# Genome assembly of 3 Amazonian *Morpho* butterfly species reveals Z-chromosome rearrangements between closely related species living in sympatry

**DOI:** 10.1093/gigascience/giad033

**Published:** 2023-05-22

**Authors:** Héloïse Bastide, Manuela López-Villavicencio, David Ogereau, Joanna Lledo, Anne-Marie Dutrillaux, Vincent Debat, Violaine Llaurens

**Affiliations:** IDEEV, Bât. 680,12, 91190 Gif Sur Yvette, France; Institut de Systématique, Evolution et Biodiversité (UMR 7205 CNRS/MNHN/SU/EPHE/UA), Muséum National d’Histoire Naturelle–CP50, 75005 Paris, France; IDEEV, Bât. 680,12, 91190 Gif Sur Yvette, France; GeT-PlaGe, Bât G2, INRAe, 31326 Castanet-Tolosan Cedex, France; Institut de Systématique, Evolution et Biodiversité (UMR 7205 CNRS/MNHN/SU/EPHE/UA), Muséum National d’Histoire Naturelle–CP50, 75005 Paris, France; Institut de Systématique, Evolution et Biodiversité (UMR 7205 CNRS/MNHN/SU/EPHE/UA), Muséum National d’Histoire Naturelle–CP50, 75005 Paris, France; Institut de Systématique, Evolution et Biodiversité (UMR 7205 CNRS/MNHN/SU/EPHE/UA), Muséum National d’Histoire Naturelle–CP50, 75005 Paris, France

**Keywords:** sympatric speciation, reinforcement, mimicry, structural variant, inversions, gene duplication, wing color pattern, evolutionary convergence: genomic divergence, karyotype

## Abstract

The genomic processes enabling speciation and species coexistence in sympatry are still largely unknown. Here we describe the whole-genome sequencing and assembly of 3 closely related species from the butterfly genus *Morpho*: *Morpho achilles* (Linnaeus, 1758), *Morpho helenor* (Cramer, 1776), and *Morpho deidamia* (Höbner, 1819). These large blue butterflies are emblematic species of the Amazonian rainforest. They live in sympatry in a wide range of their geographical distribution and display parallel diversification of dorsal wing color pattern, suggesting local mimicry. By sequencing, assembling, and annotating their genomes, we aim at uncovering prezygotic barriers preventing gene flow between these sympatric species. We found a genome size of  480 Mb for the 3 species and a chromosomal number ranging from 2*n* = 54 for *M. deidamia* to 2*n* = 56 for *M. achilles* and *M. helenor*. We also detected inversions on the sex chromosome Z that were differentially fixed between species, suggesting that chromosomal rearrangements may contribute to their reproductive isolation. The annotation of their genomes allowed us to recover in each species at least 12,000 protein-coding genes and to discover duplications of genes potentially involved in prezygotic isolation like genes controlling color discrimination (*L-opsin*). Altogether, the assembly and the annotation of these 3 new reference genomes open new research avenues into the genomic architecture of speciation and reinforcement in sympatry, establishing *Morpho* butterflies as a new eco-evolutionary model.

Key Points

- We sequenced, assembled, annotated and karyotyped the genomes of three mimetic and sympatric *Morpho* species.
- We found inversions on the Z sex chromosome that were differentially fixed between species and uncovered duplications of genes involved in vision.
- We discuss the potential implications of these findings in preventing gene flow between close sympatric species.

## Introduction

Chromosomal rearrangements are likely to play a major role in both adaptation and speciation processes [1, 2]. Inversions, for instance, can favor the emergence of adaptive syndromes by locking together coadapted allelic variations [3]. Chromosomal rearrangements have also been suggested to contribute to reproductive isolation between species by promoting divergent adaptation or by bringing together genetic incompatibilities [4]. Nevertheless, the role of structural variants in these evolutionary processes is still largely unknown. Recently developed sequencing and assembly methods now provide access to complete genomes, therefore opening the investigation of structural variation within and among species (see [5] for a review).

**Figure 1:**
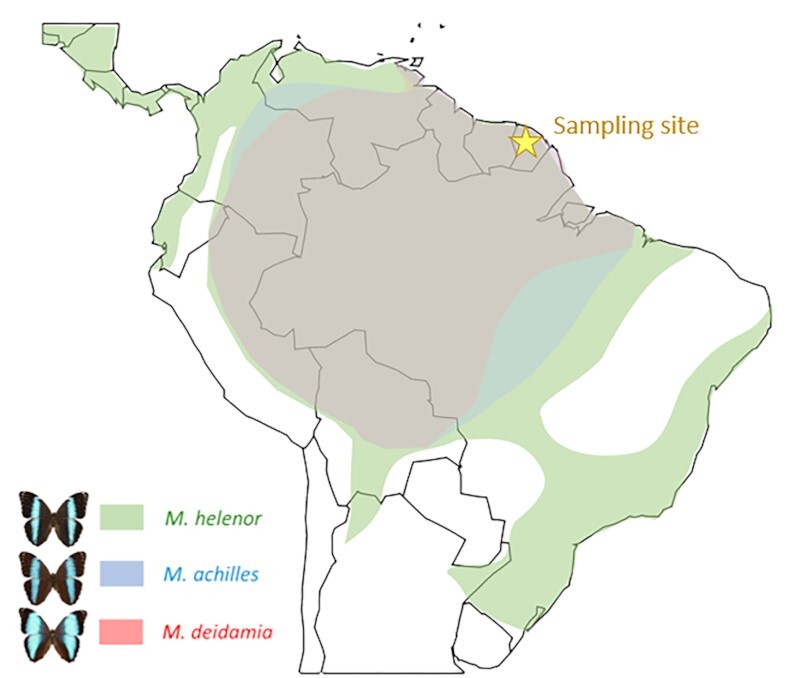
Geographical distribution of the 3 neotropical species *M. helenor* (green areas), *M. achilles* (blue areas), and *M. deidamia* (red areas). *M. helenor* has the widest distribution, from Central America to southern Brazil, while *M. achilles* and *M. deidamia* are restricted to the Amazonian basin. The 3 species are in sympatry throughout the Amazonian rainforest, including French Guiana (marked with the yellow star), where the samples studied here were collected.

Here, we focus on emblematic species of the Amazonian rainforest, the blue *Morpho*. We describe the whole-genome sequences of 3 closely related *Morpho* species living in sympatry for a large range of their geographical distribution (Fig. 1): *Morpho helenor, Morpho achilles*, and *Morpho deidamia* [6], thereby developing relevant resources to study the evolution of barriers to gene flow in sympatry. In Lepidoptera, specialization toward host plant has been shown to be a major factor affecting species diversification [7]. Such ecological specialization may favor speciation and coexistence in sympatry and may stem from the evolution of gustatory receptors enabling plant recognition by females [8].

The evolution of visual [9] and olfactory signals [10] between species may also limit gene flow between sympatric species of Lepidoptera. In the 3 *Morpho* species studied here, both males and females display conspicuous iridescent blue color patterns on the dorsal side of their wings, combined with cryptic brownish color on the ventral side [11]. Such a combination of dorsoventral patterns, associated with a fast and erratic flight, is thought to contribute to the high escape abilities from predators of these butterflies, promoting color pattern convergence between sympatric species (i.e., escape mimicry, [12]). Parallel geographic variation of dorsal wing color pattern has indeed been detected in the 3 *Morpho* species studied here, suggesting local convergence promoted by predators’ behavior [13]. Given the key role of color pattern in both sexual selection and species recognition in diurnal butterflies, such a resemblance is thought to enhance reproductive interference between sympatric species [14]. Behavioral experiments carried out in the wild revealed that males from the 3 mimetic *Morpho* species are indeed attracted by both intra- and interspecific wing patterns [15]. Despite this heterospecific attraction of males at long distances, RAD-sequencing markers revealed a highly limited gene flow between these 3 sympatric species [15]. This might be due to the differences in the timing of daily activities observed between these sympatric species, limiting heterospecific encountering [15]. This divergence in daily phenology may contribute to the initiation of speciation or to the reinforcement of prezygotic barriers to heterospecific matings.

Genetic incompatibilities may also contribute to speciation and reinforcement processes by generating postzygotic barriers. For instance, variation in chromosome numbers has been shown to correlate with speciation rate in Lepidoptera [16]. Similarly, chromosomal inversions may fuel the speciation process: by capturing genetic variations, inversions may lead to increased genetic divergence between species. Such divergence may lead to maladaption in hybrids and further limit gene flow between species living in sympatry.

By relying on both karyotype data and PacBio-Hifi sequencing, we generated de novo genome assemblies for 3 sympatric species of *Morpho* butterflies. The divergence between the 2 sister species *M. helenor* and *M. achilles* was estimated to occur about 3.91 million years (My) ago, while the divergence between these 2 sister species and *M. deidamia* was estimated to circa 16.68 My ago [17], enabling to compare the genome divergence in sympatry at different time scales. We then investigated the structural variants and variation in genes potentially contributing to prezygotic isolation among these species. We aim to shed light on the genomic processes involved in sympatric speciation and reinforcement as well as detecting chromosomal rearrangements. We also provide their mitochondrial genomes, study their transposable element (TE) contents, and annotate the genomes. These genomic resources will open new research avenues into the understanding of adaptive processes, such as convergence evolution of color pattern or divergence in visual systems, as well as speciation and coexistence of sister species in sympatry, establishing *Morpho* butterflies as a new eco-evolutionary model.

## Material and Methods

### Butterfly sampling

Males from the species *M. helenor* (*n* = 1), *M. achilles* (*n* = 4), and *M. deidamia* (*n* = 2) were caught with a handnet at the Patawa waterfall, located in the Kaw mountain area of French Guiana (GPS location: 4.54322; −52.15832) to perform DNA extractions. In these species, males typically patrol in river beds and are easy to catch, while females are more rarely encountered. We therefore focused on males only. Because in butterflies sex is controlled by a ZW sex chromosome system (females being the heterogametic sex), we were thus able to access the Z sex chromosome but not the W chromosome.

### Karyotype study

Cytogenetic techniques were applied to wild-caught males (*M. helenor, n* = 3; *M. achilles, n* = 4; and *M. deidamia, n* = 2) that were collected at the abovementioned location in 2019. Their testicles were dissected and processed shortly after capture following the protocol described in [18]. The obtained cell suspension was conserved in fixative at about 4°C. The cell spreading and staining were then performed as described in [18]. The chromosome staining relied on the Giemsa method.

### DNA extractions and genome sequencing

Live butterflies (*M. helenor, n* = 1; *M. achilles, n* = 4; and *M. deidamia, n* = 2) captured in 2021 at the same site in French Guiana were killed in the lab and their bodies immediately placed in liquid nitrogen. The DNA extraction was carried out the following day using the Qiagen Genomic-tip 100/G kit and following supplier instructions. The extracted DNA of a single male from each species was used (see Supplementary Fig. S1 for pictures of the wings of the sequenced specimens). Library preparation and sequencing were performed at the GeT-PlaGe core facility (INRAe Toulouse) according to the manufacturer’s instructions, “Procedure and Checklist Preparing HiFi SMRTbell Libraries Using SMRTbell Express Template Prep Kit 2.0.” At each step, DNA was quantified using the Qubit dsDNA HS Assay Kit (Life Technologies). DNA purity was tested using the nanodrop (Thermo Fisher) and size distribution and degradation assessed using the Femto pulse Genomic DNA 165-kb Kit (Agilent). Purification steps were performed using AMPure PB beads (Pacific Biosciences). Next, 15 μg DNA was purified and then sheared at 15 kb (speed 31 and 32) with the Megaruptor3 system (Diagenode). Using the SMRTbell Express Template prep kit 2.0, a single-strand overhang removal, a DNA and END damage repair step was performed on 10 μg of sample. Blunt hairpin adapters were then ligated to the library, which was treated with an exonuclease cocktail to digest unligated DNA fragments. A size selection step using a 10-kb cutoff was performed on the BluePippin Size Selection system (Sage Science) with the “0.75 percent DF Marker S1 6-10 kb vs3 Improved Recovery” protocol. Using Binding kit 2.2 and sequencing kit 2.0, the primer V5 annealed and polymerase 2.2 bounded library was sequenced by diffusion loading onto 1 SMRTcells per sample on a SequelII instrument at 80 pM with a 2-hour preextension and a 30-hour movie.

### *K*-mer analysis, genome size, and heterozygosity estimation

We used Jellyfish (v.2.3.0) [19] to perform a *k*-mer analysis on each PacBio dataset with a *k*-mer size of 21. For each dataset, *k*-mers were counted and aggregated (jellyfish count option) and histograms were generated using the “-histo” command. The resulting histograms allowed the estimation of genome length and heterozygosity with GenomeScope version 2.0 [20] using the web application.

### Nuclear and mitochondrial genome assembly

For the assembly of the nuclear genomes, we compared 3 long-read assembly tools: IPA-Improved Phased Assembler (v1.0.3-0) (https://github.com/PacificBiosciences/pbipa), Flye (v2.9) [21], and Hifiasm (v0.16.1 with the option -l3 to purge all types of haplotigs in the most aggressive way) [22]. For each assembler, we estimated basic assembly statistics such as scaffold count, contig count, and N50 using the “stats.sh” program from the BBMap v38.93 package [23]. The completeness of each assembly was assessed using BUSCO v5.2.2 and MetaEuk for gene prediction against the $lepidoptera\_odb10$ database [24]. We retained the Hifiasm assembly because it had the highest BUSCO score, the highest contiguity (N50), and the longest contig. Despite the high level of purging performed by Hifiasm, the species (*M. helenor* and *M. achilles*, respectively) retained a high level of duplicates in the BUSCO score. To remove false haplotypic duplications in these 2 species, we used Purge_dups v1.2.5, setting the cutoffs manually (with calcuts -l 5 -m 33 -u 135 for *M. helenor* and calcuts -l 10 -m 45 -u 145 for *M. achilles*) [25]. The completeness of the purged genomes was then reassessed using BUSCO.

The mitochondrial genome of each species was assembled and circularized using Rebaler (https://github.com/rrwick/Rebaler) directly from the PacBio Hifi reads and using the mitochondrial genome of the closely related species *Pararge aegeria* as a reference.

### Annotation of repetitive regions

The annotation of repetitive regions in the 3 species was performed following 2 main steps. First, we used RepeatModeler v2.0.2a [26] with the option -s (slow search) and -a (to get a.align output file) to create de novo libraries of repetitive elements for each species. The library was then used to hardmask the corresponding genome assembly using RepeatMasker 4.1.2.p1 [26]. A summary of the repeated elements was generated with the script “buildSummary.pl” included in RepeatMasker.

### Genome annotation

Each of the 3 genomes was independently annotated using MAKER v2.31.10 [27], following the protocol given in [28]. In short, MAKER is usually run several times successively and uses the gene models generated in 1 round to train ab initio gene predictors and improve the initial gene models in the next round (see below). We used the abovementioned hardmasked genomes and carried out their annotation using the proteomes of 3 closely related species, namely, *P. aegeria* [29], *Maniola hyperantus* [30], and *Bicyclus anynana* [31]. For each species, the output files were merged into a gff3 file that was then used to generate the necessary files to train SNAP (version 2006-07-28), an ab initio gene-finding program [32]. A second run of MAKER with the abovementioned gff3 file and the .hmm file provided by SNAP resulted in a second gff3 file that was used to train SNAP a second time. A third round of MAKER with the second gff3 and .hmm files was followed by the training of Augustus (3.3.3), another gene prediction tool [33], with the third gff3 file. A final round of MAKER with the third gff3 file and the files generated by Augustus led to the fourth and last gff3 file, containing all the genome features for each species.

Protein-Protein BLAST 2.9.0+ (-evalue 1e-6 -max_hsps 1 -max_target_seqs 1) was then used to assess putative protein functions in each *Morpho* species by comparing the protein sequences given by MAKER to the protein sequences from the annotated genomes of *Maniola jurtina* [29], *P. aegeria* [29], *B. anynana* [31], and *Spodoptera littoralis* specifically for the detection of OR (olfactory receptor) sequences [34]. We used BUSCO to assess the completeness of the proteome with the protein mode and the $lepidoptera\_odb10$ database on the annotated gene set produced by MAKER [24].

### Phylogenetic analysis

To specifically compare the exon sequences of the opsins detected in the *Morpho* genomes to the opsins described in other Lepidoptera, we retrieved the coding sequences of opsins from NCBI and used the software Mega v.11 [35] to build a maximum likelihood tree and compute the associated bootstrap values.

Regarding the OR repertoire in the 3 *Morpho* species, we curated the sequences obtained by blast comparison of the MAKER-annotated genes on the reference genome of *S. littoralis*, as a number of sequences showed incorrect lengths (<300 or >500 amino acids). We used exonerate version 2.4.0 [36] with the options -maxintron 2000 independently in each *Morpho* species. The exonerate alignment files and the assemblies were used with InsectOR (http://caps.ncbs.res.in/gws_ors/), a website specifically designed to help predict OR genes from insect genomes, with the option HMMSEARCH against 7tm_6 [37, 38]. The sequences uncovered with insectOR for each *Morpho* species were aligned with the ORs of *S. littoralis* using MAFFT [39], and we generated a maximum likelihood phylogenetic tree with IQ-TREE version 2.2.0 [40] with the options -bb 1000 and -nt AUTO.

### Synteny and rearrangement detection

To assess variation in chromosome-scale synteny, we compared the assemblies of each *Morpho* to the assembly of *M. jurtina*, the closest relative of *Morpho* with a karyotype of 29 chromosomes and for which a high-quality chromosome-level assembly (based on N50 values and BUSCO score, accession ID GCF_905333055.1) is available [29]. We used MUMmer 3.23 [41] to align the masked assembled genomes of *M. helenor, M. achilles*, and *M. deidamia* to the *M. jurtina* genome. The output produced by MUMmer is an ASCII delta file that was then filtered and parsed using the utility programs delta-filter and show-coords from MUMmer. Synteny was visualized with the MUMmer results in R with the packages circlize v 0.4.12 [42] and Paletteer (https://github.com/EmilHvitfeldt/paletteer) using the Rscript from [43] described here: https://github.com/bioinfowheat/Polygonia_calbum_genomics/blob/7c75aac624157faa3ab229e3fc1e0e315302194d/synteny/circlePlot_nucmerOutput.R, removing short contigs, short alignments (less than 200 bp), and low-identity alignments (less than 90% identity).

In order to detect potential genome rearrangements between *Morpho* and closely related species, we estimated the whole-genome collinearity between the *Morpho* assemblies and 5 closely related Nymphalidae species whose genomes exhibit good-quality assemblies in the NCBI genome database: *M. jurtina* (GCA_905333055.1), *P. aegeria* (GCA_905333055.1), *Erebia ligea* (GCA_923060345.2), *Melanargia galathea* (GCA_920104075.1), and *Lasiommata megera* (GCA_928268935.1) using D-GENIES [44]. Paired alignments between a *Morpho* species and 1 Nymphalidae species were performed using the minimap2 aligner [45] in D-GENIES, treating each *Morpho* species genome as the query and the Nymphalidae species genome as the target reference. We also used D-GENIES to pair-compare the genomes of the 3 *Morpho* species. As D-GENIES revealed differences between *Morpho* species in the contig corresponding to the Z chromosome (see Results), we used SyRI [46] to study in detail the rearrangements in the sequences of this contig between the 3 species. We generated paired alignments of the Z contig with minimap2 and ran SyRI with the option -c on.sam files. SyRI requires that the 2 compared genomes represent the same strand, and in the case of *M. achilles*, the orientation of the sequence produced by Hifiasm was complementary to the sequences of *M. helenor* and *M. deidamia*. We then reverse-complemented this sequence in order to make the alignments. All the genomic structures predicted by SyRI were plotted using plotsr [47].

## Results

### Comparing karyotypes between species

First, we characterized the karyotypes of the 3 studied species (see Supplementary Fig. S2 to visualize the chromosomes). In *M. helenor*, the detected number of diploid chromosomes ranged from 54 to 56 in the different replicates of mitoses, with a discreet mode at 2*n* = 56. This variation is probably due to technical difficulties. The presence of *n* = 28 bivalents in metaphase confirmed the diploid number of 2*n* = 56 chromosomes. In *M. achilles*, 4 specimens had the same modal chromosome counts: mitoses, 2*n* = 56 chromosomes; pachynema, *n* = 28 bivalents; metaphase I, *n* = 28 bivalents; and metaphase II, *n* = 28 chromosomes with 2 chromatids. Surprisingly, the karyotype of the last male was quite different, with a modal number of 84 mitotic chromosomes. Interestingly, there was the same number (*n* = 28) of elements as above at the pachynema stage, indicating that they were trivalents. They were thicker than bivalents, and a more careful analysis showed the recurrent asynapsis of 1 of the 3 chromosomes (Supplementary Fig. S3). No “normal” metaphase I or II was observed. It was concluded that this specimen was triploid with 3*n* = 84 and probably sterile. In *M. deidamia*, the diploid chromosome number had a discreet mode of 2*n* = 54, suggesting a slightly smaller number of chromosome pairs (*n* = 27) in this more distantly related species. Our result is consistent with the modal number of chromosomes in the Morphinae (*n* = 28) described in previous karyotypic studies conducted in 8 *Morpho* species [48], where the reported number of chromosomes was also *n* = 28 for both *M. helenor* and *M. achilles*.

GenomeScope analyses suggested very high levels of heterozygosity for the 3 species (Table 1). In all of them, the N50 and contig sizes were generally larger in the assemblies produced by Hifiasm than in IPA and Flye assemblies (see Supplementary Table S1). The BUSCO scores revealed a very high percentage of duplicated sequences, especially in the assemblies produced by IPA and Flye. The use of purge_dups strongly reduced the number of duplicates, the estimated size of the genome, and the number of final contigs (see Supplementary Fig. S4 and Supplementary Table S1). Hifiasm and the posttreatment with Purge_dups v1.2.5 gave an assembly of 143 contigs for *M. helenor* (size of the longest contig: 42,411,663 bp), of 32 contigs for *M. achilles* (size of the longest contig: 24,854,087 bp), and of 58 contigs for *M. deidamia* (size of the longest contig: 22,518,629 bp) (Supplementary Table S1). The Rebaler pipeline identified a circular mitochondrial genome of 15,336 bp for the species *M. helenor*, 15,340 bp for *M. achilles*, and 15,196 bp for *M. deidamia*.

**Table 1:** Genome heterozygosity estimated with GenomeScope and Genome statistics for the assemblies of 3 *Morpho* species using different computational methods. Assemblies were purged using purge_dups. Statistics were obtained with BBMap. The assembly produced with Hifiasm for the individual *M. deidamia* was not purged with purge_dups as BUSCO results on the preliminary assembly revealed a very low duplicate content.

	*M. helenor*	*M. achilles*	*M. deidamia*
Heterozygosity (%)	3.35	2.78	1.68
Assembly method			
Hifiasm			
Total contigs	143	32	58
Genome size	470.254 Mb	478.514 Mb	489.914 Mb
N50	12 Mb	12 Mb	13 Mb
IPA			
Total contigs	128	56	47
Genome size	473.620 Mb	493.177 Mb	481.177 Mb
N50	17 Mb	14 Mb	13 Mb
Flye			
Total contigs	134	114	291
Genome size	466.515 Mb	477.638 Mb	484.463 Mb
N50	21 Mb	20 Mb	34 Mb

#### Annotation of repetitive region

In each of the 3 species of *Morpho*, we annotated around 50% of the genome as repeated elements (Supplementary Fig. S5). In *M. helenor*, 241,166,073 bp (51.28% of the genome) corresponded to repeated elements, 261,488,514 bp (54.65% of the genome) in *M. achilles*, and 255,779,512 bp (52.75% of the genome) in *M. deidamia*. The repetitive elements categories are shown in Supplementary Fig. S5. For the 3 species, long interspersed nuclear elements (LINEs) accounted for the largest percentage (between 13.53% and 17.22% ) of the repeated elements in the genomes.

### Genome annotation

We recovered 12,651, 12,978, and 12,093 protein-coding genes in the genomes of *M. helenor, M. achilles*, and *M. deidamia*, respectively. These values were comparable to what was found in *Maniola hyperantus* (13,005 protein-coding genes) and *P. aegeria* (13,515 protein-coding genes) but were lower than in *M. jurtina* (13,777 protein-coding genes) and *B. anynana* (14,413 protein-coding genes). BUSCO results for the proteome and transcriptome are presented as supplementary material (see Supplementary Figs. S5 and S6). In order to assess if the annotations were complete, we estimated in each species the percentage of proteins with a Pfam domain as this value has been found to vary between 57% and 75% in eukaryotes [49]. This value ranged from 65.50% in *M. achilles* to 71.32% in *M. helenor* with an intermediate value of 70.42% in *M. deidamia*, thus showing that the annotations were of good quality. Proteome completeness using BUSCO was also high. From a set of 5,286 single-copy orthologs from the Lepidoptera lineage, the proteome completeness varied between 69% and 79% depending on the species (Supplementary Fig. S5). We were thus able to further investigate gene families that could be involved in prezygotic isolation through duplication or loss events. This includes genes having a role in vision (*L opsin*) but also chemosensory genes such as odorant and gustatory receptors that reflect the degree of species specialization.

#### Duplications in opsin genes

**Figure 2:**
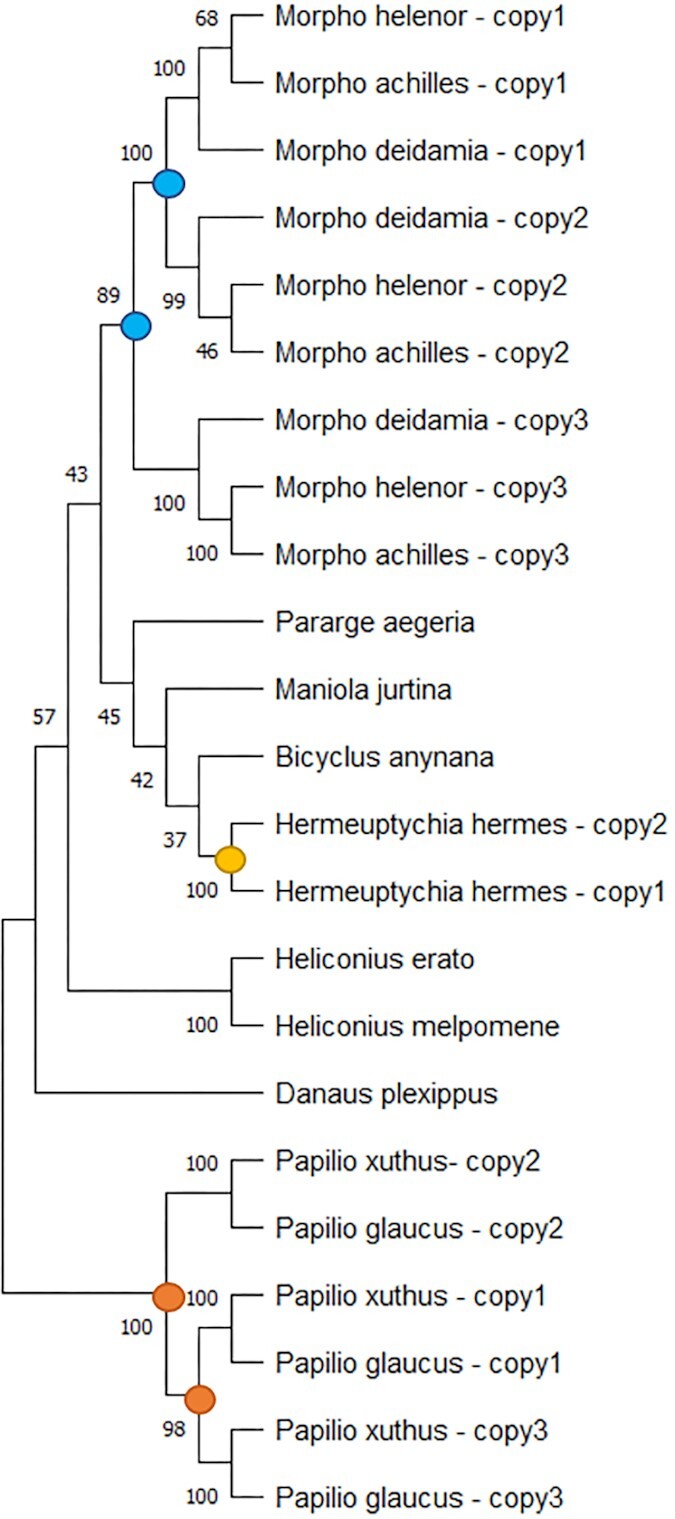
Maximum likelihood tree of L-opsin exon sequences detected in the genomes of *M. helenor, M. achilles*, and *M. deidamia* and other butterflies species, with bootstrap values. The colored dots indicate the putative locations of the duplication events on the tree: the putative origin of duplications of the L-opsin observed within the genus *Morpho* appear in blue, while the duplications that occurred in the *Hermeuptychia hermes* clade and in the *Papilio* clade appear in yellow and orange, respectively.

Vision in butterflies notably relies on opsins, for which 3 major types of molecules have been described depending on their wavelength of peak absorbance: in the ultraviolet (UV, 300–400 nm), blue (B, 400–500 nm), and long-wavelength (L, 500–600 nm) part of the visible spectrum. Opsins are encoded by UV, B, and L opsin genes. We investigated the number of copies for each opsin gene in the 3 *Morpho* species. We consistently found 1 copy of the UV opsin gene and 2 copies of the B opsin genes in the 3 *Morpho* species. Duplications of L opsin were observed in *M. achilles, M. deidamia*, and *M. helenor*. In the other reference genomes, *M. jurtina, B. anynana*, and *P. aegeria*, a single copy of the UV opsin gene, the B opsin gene, and the L opsin gene was found. By comparing the L opsin sequences using a maximum likelihood tree based on the exon sequences (Fig. 2), we showed that the duplications observed in *Morpho* butterflies probably occurred independently from previously described duplications that happened in other clades of Lepidoptera. The phylogenetic relationships between the copies in the 3 species reveal that the duplications observed in the 3 *Morpho* species probably occurred before their speciation (Fig. 2). The detection of the different copies in different species within the *Morpho* genus and in closely related genus is now required to precisely characterize the evolutionary origin of these duplications.

#### Odorant and gustatory receptors

In order to estimate the number of *OR* and *GR* genes in the 3 *Morpho* species, we blasted our MAKER-annotated genes on the reference genome of *S. littoralis*. In this moth species, 60 *OR* and 16 *GR* genes were curated [50]. Interestingly, we recovered only 31 *OR* genes including Orco in *M. helenor*, 32 in *M. achilles*, and 36 in *M. deidamia*, while we found 14 *GR* genes in *M. helenor* and 16 in *M. achilles* and *M. deidamia*. With insectOR, we found 36 OR genes including Orco in *M. helenor*, 37 in *M. achilles*, and 38 in *M. deidamia*, confirming the major loss of ORs in our 3 *Morpho* species. For comparison, we blasted against the same reference genome of *S. littoralis* the annotated sequences of the 3 outer Lepidopteran species used in the previous analyses and uncovered a much higher number of *OR* and *GR* genes with 61 *OR* and 28 *GR* in *M. jurtina*, 60 *OR* and 35 *GR* in *B. anynana*, and 50 *OR* and 20 *GR* in *P. aegeria*, respectively. The drastic reduction of chemosensory receptors, particularly in the number of *OR* genes in the 3 *Morpho* species, could potentially reflect a higher degree of specialization to their respective biochemical environment. A phylogenetic analysis of *Morpho* ORs along with those of *S. littoralis*, the sole Lepidopteran species for which a considerable number of ORs were functionally deorphanized and divided into 3 chemical classes (aromatics, terpenes, and aliphatics) as described in [34], showed that the loss of ORs in *Morpho* was not clustered around a particular set of genes (Supplementary Fig. S9). Further functional characterization coupled with precise ecological investigations are therefore needed to understand the loss of ORs in the *Morpho* genus.

### Synteny and rearrangement detection

#### Conserved synteny with other Lepidoptera species

**Figure 3:**
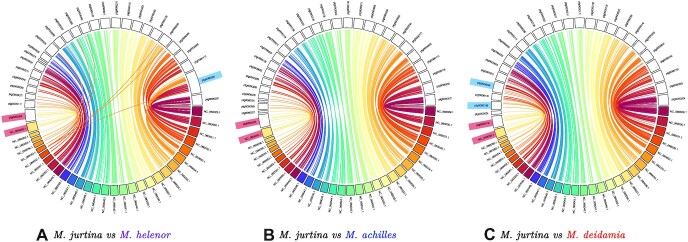
Synteny between the chromosome-assembled genome of *M. jurtina* (colored chromosomes) and the genome assemblies of the species *M. helenor* (A), *M. achilles* (B), and *M. deidamia* (C). Equivalent chromosomes/contigs are linked by same color ribbons. Chromosome Z for each species is labeled in red. Single chromosomes in *Morpho* that are not assigned to a single chromosome in *M. jurtina* are labeled in blue.

We found a high concordance between the *n* = 29 chromosomes of *M. jurtina* and the contigs of the 3 *Morpho* species (Fig. 3). The MUMmer alignment and the postalignment treatment to remove short contigs and low-identity alignments reduced the assembly to 27 contigs containing 97% of the total genome for *M. helenor* (removing 117 short contigs from the original assembly), 29 contigs (98% of the genome) for *M. achilles* (3 contigs removed), and 27 for *M. deidamia* (31 contigs removed) (Fig. 3).

The synteny plot between *M. helenor* and *M. jurtina* showed 27 contigs for *M. helenor*, 1 contig less than expected based on its karyotype of *n* = 28. In the plot, 1 single contig (ptg000028l) was assigned to 2 different chromosomes from the *M. jurtina* assembly (chromosomes 2 and 6, NC_060030.1 and NC_060034.1). Contig ptg000028l is twice the size of any other contig found in the 3 *Morpho* species analyzed here. Based on the differences between the number of contigs and the karyotype and the unexpectedly big size of the contig ptg000028l, we believe the difference in chromosome number between *M. jurtina* and *M. helenor* can be explained by an overassembly of the genome of *M. helenor* by Hifiasm, which assigned 1 single contig to 2 different chromosomes from the *M. jurtina* assembly (Fig. 3). In *M. deidamia*, the Hifiasm assembly showed a single contig, ptg000008l (size 20.29 Mb), containing chromosomes NC_060051.1 and NC_060052.1 (sizes 10.05 Mb and 9.43 Mb, respectively) from *M. jurtina*. Because the number of contigs recovered for this species is in accordance with the karyotype of *n* = 27, the differences between *M. deidamia* and *M. jurtina* suggest that in this case, chromosomes NC_060051.1 and NC_060052.1 in *M. jurtina* may have fused to form contig ptg000008l in *M. deidamia*. Other rearrangement in this species compared to *M. jurtina* seems to be the contig ptg0000161l, which appears to contain small portions of chromosomes NC_060054.1 and NC_060055.1 from *M. jurtina*.

For the 3 *Morpho* species, we were able to identify a single contig corresponding to the chromosome Z (NC_060058.1) in *M. jurtina* (contig ptg000030l in *M. helenor*, contig ptg000024l in *M. achilles*, and contig ptg000019l in *M. deidamia*).

We also found a high level of collinearity between the genomes of the 3 *Morpho* species and the 5 Nymphalidae species used for comparisons. The alignment between *M. jurtina* and the 3 *Morpho* species (Fig. 3) was very similar to the alignments obtained for the other Nymphalidae (Supplementary Fig. S6) and confirmed that the assembly of the genome of *M. helenor* by Hifiasm might have merged together 2 chromsomes: the single contig ptg000028l was scattered into 2 chromosomes in the other Nymphalidae. Although collinearity was generally high, we detected some putative inversions located in regions that varied among pairs for the 3 *Morpho* species in comparison with the Nymphalidae (see Supplementary Fig. S6). Interestingly, the contig corresponding to the chromosome Z was the only one consistently showing inversions in the pairwise genome-wide alignments (see Supplementary Fig. S6).

#### Inversions in the Z chromosome between the 3 sympatric *Morpho* species

**Figure 4:**
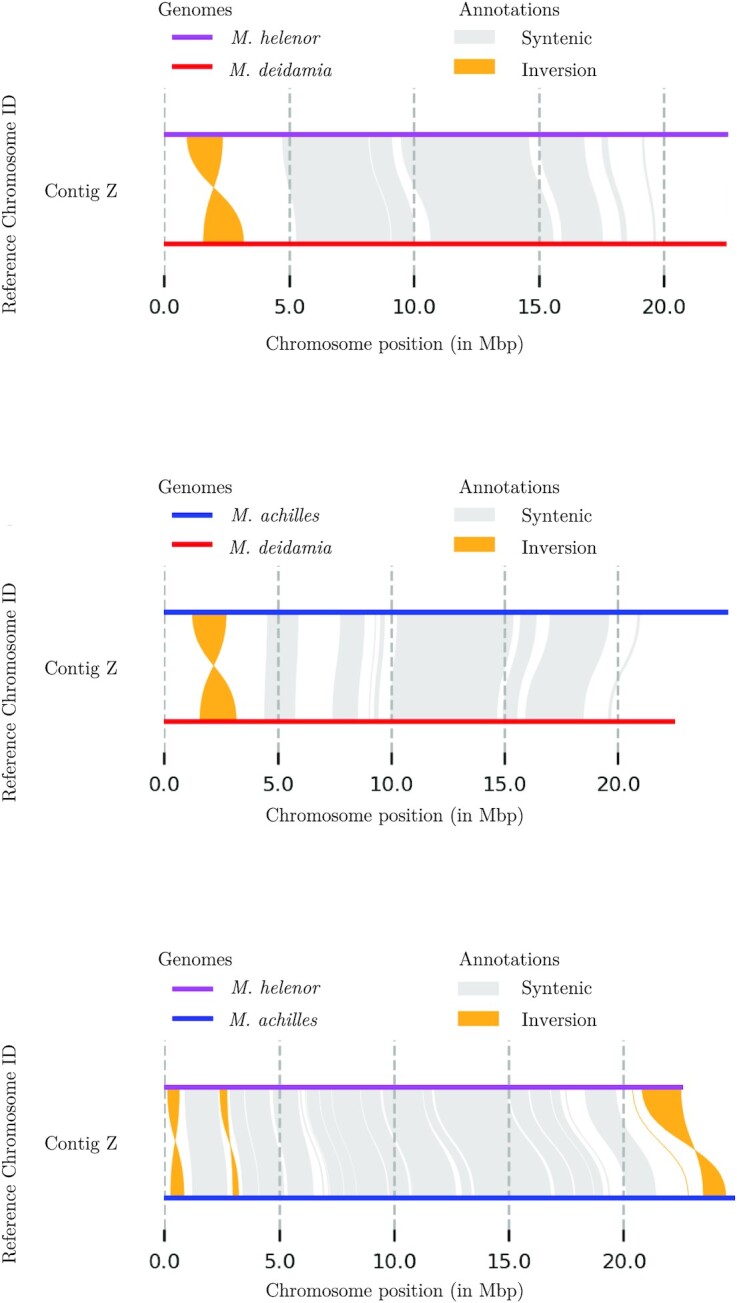
Synteny and rearrangement (SyRI) plot of the paired comparisons for the Z contig between the 3 *Morpho* species. Upper figure: *M. helenor* and *M. deidamia*; middle: *M. deidamia* and *M. achilles*; lower: *M. helenor* and *M. achilles*.

Pairwise whole-genome alignments of the 3 *Morpho* species showed a very high similarity between genomes (see Supplementary Fig. S7). The only contig that differed between species was the one corresponding to the Z chromosome. SyRI identified 1 inversion of 1.6 Mb between *M. helenor* and *M. deidamia*, 5 inversions (comprising 1 of more than 1.8 Mb) between *M. helenor* and *M. achilles*, and 2 between *M. deidamia* and *M. achilles* with 1 of 1.6 Mb (Fig. 4). Interestingly, the inversion found in *M. deidamia* when compared to *M. achilles* or *M. helenor* has the same size and is located in exactly the same position of the chromosome (from bp 1567583 to 3192401), suggesting that this inversion is ancestral to the speciation of *M. achilles* and *M. helenor*. In the case of *M. achilles* versus *M. helenor*, 2 inversions were found flanking the site of the putative ancient inversion, and a bigger inversion was found at the end of the chromosome (Fig. 4).

## Discussion

### Assembly of heterozygous Lepidoptera genomes with a high proportion of repeated elements

We generated de novo, reference-quality genome assemblies for 3 emblematic species of Amazonian butterflies: *M. helenor, M. achilles*, and *M. deidamia*. Our results indicate genome sizes comprised between 470 Mb and 489 Mb, similar to most of the closely related Nymphalidae species sequenced so far, for example, *B. anynana* (475 Mb), *P. aegeria* (479 Mb), or *M. jurtina* (429 Mb). This is also close to the 479 Mb estimated from phylogenetic comparison using the taxon-centered database “Genomes on a Tree” (GoaT) [51]. The final number of contigs within each of the 3 species ranged from 27 to 29, close to the number of chromosome pairs observed in our cytogenetics study. The number of chromosomes found in those French Guiana samples (i.e., in the subspecies *M. helenor helenor* and *M. achilles achilles*) is consistent with those found in other subspecies of both species in previous studies [48]. The available sequenced species of Nymphalidae that are closely related to the genus *Morpho* also generally show 29 pairs of chromosomes (28 autosomes, plus Z and W sex chromosomes), which is close to the chromosomal numbers observed in the 3 *Morpho* species studied here. The mapping between the assemblies of *Morpho* species to the chromosome-level assembly of *M. jurtina* and the posttreatment to eliminate small contigs allowed us to identify between 27 and 29 contigs in *Morpho* that were homologous to *M. jurtina* chromosomes, including the contig corresponding to the Z chromosome. This suggests a high conservation of chromosomal synteny among closely related Nymphalidae species, which is consistent with the high level of synteny observed throughout the whole Lepidoptera clade [52]. In the 3 species, genome heterozygosity was very high (from 1.68% in *M. deidamia* to 3.35% in *M. helenor*), and heterozygosity presents a major challenge in de novo assembly of diploid genomes. Indeed, levels of heterozygosity of 1% or above are considered “moderate to high,” and most assemblers struggle when 2 divergent haplotypes are sequenced together, as heterozygosity may impair the distinction of different alleles at the same locus from paralogs at different loci [53]. Then, final assemblies of heterozygous genomes are expected to be of poor quality, highly fragmented, and containing redundant contigs [54]. Hifiasm generated the most completely haplotype-resolved assemblies; nevertheless, the level of heterozygosity clearly impacted the quality of the assemblies, and a posttreatment to remove duplicated sequences was necessary for the 2 most heterozygous genomes (*M. helenor* and *M. achilles*), showing the difficulty that heterozygosity still imposes on long-read heterozygosity-aware assemblers. Such a high heterozygosity has been observed in other genomes of Lepidoptera [31] and can be a signature of high effective population sizes. The wide Amazonian distribution of these species and their flight activity could contribute to such a high level of genetic diversity within the population, because elevated dispersal contributes to increased gene flow within each species throughout their geographic range. Our results also showed that around 50% of the genomes of the sequenced *Morpho* were composed of repeated elements, a very high proportion as compared to other genomes of Lepidoptera. In Lepidoptera, TE content has been found to be correlated with genome size [55], but in the case of the 3 *Morpho* species studied here, the repeat content is higher than for other species with similar genome sizes such as the *Bombyx mori* moth, with a genome size estimated at 530 Mb and a TE content of 35% [56] or the more closely related species *B. anynana* with a genome size of 475 Mb and a repeat content of 26% [31].

### Structural variations between genomes of sympatric species

The karyotype and assembly analyses suggest some differences in chromosome number between the 3 sympatric *Morpho* species studied here, particularly between *M. deidamia* (27 chromosome pairs) versus *M. helenor* and *M. achilles* (28 chromosome pairs). Differences in chromosome numbers and other chromosomal rearrangements may strongly affect reproductive barriers. Two groups of models have been proposed to explain how chromosomal rearrangements prevent gene flow and contribute to species maintenance and speciation. First, hybrid-sterility models suggest reduced fertility or viability in individuals heterozygous for chromosomal rearrangements. These models are considered inconsistent and difficult to evaluate [4]. More recently, suppressed-recombination models propose that chromosomal rearrangements permit speciation in sympatry because they reduce recombination between chromosomes carrying different rearrangements [4]. Indeed, in Lepidoptera, differences in chromosome number are proposed to be an important mechanism leading to species diversification in *Agrodiaetus, Erebia*, and *Lysandra* butterflies [57–59].

Besides differences in chromosome numbers, we systematically found inversions in the contig corresponding to the Z chromosome when comparing the genomes of *Morpho* to the other Nymphalidae and between the 3 different *Morpho* species. Inversions are also a type of chromosomal rearrangement known to occur throughout evolution and are considered an important mechanism for speciation, particularly for species living in sympatry [1, 4]. Empirically and theoretically, it has been suggested that inversions may have contributed to speciation in sympatry in different groups of animals. In 2 ascidian species of the genus *Ciona* and in insects like *Drosophila*, inversions may promote speciation by reduction of the fitness or by causing sterility of heterozygotes [60, 61]. In the *Anopheles gambiae* species complex, inversions may allow for ecotypic differentiation and niche partitioning, leading to different sympatric and genetically isolated populations [62]. In groups like paserine birds where sexual differentiation is controlled by a ZW sex chromosome system (females being the heterogametic sex), inversions in the Z chromosome in particular seem to explain speciation in sympatry between close species. Cytological data show that across the Passeriformes, the Z chromosome has accumulated more inversions than any other autosome and that the inversion fixation rate on the Z chromosome is 1.4 times greater than the average autosome. Interestingly, inversions on the Z chromosome are significantly more common in sympatric than in allopatric closely related clades [63, 64].

In Lepidoptera, the role of inversions in speciation in sympatry has been studied in the species *Heliconius melpomene* and *Heliconius cydno*, 2 sympatric species that can hybridize (although rarely) in the wild [65]. The analyses of the genomic differences between the 2 species showed some small inversions (less than 50 kb), and there was no evidence for a reduction of recombination in hybrids, suggesting that in this case, inversions were not involved in the maintenance of the species barriers, and other processes such as strong mate preference could prevent hybridization in the wild [65]. In the *Morpho* species studied here, however, we found inversions between *Morpho* Z chromosomes that were longer than 1.5 Mb. Models suggest that to be associated with adaptive traits or species barriers, inversions should typically be megabases long in order to be fixed in populations [65]. The position of the inversion in the Z contig when comparing *M. helenor* or *M. achilles* to *M. deidamia* is at the exact same place in *M. deidamia*’s genome, suggesting that this specific inversion likely occurred before the speciation between *M. achilles* and *M. helenor*. When comparing *M. helenor* to *M. achilles*, we found 2 different smaller inversions that are not found in *M. deidamia* and that are close to the putative ancestral inversion region, suggesting that these 2 smaller inversions could have appeared after the speciation between *M. achilles* and *M. helenor*. At the moment, we do not know if the inversions segregate at different frequencies in the *Morpho* populations or if they are fixed. Population analyses are needed to answer this question and to enlighten what evolutionary forces could be acting to maintain them. The copy number variation detected in genes involved in color perception (i.e., *L opsin*) may also play a significant role in reproductive isolation in these sympatric species. For instance, the 3 copies of L opsins found in the *Papilio* genus (Fig. 2) have been found to also show subfunctionalization and neofunctionalization [66]. The duplication followed by genetic divergence observed in these 3 mimetic *Morpho* species may improve their visual discrimination capacities and facilitate species recognition, therefore reinforcing barriers to gene flow in sympatry. Genes potentially involved in color pattern variations (e.g., *bric*-*a*-*brac* or *bab*) may also play a role in prezygotic isolation, but they were not thoroughly investigated here as their functional evolution involves changes in regulatory sequences rather than events of duplication or gene loss [67]. Interestingly, an orthologous search of the putative proteic sequences of each *Morpho* species against those of *M. jurtina* allowed us to uncover different copy numbers of the gene *bric-a-brac*, which play a significant role in differences of UV iridescence between males of 2 incipient species of sulfur butterflies [68]. The copy responsible for the presence/absence of UV iridescence is located on the Z chromosome, and in the 3 *Morpho* species, we found 1 or more copies of *bric-a-brac* on the contigs that correspond to the Z chromosome: *M. deidamia* had 1 copy of *bric-a-brac*, while *M. helenor* and *M. achilles* displayed 2 copies of this gene. It seems, however, that the second copy in *M. helenor* and *M. achilles* corresponds to truncated copies of *bric-a-brac*. While this is certainly the sign of an ancient duplication followed by a pseudogenization event, this could lead to further investigations of putative functions of the truncated copies. It is worth noting that variations in the number of *bab* copies were also observed in the 3 reference genomes used for the blast: *M. jurtina* had 2 copies on the Z chromosome (including a truncated copy), *B. anynana* had only 1 copy, and *P. aegeria* had none. The investigation of gene levels of polymorphism on the Z chromosomes would also be of great interest as genes linked to the Z chromosome are often among the most divergent between closely related species [69].

Altogether, the assembly and annotation of these 3 mimetic species of *Morpho* butterflies reveal differences in chromosome numbers, the presence of several Mb-long inversions in the Z chromosome, and copy number variation and genetic divergence among copies of genes that may play a significant role in reproductive isolation. Our study thus open new avenues into the investigation of the ecological and genomic factors involved in sympatric speciation and its reinforcement.

## Supplementary Material

giad033_GIGA-D-22-00294_Original_Submission

giad033_GIGA-D-22-00294_Revision_1

giad033_GIGA-D-22-00294_Revision_2

giad033_Response_to_Reviewer_Comments_Original_Submission

giad033_Response_to_Reviewer_Comments_Revision_1

giad033_Reviewer_1_Report_Original_Submission Charlotte Julie Wright, MPhil -- 12/8/2022 Reviewed

giad033_Reviewer_2_Report_Original_Submission Niklas Wahlberg -- 12/15/2022 Reviewed

giad033_Supplemental_Files

## Data Availability

Fastq files, genome assemblies, assembly methods, and collection information were uploaded at the ENA website (https://www.ebi.ac.uk/ena/browser/home) under the project number PRJEB56642. Genome accession numbers are ERZ14213098 for *M. helenor*, ERZ14213099 for *M. achilles*, and ERZ14213100 for *M. deidamia*. Other data further supporting this work are openly available in the *GigaScience* repository, GigaDB [70–72].

## Additional Files

**Supplementary Table S1**. Genome statistics for the assemblies of 3 *Morpho* species before the use of Purge_dups and using different assemblers. Statistics were obtained with BBMap.

**Supplementary Fig. S1**. Pictures of dorsal and ventral sides of the wings of the sequenced specimens of *M. helenor, M. achilles*, and *M. deidamia* sampled in French Guiana.

**Supplementary Fig. S2**. Pictures of caryotypes obtained from specimens of *M. helenor, M. achilles*, and *M. deidamia* sampled in French Guiana. Note that the chromosome number differs between *M. deidamia* and the other 2 species.

**Supplementary Fig. S3**. Picture of the putative triploid caryotype obtained from a specimen of *M. achilles*. There was the same number (*n* = 28) of elements as in the other *M. achilles* individuals at the pachynema stage. However, these elements were thicker than bivalents, and a more careful analysis showed the recurrent asynapsis of 1 of the 3 chromosomes (see arrows).

**Supplementary Fig. S4**. BUSCO assessment of the genome assembly completeness of *Morpho* species assembled using IPA, Flye, and Hifiasm. Results correspond to the BUSCO score before (top) and after (bottom) the use of purge_dups.

**Supplementary Fig. S5**. BUSCO analysis of gene annotation of *Morpho* species. BUSCO was run in “protein” mode on the annotated gene set produced by MAKER.

**Supplementary Fig. S6**. Proportion of each class of transposable elements (TEs) in the assembled genomes of the 3 *Morpho* species.

**Supplementary Fig. S7**. D-GENIES dot plots comparing the genome assemblies of 3 *Morpho* species against the assembly of the species *M. jurtina*. Chromosomes of *M. jurtina* are on the x-axis while the y-axis shows the scaffolds of (upper) *M. helenor*, (middle) *M. achilles*, and (bottom) *M. deidamia*. The alignment between the Z chromosome of *M. jurtina* and *Morpho* corresponds to the square in the upper right corner. Here we show only the plot between *Morpho* and *M. jurtina*; comparisons with 4 other species of Nymphalidae are available upon request.

**Supplementary Fig. S8**. D-GENIES dot plots comparing the genome assemblies of pairs of *Morpho* genomes. The red arrows indicate the inversions (in the scaffolds corresponding to the Z chromosome).

**Supplementary Fig. S9**. Phylogenetic tree by maximum likelihood of ORs built from amino acid sequences of *M. helenor* “MhelOR” (red), *M. achilles* “MachOR” (green), *M. deidamia* “MdeiOR” (blue), and *S. littoralis* “SlitOR.” The latest were colored following their chemical class (magenta: aromatics; cyan: terpenes; orange: aliphatics; black: unclassified) as defined in [34].
