# Supplementary material for: Genome assembly of 3 Amazonian Morpho butterfly species reveals Z-chromosome rearrangements between closely related species living in sympatry
Source: Gigascience. 2023 May 22;12:giad033. doi: 10.1093/gigascience/giad033 (PMC10202424; doi:10.1093/gigascience/giad033)
Supplement: giad033_GIGA-D-22-00294_Revision_1 [file giad033_giga-d-22-00294_revision_1.pdf]

GigaScience

Genome assembly of three Amazonian Morpho butterfly species reveals Z-chromosome rearrangements between closely-related species living in sympatry

--Manuscript Draft--

Manuscript Number:	GIGA-D-22-00294R1
Full Title:	Genome assembly of three Amazonian Morpho butterfly species reveals Z-chromosome rearrangements between closely-related species living in sympatry
Article Type:	Research
Funding Information:	Centre National de la Recherche Scientifique (MITI program) — Ms Violaine Llaurens<br>Muséum National d'Histoire Naturelle (ATM program) — Ms Violaine Llaurens<br>Agence Nationale de la Recherche (ANR-T-ERC-OUTOFTHEBLUE) — Ms Violaine Llaurens
Abstract:	The genomic processes enabling speciation and the coexistence of species in sympatry are still largely unknown. Here we describe the whole genome sequencing and assembly of three closely-related species fromthe butterfly genus Morpho : Morpho achilles (Linnaeus, 1758), M. helenor (Cramer, 1776) and M. deidamia (Hübner, 1819). These large blue butterflies are emblematic species of the Amazonian rainforest. They live in sympatry in a wide range of their geographical distribution and display parallel diversification of dorsal wing colour pattern, suggesting local mimicry. By sequencing, assembling and annotating their genomes, we aim at uncovering pre-zygotic barriers preventing gene flow between these sympatric species. We found a genome size of 480 Mb for the three species and a chromosomal number ranging from 2n = 54 for M. deidamia to 2n = 56 for M. achilles and M. helenor. We also detected inversions on the sex chromosome Z that were differentially fixed between species, suggesting that chromosomal rearrangements may contribute to their reproductive isolation. The annotation of their genomes allowed us to recover in each species at least 12,000 protein-coding genes and to discover duplications of genes potentially involved in pre-zygotic isolation like genes controlling colour discrimination (L-opsin). Altogether, the assembly and the annotation of these three new reference genomes open new research avenues into the genomic architecture of speciation and reinforcement in sympatry, establishing Morpho butterflies as a new eco-evolutionary model.
Corresponding Author:	Héloïse Bastide<br>Université Paris-Saclay: Universite Paris-Saclay<br>Gif-sur-Yvette, FRANCE
Corresponding Author Secondary Information:	
Corresponding Author's Institution:	Université Paris-Saclay: Universite Paris-Saclay
Corresponding Author's Secondary Institution:	
First Author:	Héloïse Bastide
First Author Secondary Information:	
Order of Authors:	Héloïse Bastide<br>Manuela López-Villavicencio<br>David Ogereau<br>Joanna Lledo<br>Anne-Marie Dutrillaux<br>Vincent Debat<br>Violaine Llaurens

Powered by Editorial Manager® and ProduXion Manager® from Aries Systems Corporation

Order of Authors Secondary Information:	
Response to Reviewers:	Reviewer reports:<br>Reviewer #1: Summary<br><br>This manuscript presents three, high-quality genomes for three charismatic butterfly species. These represent the first genomes for any Morpho species and serve as an important resource for the community. The analyses conducted on these genomes demonstrate their value and the use of these species as a model for exploring evolutionary processes such as speciation and exploring the role of chromosome change in such processes. The methods are appropriate and have been carried out in a considered way.<br><br>In the most part, the manuscript clearly describes how the data was obtained and analysed. In particular, the descriptions of how the genomes were sequenced, assembled and annotated are very thorough. The clarity of the text could be improved in some places. However, this is very minor and overall it reads well and clearly describes how analyses were conducted and presents an interesting discussion of the results.<br><br>We thank the referees for their time and for the comments made to our manuscript. Our answers are included in the text.<br><br>The following minor comments apply to the methods:<br><br>The description of how the karyotype analyses were conducted was more limited compared to the genomic descriptions. While the brevity is understandable as the authors used a protocol similar to what was described in a previous study, additional details such as which stain was used is required as two were used in the cited study (McClure et al. 2017).<br><br>The Giemsa method was used to stain the Morpho chromosomes and this precision is now added to the manuscript (l. 113-114).<br><br>I could not find any project records when I searched for project number PRJEB56642 on ENA. Please could the data be made publicly available before the manuscript is accepted.<br><br>The data will be released as soon as the manuscript is accepted.<br><br>Genome annotations are not currently included in the supporting information, please could they be added as part of the genome assembly submissions, or at the very least as a citable data object on zenodo.org etc as they serve as a valuable resource to other researchers.<br><br>The transcriptomic and proteic sequences of the putative proteins predicted by Maker along with the gff files will be available upon request. This information was added in the manuscript (l. 636-637 and l. 641-642).<br><br>In the assembly section the authors state that 'Purge_dups v1.2.5 setting the cutoffs manually'. It would be helpful to state what values were used as cutoffs for the final analysis for reproducibility.<br><br>Done (l. 170-172).<br><br>State whether BUSCOs was run in augustus or metaeuk mode (metaeuk is the default in BUSCO v5).<br><br>Done (l. 163).<br><br>"We removed short scaffolds, short alignments and low identity alignments with the R script proposed in [34]" - did you use the same thresholds as in ref [34]? If so explicitly state this. In Ref [34] they state that "The alignment file was filtered to retain only those

Powered by Editorial Manager® and ProduXion Manager® from Aries Systems Corporation

aligned sequences that were longer than 200 bp and had less than 90% identity between the two genomes in contigs that were at least 1 Mb long."

Done (l. 249-254).

Filtering of scaffolds and visualisation of synteny was achieved using two Rscripts. Currently, the references to the papers where the Rscripts were originally used are given but getting from these to the actual scripts takes some effort.
For the first (filtering, ref[34]) I could find the relevant script: https://github.com/bioinfowheat/Polygonia_calbum_genomics/blob/7c75aac624157faa3ab229e3fc1e0e315302194d/synteny/circlePlot_nucmerOutput.R . I think it would be helpful to include this location in the manuscript for greater visibility.
I could not find the script referenced in ref[36] for visualising synteny. If the link to this script could be found, that would also be beneficial to be included.

Done. The script used in this section is now included in the text (l. 246-254).

Minor comments on rest of text:

Given that the close relatedness of these three species is a key feature of this study, and is what makes this such an interesting system, I think it would be helpful to mention the estimated divergence time between the species pairs from previous studies (M. helenor vs M. achilles: 6.8 mya; M. helenor vs M. deidamia: 11.9 mya) (Penz et al. 2012)

We agree with this relevant suggestion, and we added the divergence times estimated in the latest publication on the diversification of Morpho species (Chazot et al. 2021) that slightly differ from the numbers reported in Penz et al. 2012 (l. 62-67).

While it is mentioned in the discussion that two of the three species have previously been karyotypically described, this is not mentioned anywhere else in the text. It would be good to acknowledge this previous work also in the results section briefly to state that the results here are consistent with previous work. For example here, "First, we characterized the caryotypes of the three studied species (see sup. figure 2 to visualize the chromosomes)."

This is again a highly relevant comment. We now state that the modal number of chromosomes in the Morphinae is n=28 in the article of Brown et al. 2007, and that n=28 chromosomes were found in M. helenor and M. achilles in the same study, consistent with our own findings (l. 302-306).

"Chromosomal rearrangements are likely to play a major role in both adaptation and speciation processes [1]"

The reference used only references inversions, not other sources of rearrangements. Suggest using either a more general reference such as Feulner & De Kayne, 2021 or adding extra references for other types of rearrangements. May also be worth explicitly listing types of rearrangements such as duplications given these are a key aspect of the manuscript.

This reference has been added (l. 70).

Consider combining the last two paragraphs of the introduction which are short into one paragraph, and moving the sentences around to allow the text to flow better. For example, "By obtaining karyotype data and generating whole genome sequences for three sympatric species of Morpho buttterflies, we.."

We tried to combine these two short paragraphs in a single one as suggested by the referee (l. 60-78).

"Males from the species M. helenor, M. achilles and M. deidamia were caught with a handnet at the Patawa waterfall, located in the Kaw mountain area of French Guiana"

Powered by Editorial Manager® and ProduXion Manager® from Aries Systems Corporation

Explicitly state the number per species in brackets after each species.

This sampling was performed during a mark-recapture experiments carried out in July 2019 and July 2021, where we caught a large number of Morphos (n=395) but kept only few specimens for sequencing and karyotyping. We added the number of specimens per species used for DNA extraction, sequencing and karyotyping in brackets in the manuscript (l. 92-93 and l. 101-102).

"To assess variations in chromosome-scale synteny, we compared the assemblies of each Morpho to the assembly of M. jurtina, the closest relative of Morpho for which a high quality chromosome level assembly (based in N50 values and Busco score, accession ID GCF_905333055.1) is available [27]."

It would be helpful to state the chromosome number of M. jurtina (n=29) in this context.

Done (l. 224).

The authors state the number of M. achilles individuals which were used for karyotype analyses but do not state the number for M. helenor or M. achilles.
We used n=3 specimens of M. helenor, n=2 specimens of M. deidamia and n=4 specimens of M. achilles for karyotypic analyses, the exact numbers are now provided in the manuscript (l. 92-93).

"GenomeScope analyses suggested relatively similar genome sizes (between 470 and 489 Mb) and very high levels of heterozygosity for the three species 1."

Should this say Table 1? If so, why are the genome sizes in the table different to the text (335-380 Mb in Table 1)? Values 470-489 Mb correspond to final assemblies (post-purging for H. helene and H. achilles) based in Sup Tab 1.

I would suggest that Table 1 is altered to show the statistics for the final assemblies per species (haploid genome size, number of scaffolds, N50, heterozygosity, BUSCO genome complete/fragmented %). It could also include the number of predicted genes and BUSCO proteome complete/fragmented %.

We have now fused the genomescope heterozygosity results and statistics table in one single table following the suggestions made by the referee (Table 1).
We have also added the results of BUSCO run in the protein mode to get the BUSCO score of the proteome. Nevertheless, we placed all BUSCO results as figures in the supplementary material because when added to a general table, the size of the table was difficult to read (supplementary figures 4 and 5).

"The use of purge_dups significantly reduced thenumber ofduplicates"

I'd say "strongly" rather than "significantly" as that can imply that statistics have been carried out.

Corrected (l. 298).

"In order to assess if the annotations were complete, we estimated in each species the percentage of proteins with a Pfam domain … 65,50% in M. achilles to 71,32% in M. helenor …showing that the annotations were of good quality."

While I agree that this shows that the predicted genes are of good quality, it doesn't necessarily show how complete the annotation is. A quick extra piece of information would be to run BUSCO in proteome mode on the annotation and compare the resulting completeness result to that of the genome.

Done. These results have been added to the supplementary material (supplementary figure 5) and a sentence has been added to the text (l. 332-336).

"The phylogenetic relationships between the copies in the three species reveal that the duplications observed in the three Morpho species probably occurred before their

speciation"

I agree with the authors that this is by far the most likely explanation for the data. It might be worth saying however that the alternative explanation (which is far less likely!) is that the three species share ancient haplotypic variation. It might be helpful to show Fig 2 as a tree rather than a cladogram, thus including branch lengths to evaluate this but I don't think this is necessary.

The tree indeed shows that the level of resemblance of the opsin copy sequences among the three species was correlated with their level of genomic divergence, suggesting that theses copies were ancestral to the diversification of these three species and then evolved independently within each lineage. Nevertheless, we agree that we cannot precisely date the duplication events, we are currently aiming at detecting these copies throughout the genus Morpho to infer their putative origin (within the genus Morpho or ancestral to the diversification of this genus). We thus added a comment on the lack of precision we currently have on the evolutionary origin of these two duplications (l. 361-363).

"The dot-plots from the paired comparisons between the three Morpho using D-GENIES showed a very high similarity between genomes (see sup. figure 7)."

The use of D-GENIES is mentioned in the methods so is not needed again in the results. Instead of stating the tool, it might be more helpful to the reader to describe the analysis e.g. "pairwise comparisons of synteny…"

The sentence has been modified (l. 445-447).

"In M. deidamia, the Hifiasm assembly showed a single scaffold ptg000028l containing chromosomes NC_060051.1 and NC_060052.1 from M. jurtina."

I believe this should be ptg000008l rather than ptg000028l. It also looks like ptg0000161l is syntenic to two chromosomes in M. jurtina (NC_060054.1 and NC_060055.1). I

The referee is right, we have changed the sentences (l. 413-423).

Based on the synteny plots in Fig 3, it seems that after filtering, H. helenor and H. deidomia have scaffolds with synteny to 27 of the 29 chromosomes of M. jurtina. In contrast, H. achilles has scaffolds with synteny to all 29 chromosomes of M. jurtina. Presumably, the sequences corresponding to the two missing homologous chromosomes in H. helenor and H. deidomia are in the filtered out scaffolds. It might be good to check this and report which scaffold(s) do map to these two M. jurtina chromosomes and which filter caused them to be dropped from the analysis as other users of this data may be interested in these. It is also a good sanity check that no data has been lost.

We think the difference in the chromosome and contig number between M. helenor and M. deidamia (27 contigs each) vs M. jurtina (29 chromosome pairs) is not due to the filtering of sequences in Morpho.
In the case of M. helenor, the biggest contig in the assembly (ptg000028l with a size of 42 Mb), appears syntenic in the plot to two chromosomes of M. jurtina (chromosomes 2 and 6 with sizes of 17.19 Mb and 16.2 Mb respectively). As the number of contigs recovered by the synteny plot in M. helenor is less than the number of chromosomes observed in the karyotype of this species and as the size of contig ptg000028l is twice the size of every other contig in any Morpho species we analyzed, we believe this very large contig was produced by an overassembly of two chromosomes into a single contig by hifiasm.
In the case of M. deidamia, Maniola's chromosomes 24 and 25 (with sizes 10.05 and 9.43 Mb respectively) appear syntenic to a single contig in deidamia (contig ptg000008l with a size of 20.29 Mb). Nevertheless, synteny plots and karyotype of this species gave the same number of contigs/chromosomes (27). We then suggest that in this

case, the chromosome corresponding to the contig ptg000008l in M. deidamia could have been formed by fusion of two chromosomes of M. jurtina.
We have tried to clarify this in the text (l. 413-423).

In Fig 3, could the scaffolds that are described in the text (e.g. ptg000008l) be annotated in some way to allow the reader to more easily locate them, for example by making the text label red?

Done. We have now labeled Z chromosomes in red and single chromosomes in Morpho that are not assigned to a single chromosome in M. jurtina in blue.

The Morpho species labels in Fig 3 and Fig4 are red, green and blue which may prove difficult for colour blind readers. It may be helpful to change one of these colours.

Done. We have changed the green color used in M. helenor.

Genomes on a Tree (GoaT) (https://goat.genomehubs.org/) predicts a genome size of 474 Mb for Morpho species so the values obtained here are indeed very close to the estimate. This may be worth adding but not essential!

Done. This was added in the discussion (l. 468-470).

"The karyotype and assembly analyses suggest some differences in chromosome number between the three sympatric Morpho species studied here, particularly between M. deidamia (27 chromosome pairs) and M. achilles (28 or 29 chromosome pairs)."

As in the results, M. achilles is described as having n=28 in all four specimens studied, I am not sure why "28 or 29" is stated here.

This sentence has been corrected (l. 523-526).

"In two ascidians species of the genus Ciona and in insects like Drosophila inversions may promote speciation by reduction of the fitness or by causing sterility of heterozygotes."

Reference(s) missing.

The references have been added (l. 550-552).

"Figure 4. Rearrangement (SyRI) plot of the paired comparisons for the Z scaffold between the three Morpho species. Upper figure: M. helenor andM. deidamia; middle: M. deidamia andM. achilles; lower: M. helenor andM. deidamia. SyRI results were plotted using plotsr"

"SYRI results were plotted using plotter" should be in methods rather than the figure legend.

This was removed from the figure caption and highlighted in the methods (l. 263-264).

Lower text should say 'M. helenor and M. achilles' rather than 'M. helenor and M. deidamia'.

Corrected.

'Synteny and rearrangement plot' is more descriptive than 'rearrangement plot'.

The sentence has been modified.

"Data show that across the Passeriformes, the Z chromosome has accumulated more inversions than any other autosome and that the inversion fixation rate on the Z chromosome is 1.4 times greater than the average autosome."

Explicitly describe the type of data e.g. 'cytological and genomic data..'.

This has been added (l. 559).

"Further population analyses are needed to answer this question and to enlighten what evolutionary forces could be acting to maintain them."
I suggest removing 'Further' as that suggests population analyses have been presented here.

Done (l. 589).

"For instance, the three copies of LW opsins found in the Papilio genus (fig.)"
I think this should read as "Fig 2"?

Corrected (l. 595).

"Interestingly, a blast of the putative proteic sequences of each Morpho species against those of M. jurtina allowed us to uncover different copy numbers of the gene bric - a - brac, which play a significant role in differences of UV iridescence between males of two incipient species of sulphur butterflies [60]."

Similar to comment 13, While a blast search is what was carried out, it would be more helpful to the reader to describe the analysis rather than the tool used e.g. "a homology search"

Done (l. 605).

Scale bar is missing from Sup Fig 2.

The scale bar was added to Sup Fig 2.

Reviewer #2: The authors report three high quality genomes of three closely related species of Morpho butterflies. While the genomes appear to be normal butterfly genomes, with no surprising features, the species involved are iconic and the whole genus does not have a genome available previously. For the research community, one might say finally a Morpho has been genomed! I did find it interesting that three species seem to have lower numbers of genes for the olfactory receptors, these butterflies are well known to be easy to catch in butterfly bait traps baited with rotting fruit and other smelly types of bait. Perhaps the remaining ORs are those that help them find rotting fruit in the forests?

We added a phylogenetic analysis of the OR sequences uncovered in the three Morpho species along with those of the moth Spodoptera littoralis, in which a number of ORs have been previously deorphanized. Our results show that the remaining ORs were not clustered around one function in particular. We would thus need further functional characterization of the remaining ORs in the Morpho species to fully answer this question. We modified the results to include this information, added a supplementary figure with the phylogenetic tree (Supplementary Figure 9) and edited the methods accordingly (l. 365-390 and l. 207-220 respectively).

Another interesting point made in the manuscript is that the Z chromosome might have something to do with keeping the species separate. The authors did not cite another paper suggesting this for Junonia butterflies (DOI: 10.1111/syen.12428), and although the quality of data for that
paper is much lower than for this manuscript, the authors of the Junonia paper do extensively discuss the role of the Z chromosome in maintaining species boundaries in closely related species. Perhaps something to include in the discussion?

We added the reference in the discussion (l. 622-626).

Powered by Editorial Manager® and ProduXion Manager® from Aries Systems Corporation

	Otherwise the paper is very well written, and the availability of these new genomes to the research community is highly exciting. I have only one minor point to make, the GPS coordinates place the collection site in the middle of the Atlantic off the coast of West Africa, i.e. the longitude should -52.15832.<br><br>Corrected (l. 83-84).
Additional Information:	
Question	Response
Are you submitting this manuscript to a special series or article collection?	No
**Experimental design and statistics**<br><br>Full details of the experimental design and statistical methods used should be given in the Methods section, as detailed in our Minimum Standards Reporting Checklist. Information essential to interpreting the data presented should be made available in the figure legends.<br><br>Have you included all the information requested in your manuscript?	Yes
**Resources**<br><br>A description of all resources used, including antibodies, cell lines, animals and software tools, with enough information to allow them to be uniquely identified, should be included in the Methods section. Authors are strongly encouraged to cite Research Resource Identifiers (RRIDs) for antibodies, model organisms and tools, where possible.<br><br>Have you included the information requested as detailed in our Minimum Standards Reporting Checklist?	Yes
**Availability of data and materials**<br><br>All datasets and code on which the conclusions of the paper rely must be either included in your submission or deposited in publicly available repositories (where available and ethically	Yes

appropriate), referencing such data using a unique identifier in the references and in the “Availability of Data and Materials” section of your manuscript.<br><br>Have you have met the above requirement as detailed in our Minimum Standards Reporting Checklist?	

Powered by Editorial Manager® and ProduXion Manager® from Aries Systems Corporation

Manuscript

Click here to access/download;Manuscript;Manuscript.pdf

GigaScience, 2022, 1–10

doi: xx.xxxx/xxxx
Manuscript in Preparation
Paper

PAPER

Genome assembly of three Amazonian *Morpho* butterfly species reveals Z-chromosome rearrangements between closely-related species living in sympatry

Héloïse Bastide[1,*,†], Manuela López-Villavicencio[2,†], David Ogereau[1], Joanna Lledo[3], Anne-Marie Dutrillaux[2], Vincent Debat[2] and Violaine Llaurens[2]

[1]IDEEV, Bât. 680,12, route 128 91190 Gif Sur Yvette, France and [2]Institut de Systématique, Evolution et Biodiversité (UMR 7205 CNRS/MNHN/SU/EPHE/UA), Muséum National d'Histoire Naturelle - CP50, 45 rue Buffon 75005 PARIS, France and [3]GeT-PlaGe, Bât G2, INRAe, 24 chemin de borde rouge - Auzeville, CS 52627, 31326 CASTANET-TOLOSAN Cedex, France

[*]Corresponding author
[†]Contributed equally.

Abstract

The genomic processes enabling speciation and the coexistence of species in sympatry are still largely unknown. Here we describe the whole genome sequencing and assembly of three closely-related species from the butterfly genus *Morpho* : *Morpho achilles* (Linnaeus, 1758), *M. helenor* (Cramer, 1776) and *M. deidamia* (Hübner, 1819). These large blue butterflies are emblematic species of the Amazonian rainforest. They live in sympatry in a wide range of their geographical distribution and display parallel diversification of dorsal wing colour pattern, suggesting local mimicry. By sequencing, assembling and annotating their genomes, we aim at uncovering pre-zygotic barriers preventing gene flow between these sympatric species. We found a genome size of 480 Mb for the three species and a chromosomal number ranging from $2n = 54$ for *M. deidamia* to $2n = 56$ for *M. achilles* and *M. helenor*. We also detected inversions on the sex chromosome Z that were differentially fixed between species, suggesting that chromosomal rearrangements may contribute to their reproductive isolation. The annotation of their genomes allowed us to recover in each species at least 12,000 protein-coding genes and to discover duplications of genes potentially involved in pre-zygotic isolation like genes controlling colour discrimination (*L-opsin*). Altogether, the assembly and the annotation of these three new reference genomes open new research avenues into the genomic architecture of speciation and reinforcement in sympatry, establishing *Morpho* butterflies as a new eco-evolutionary model.

Key words: Sympatric speciation; reinforcement; mimicry; structural variant; inversions; gene duplication; wing colour pattern; evolutionary convergence: genomic divergence; karyotype.

Introduction

Chromosomal rearrangements are likely to play a major role in both adaptation and speciation processes (1, 2). Inversions, for instance, can favour the emergence of adaptive syndromes by locking together co-adapted allelic variations [3]. Chromosomal rearrangements have also been suggested to contribute to reproductive isolation between species by promoting divergent adaptation or by bringing together genetic incompatibilities [4]. Nevertheless, the role of structural variants in these evolutionary processes is still largely unknown. Recently-developed sequencing and assembly methods now provide access to complete genomes, therefore opening the investigation of structural variation within and among species (see [5] for a review).

Compiled on: February 13, 2023.
Draft manuscript prepared by the author.

Key Points

- This is the first point
- This is the second point
- One last point.

Here, we focus on emblematic species of the Amazonian rainforest, the blue *Morpho*. We describe the whole genome sequences of three closely-related *Morpho* species living in sympatry for a large range of their geographical distribution (Fig. 1) : *M. helenor*, *M. achilles* and *M. deidamia* [6], thereby developing relevant resources to study the evolution of barriers to gene flow in sympatry. In Lepidoptera, specialization towards host-plant has been shown to be a major factor affecting species diversification [7]. Such ecological specialization may favour speciation and co-existence in sympatry, and may stem from the evolution of gustatory receptors enabling plant recognition by females [8].

The evolution of visual [9] and olfactory signals [10] between species may also limit gene flow between sympatric species of Lepidoptera. In the three *Morpho* species studied here, both males and females display conspicuous iridescent blue colour patterns on the dorsal side of their wings, combined with cryptic brownish colour on the ventral side [11]. Such a combination of dorso-ventral pattern, associated with a fast and erratic flight, is thought to contribute to the high escape abilities from predators of these butterflies, promoting colour pattern convergence between sympatric species (*i.e.* escape mimicry, [12]). Parallel geographic variation of dorsal wing colour pattern has indeed been detected in the three *Morpho* species studied here, suggesting local convergence promoted by predators behaviour [13]. Given the key role of colour pattern in both sexual selection and species recognition in diurnal butterflies, such a resemblance is thought to enhance reproductive interference between sympatric species [14]. Behavioural experiments carried out in the wild revealed that males from the three mimetic *Morpho* species are indeed attracted by both intra and interspecific wing patterns [15]. Despite this heterospecific attraction of males at long distances, RAD-sequencing markers revealed a highly limited gene flow between these three sympatric species [15]. This might be due to the differences in the timing of daily activities observed between these sympatric species limiting heterospecific encountering [15]. This divergence in daily phenology may contribute to the initiation of speciation or to the reinforcement of pre-zygotic barriers to heterospecific matings.

Genetic incompatibilities may also contribute to speciation and reinforcement processes by generating post-zygotic barriers. For instance, variation in chromosome numbers has been shown to correlate with speciation rate in Lepidoptera [16]. Similarly, chromosomal inversions may fuel the speciation process: by capturing genetic variations, inversions may lead to increased genetic divergence between species. Such divergence may lead to maladaption in hybrids and further limit gene flow between species living in sympatry.

By relying on both karyotype data and PacBio-Hifi sequencing, we generated *de novo* genome assemblies for three sympatric species of *Morpho* butterflies. The divergence between the two sister species *M. helenor* and *M. achilles* was estimated to occur about 3.91 My ago, while the divergence between these two sister species and *M. deidamia* was estimated to circa 16.68 My ago [17], enabling to compare the genome divergence in sympatry at different time scales. We then investigated the structural variants and variation in genes potentially contributing to pre-zygotic isolation among these species. We aim to shed light on the genomic processes involved in sympatric speciation and reinforcement as well as detecting chromosomal rearrangements. We also provide their mitochondrial genomes, study their transposable element (TE) contents and annotate the genomes. These genomic resources will open new research avenues into the understanding of adaptive processes, such as convergence evolution of colour pattern or divergence in visual systems, as well as speciation and co-existence of sister-species in sympatry, establishing *Morpho* butterflies as a new eco-evolutionary model.

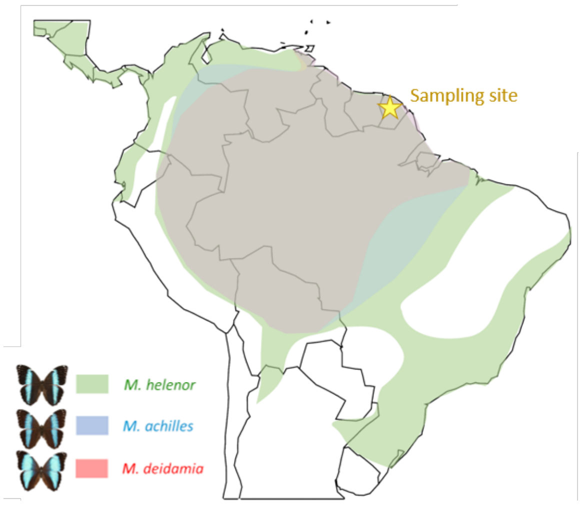

Figure 1. Geographical distribution of the three neotropical species *M. helenor* (green areas), *M. achilles* (blue areas) and *M. deidamia* (red areas). *M. helenor* has the widest distribution, from central America to Southern Brazil, while *M. achilles* and *M. deidamia* are restricted to the Amazonian basin. The three species are in sympatry throughout the Amazonian rainforest, including French Guiana (marked with the yellow star) where the samples studied here were collected.

Material and methods

Butterfly sampling

Males from the species *M. helenor* (n = 1), *M. achilles* (n = 4) and *M. deidamia* (n = 2) were caught with a handnet at the Patawa waterfall, located in the Kaw mountain area of French Guiana (GPS location: 4.54322; -52.15832) to perform DNA extractions. In these species, males typically patrol in river beds and are easy to catch, while females are more rarely encountered. We therefore focused on males only. Because in butterflies sex is controlled by a ZW sex chromosome system (females being the heterogametic sex), we were thus able to access the Z sex chromosome but not the W chromosome.

Karyotype study

Cytogenetic techniques were applied to wild caught males (*M. helenor* (n = 3), *M. achilles* (n = 4) and *M. deidamia* (n = 2)) that were collected at the above-mentioned location in 2019. Their testicles were dissected and processed shortly after capture following the

protocol described in [18]. The obtained cell suspension was conserved in fixative at about 4°C. The cell spreading and staining were then performed as described in [18]. The chromosome staining relied on the Giemsa method.

DNA extractions and genome sequencing

Live butterflies (*M. helenor* (n = 1), *M. achilles* (n = 4) and *M. deidamia* (n = 2)) captured in 2021 at the same site in French Guiana were killed in the lab and their body immediately placed in liquid nitrogen. The DNA extraction was carried out the following day using the Qiagen Genomic-tip 100/G kit and following supplier instructions. The extracted DNA of a single male from each species was used (see Supplementary Fig. 1 for pictures of the wings of the sequenced specimens). Library preparation and sequencing were performed at GeT-PlaGe core facility (INRAe Toulouse) according to the manufacturer's instructions "Procedure and Checklist Preparing HiFi SMRTbell Libraries using SMRTbell Express Template Prep Kit 2.0". At each step, DNA was quantified using the Qubit dsDNA HS Assay Kit (Life Technologies). DNA purity was tested using the nanodrop (Thermofisher) and size distribution and degradation assessed using the Femto pulse Genomic DNA 165 kb Kit (Agilent). Purification steps were performed using AMPure PB beads (Pacific Biosciences). 15µg of DNA was purified then sheared at 15kb (speed 31 and 32) with the Megaruptor3 system (Diagenode). Using SMRTbell Express Template prep kit 2.0, a Single strand overhangs removal, a DNA and END damage repair step were performed on 10µg of sample. Blunt hairpin adapters were then ligated to the library, which was treated with an exonuclease cocktail to digest unligated DNA fragments. A size selection step using a 10kb cutoff was performed on the BluePippin Size Selection system (Sage Science) with the "0.75 percent DF Marker S1 6-10 kb vs3 Improved Recovery" protocol. Using Binding kit 2.2 and sequencing kit 2.0, the primer V5 annealed and polymerase 2.2 bounded library was sequenced by diffusion loading onto 1 SMRTcells per sample on SequelII instrument at 80 pM with a 2 hours pre-extension and a 30 hours movie.

K-mer analysis, genome size and heterozygosity estimation

We used Jellyfish (v.2.3.0) [19] to perform a *k*-mer analysis on each PacBio dataset with a *k*-mer size of 21. For each dataset *k*-mers were counted and aggregated (jellyfish count option) and histograms were generated using the "-histo" command. The resulting histograms allowed the estimation of genome length and heterozygosity with GenomeScope version 2.0 [20] using the web application.

Nuclear and mitochondrial genome assembly

For the assembly of the nuclear genomes, we compared three long-read assembly tools: IPA-Improved Phased Assembler (v1.0.3-0) (https://github.com/PacificBiosciences/pbipa), Flye (v2.9) [21] and Hifiasm (v0.16.1 with the option -l3 to purge all types of haplotigs in the most aggressive way) [22]. For each assembler, we estimated basic assembly statistics such as scaffold count, contig count and N50 using the "stats.sh" program from the BBMap v38.93 package [23]. The completeness of each assembly was assessed using BUSCO v5.2.2 and MetaEuk for gene prediction against the *lepidoptera_odb*10 database [24]. We retained the Hifiasm assembly because it had the highest BUSCO score, the highest contiguity (N50) and longest contig. Despite the high level of purging performed by Hifiasm, the species (*M. helenor* and *M. achilles* respectively) retained a high level of duplicates in the BUSCO score. To remove false haplotypic duplications in these two species, we used Purge_dups v1.2.5 setting the cutoffs manually (with calcuts -l 5 -m 33 -u 135 for *M. helenor* and calcuts -l 10 -m 45 -u 145 for *M. achilles*) [25]. The completeness of the purged genomes was then reassessed using BUSCO.

The mitochondrial genome of each species was assembled and circularized using Rebaler (https://github.com/rrwick/Rebaler) directly from the PacBio Hifi reads and using the mitochondrial genome of the closely related species *Pararge aegeria* as a reference.

Annotation of repetitive regions

The annotation of repetitive regions in the three species was performed following two main steps. First, we used RepeatModeler v2.0.2a [26] with the option -s (slow search) and -a (to get a .align output file) to create *de novo* libraries of repetitive elements for each species. The library was then used to hardmask the corresponding genome assembly using RepeatMasker 4.1.2.p1 [26] . A summary of the repeated elements was generated with the script 'buildSummary.pl' included in RepeatMasker.

Genome annotation

Each of the three genomes was independently annotated using Maker v2.31.10 [27], following the protocol given in [28]. In short, Maker is usually run several times successively and uses the gene models generated in one round to train *ab initio* gene-predictors and improve the initial gene models in the next round (see below). We used the above-mentioned hardmasked genomes and carried out their annotation using the proteomes of three closely-related species, namely *Pararge aegeria* [29], *Maniola hyperantus* [30] and *Bicyclus anynana* [31]. For each species, the output files were merged into a gff3 file that was then used to generate the necessary files to train SNAP (version 2006-07-28), an *ab initio* gene finding program [32]. A second run of Maker with the above-mentioned gff3 file and the .hmm file provided by SNAP resulted in a second gff3 file that was used to train SNAP a second time. A third round of Maker with the second gff3 and .hmm files was followed by the training of Augustus (3.3.3), another gene prediction tool [33], with the third gff3 file. A final round of Maker with the third gff3 file and the files generated by Augustus led to the fourth and last gff3 file, containing all the genome features for each species.

Protein-Protein BLAST 2.9.0+ (-evalue 1e-6 -max_hsps 1 -max_target_seqs 1) was then used to assess putative protein functions in each *Morpho* species by comparing the protein sequences given by Maker to the protein sequences from the annotated genomes of *Maniola jurtina* [29], *P. aegeria* [29], *B. anynana* [31] and *S. littoralis* specifically for the detection of OR sequences [34]. We used BUSCO to assess the completeness of the proteome with the protein mode and the *lepidoptera_odb*10 database on the annotated gene set produced by MAKER [24].

Phylogenetic analysis

To specifically compare the exon sequences of the opsins detected in the *Morpho* genomes to the opsins described in other Lepidoptera, we retrieved the coding sequences of opsins from NCBI and used the software Mega v.11 [35] to build a maximum likelihood tree and compute the associated bootstrap values.

Regarding the OR repertoire in the three *Morpho* species, we curated the sequences obtained by blast comparison of the Maker-annotated genes on the reference genome of *S. littoralis*, as a number of sequences showed incorrect lengths (<300 or >500 amino acids). We used exonerate version 2.4.0 [36] with the options -maxintron 2000 independently in each *Morpho* species. The exonerate alignment files and the assemblies were used with InsectOR (`http://caps.ncbs.res.in/gws_ors/`), a website specifically designed to help predict OR genes from insect genomes, with the op-

tion HMMSEARCH against 7tm_6 [37], [38]. The sequences uncovered with insectOR for each *Morpho* species were aligned with the ORs of *S. littoralis* using MAFFT [39] and we generated a maximum-likelihood phylogenetic tree with IQ-TREE version 2.2.0 [40] with the options -bb 1000 and -nt AUTO.

Synteny and rearrangement detection

To assess variation in chromosome-scale synteny, we compared the assemblies of each *Morpho* to the assembly of *M. jurtina*, the closest relative of *Morpho* with a karyotype of 29 chromosomes and for which a high quality chromosome-level assembly (based on N50 values and Busco score, accession ID GCF_905333055.1) is available [29]. We used MUMmer 3.23 [41] to align the masked assembled genomes of *M. helenor*, *M. achilles* and *M. deidamia* to the *M. jurtina* genome. The output produced by MUMmer is an ASCII delta file that was then filtered and parsed using the utility programs delta-filter and show-coords from MUMmer. Synteny was visualized with the MUMmer results in R with the packages circlize v 0.4.12 [42] and Paletteer (`https://github.com/EmilHvitfeldt/paletteer`) using the Rscript from [43] described here: `https://github.com/bioinfowheat/Polygonia_calbum_genomics/blob/7c75aac624157faa3ab229e3fc1e0e315302194d/synteny/circlePlot_nucmerOutput.R`, removing short contigs, short alignments (less than 200bp) and low identity alignments (less than 90% identity).

In order to detect potential genome rearrangements between *Morpho* and closely-related species, we estimated the whole-genome collinearity between the *Morpho* assemblies and five closely-related Nymphalidae species whose genomes exhibit a good-quality assemblies in the NCBI genome database: *M. jurtina* (GCA_905333055.1), *P. aegeria* (GCA_905333055.1), *Erebia ligea* (GCA_923060345.2), *Melanargia galathea* (GCA_920104075.1) and *Lasiommata megera* (GCA_928268935.1) using D-GENIES [44]. Paired alignments between a *Morpho* species and one Nymphalidae species were performed using the minimap2 aligner [45] in D-GENIES, treating each *Morpho* species genome as the query and the Nymphalidae species genome as the target reference. We also used D-GENIES to pair-compare the genomes of the three *Morpho* species. As D-GENIES revealed differences between *Morpho* species in the contig corresponding to the Z chromosome (see results), we used SyRI [46] to study in detail the rearrangements in the sequences of this contig between the three species. We generated paired alignments of the Z contig with minimap2 and ran SyRI with the option -c on .sam files. SyRI requires that the two compared genomes represent the same strand and in the case of *M. achilles*, the orientation of the sequence produced by HiFiasm was the complementary to the sequences of *M. helenor* and *M. deidamia*. We then reverse-complemented this sequence in order to make the alignments. All the genomic structures predicted by SyRI were plotted using plotsr [47].

Results

Comparing karyotypes between species

First, we characterized the karyotypes of the three studied species (see Supplementary Fig. 2 to visualize the chromosomes). In *M. helenor*, the detected number of diploid chromosomes ranged from 54 to 56 in the different replicates of mitoses, with a discreet mode at $2n = 56$. This variation is probably due to technical difficulties. The presence of $n = 28$ bivalents in metaphase confirmed the diploid number of $2n = 56$ chromosomes. In *M. achilles*, four specimens had the same modal chromosome counts: mitoses: $2n = 56$ chromosomes; pachynema: $n = 28$ bivalents; Metaphases I: $n = 28$ bivalents; Metaphases II: $n = 28$ chromosomes with 2 chromatids.

Table 1. Genome heterozygosity estimated with GenomeScope and Genome statistics for the assemblies of three *Morpho* species using different computational methods. Assemblies were purged using purge_dups. Statistics were obtained with BBMap. The assembly produced with Hifiasm for the individual *M. deidamia* was not purged with purge_dups as BUSCO results on the preliminary assembly revealed a very low duplicate content.

	M. helenor	*M. achilles*	*M. deidamia*
Heterozygosity (%)	3.35	2.78	1.68
Assembly method			
Hifiasm			
Total contigs	143	32	58
Genome size	470.254 Mb	478.514 Mb	489.914 Mb
N50	12 Mb	12 Mb	13 Mb
IPA			
Total contigs	128	56	47
Genome size	473.620 Mb	493.177 Mb	481.177 Mb
N50	17 Mb	14 Mb	13 Mb
Flye			
Total contigs	134	114	291
Genome size	466.515 Mb	477.638 Mb	484.463 Mb
N50	21 Mb	20 Mb	34 Mb

Surprisingly, the karyotype of the last male was quite different, with a modal number of 84 mitotic chromosomes. Interestingly, there was the same number ($n = 28$) of elements as above at the pachynema stage, indicating that they were trivalents. They were thicker than bivalents and a more careful analysis showed the recurrent asynapsis of one of the 3 chromosomes (Supplementary Fig. 3). No "normal" metaphase I or II was observed. It was concluded that this specimen was triploid with $3n = 84$, and probably sterile. In *M. deidamia*, the diploid chromosome number had a discreet mode of $2n = 54$, suggesting a slightly smaller number of chromosome pairs ($n = 27$) in this more distantly-related species. Our result are consistent with the modal number of chromosomes in the Morphinae ($n = 28$) described in previous karyotypic studies conducted in 8 Morpho species [48], where the reported number of chromosomes was also $n = 28$ for both *M. helenor* and *M. achilles*.

GenomeScope analyses suggested very high levels of heterozygosity for the three species (Table 1). In all of them, the N50 and contig sizes were generally larger in the assemblies produced by Hifiasm than in IPA and Flye assemblies (see supplementary table 1). The BUSCO scores revealed a very high percentage of duplicated sequences, especially in the assemblies produced by IPA and Flye. The use of purge_dups strongly reduced the number of duplicates, the estimated size of the genome and the number of final contigs (see Supplementary Fig. 4 and Sup. Table 1). Hifiasm and the post treatment with Purge_dups v1.2.5 gave an assembly of 143 contigs for *M. helenor* (size of the longest contig: 42411663 bp), of 32 contigs for *M. achilles* (size of the longest contig: 24854087 bp) and of 58 contigs for *M. deidamia* (size of the longest contig: 22518629 bp) (sup. Table 1). The Rebaler pipeline identified a circular mitochondrial genome of 15,336 bp for the species *M. helenor*, 15,340 bp for *M. achilles* and 15,196 bp for *M. deidamia*.

Annotation of repetitive region

In each of the three species of *Morpho*, we annotated around 50% of the genome as repeated elements (Supplementary Fig. 5). In *M. helenor*, 241,166,073 bp (51.28% of the genome) corresponded to repeated elements, 261,488,514 bp (54.65% of the genome) in *M. achilles* and 255,779,512bp (52.75% of the genome) in *M. deidamia*. The repetitive elements categories are shown in Supplementary Fig. 5. For the three species, long interspersed nuclear elements (LINE's) accounted for the largest percentage (between 13.53% and 17.22%) of the repeated elements in the genomes.

Genome annotation

We recovered 12,651, 12,978 and 12,093 protein-coding genes in the genomes of *M. helenor*, *M. achilles* and *M. deidamia* respectively. These values are comparable to what was found in *Maniola hyperantus* (13,005 protein-coding genes) and *P. aegeria* (13,515 protein-coding genes), but were lower than in *M. jurtina* (13,777 protein-coding genes) and *B. anynana* (14,413 protein-coding genes). Busco results for the proteome and transcriptome are presented as supplementary material (see Supplementary Fig. 5 and 6) In order to assess if the annotations were complete, we estimated in each species the percentage of proteins with a Pfam domain as this value has been found to vary between 57% and 75% in eukaryotes [49]. This value ranged from 65,50% in *M. achilles* to 71,32% in *M. helenor* with an intermediate value of 70,42% in *M. deidamia*, thus showing that the annotations were of good quality. Proteome completeness using BUSCO was also high. From a set of 5286 single-copy orthologues from the lepidoptera lineage, the proteome completeness varied between 69% and 79% depending on the species (Supplementary Fig. 5). We were thus able to further investigate gene families that could be involved in pre-zygotic isolation through duplication or loss events. This includes genes having a role in vision (*L-opsin*) but also chemosensory genes such as odorant and gustatory receptors that reflect the degree of species specialization.

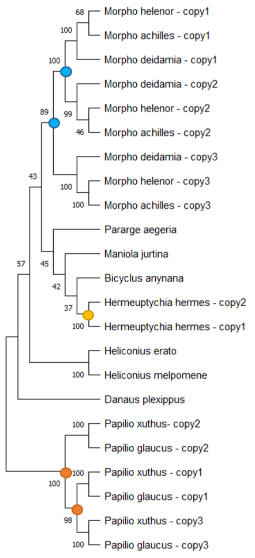

Figure 2. Maximum Likelihood tree of L-opsin exon sequences detected in the genomes of *M. helenor*, *M. achilles* and *M. deidamia* and other butterflies species, with bootstrap values. The colored dots indicate the putative locations of the duplication events on the tree: the putative origin of duplications of the L-opsin observed within the genus *Morpho* appear in blue, while the duplications that occured in the *Hermeuptychia hermes* clade and in the *Papilio* clade appear in yellow and orange respectively

Duplications in opsins genes

Vision in butterflies notably relies on opsins, for which three major types of molecules have been described depending on their wavelength of peak absorbance: in the ultraviolet (UV, 300-400 nm), blue (B, 400-500 nm) and long wavelength (L, 500-600 nm) part of the visible spectrum. Opsins are encoded by UV, B and L opsin genes. We investigated the number of copies for each opsin gene in the three *Morpho* species. We consistently found one copy of the UV opsin gene and two copies of the B opsin genes in the three *Morpho* species. Duplications of the L-opsin were observed in *M. achilles*, *M. deidamia* and *M. helenor*. In the other reference genomes *M. jurtina*, *B. anynana* and *P. aegeria*, a single copy of the UV opsin gene, the B opsin gene and the L opsin gene were found. By comparing the L-opsin sequences using a maximum likelihood tree based on the exon sequences (Fig. 2), we showed that the duplications observed in *Morpho* butterflies probably occurred independently from previously described duplications that happened in other clades of Lepidoptera. The phylogenetic relationships between the copies in the three species reveal that the duplications observed in the three *Morpho* species probably occurred before their speciation (Fig. 2). The detection of the different copies in different species within the Morpho genus and in closely-related genus is now required to precisely characterize the evolutionary origin of these duplications.

Odorant and gustatory receptors

In order to estimate the number of *OR* and *GR* genes in the three *Morpho* species, we blasted our Maker-annotated genes on the reference genome of *Spodoptera littoralis*. In this moth species, 60 *OR* and 16 *GR* genes were curated [50]. Interestingly, we recovered only 31 *OR* genes including Orco in *M. helenor*, 32 in *M. achilles* and 36 in *M. deidamia*, while we found 14 *GR* genes in *M. helenor* and 16 in *M. achilles* and *M. deidamia*. With insectOR, we found 36 OR genes including Orco in *M. helenor*, 37 in *M. achilles* and 38 in *M. deidamia*, confirming the major loss of ORs in our three Morpho species. For comparison we blasted against the same reference genome of *S. littoralis* the annotated sequences of the three outer Lepidopteran species used in the previous analyses and uncovered a much higher number of *OR* and *GR* genes with 61 *OR* and 28 *GR* in *M. jurtina*, 60 *OR* and 35 *GR* in *B. anynana* and 50 *OR* and 20 *GR* in *P. aegeria* respectively. The drastic reduction of chemosensory receptors, particularly in the number of *OR* genes in the three *Morpho* species could potentially reflect a higher degree of specialization to their respective biochemical environment. A phylogenetic analysis of *Morpho* ORs along with those of *S. littoralis*, the sole Lepidopteran species for which a considerable number of ORs were functionally deorphanized and divided into three chemical classes (aromatics, terpenes and aliphatics) as described in [34], showed that the loss of ORs in *Morpho* were not clustered around a particular set of genes (Supplementary Fig. 9). Further functional characterization coupled with precise ecological investigations are therefore needed to understand the loss of ORs in the *Morpho* genus.

Synteny and rearrangement detection

Conserved synteny with other Lepidoptera species

We found a high concordance between the $n = 29$ chromosomes of *M. jurtina* and the contigs of the three *Morpho* species (Fig. 3). The MUMmer alignment and the post alignment treatment to remove short contigs and low identity alignments reduced the assembly to 27 contigs containing 97% of the total genome for *M. helenor* (removing 117 short contigs from the original assembly), 29 contigs (98% of the genome) for *M. achilles* (3 contigs removed) and 27 for *M. deidamia* (31 contigs removed) (Fig. 3).

The synteny plot between *M. helenor* and *M. jurtina* showed 27 contigs for *M. helenor*, one contig less than expected based on its karyotype of n=28. In the plot, one single contig (ptg000028l) was assigned to two different chromosomes from the *M. jurtina* assem-

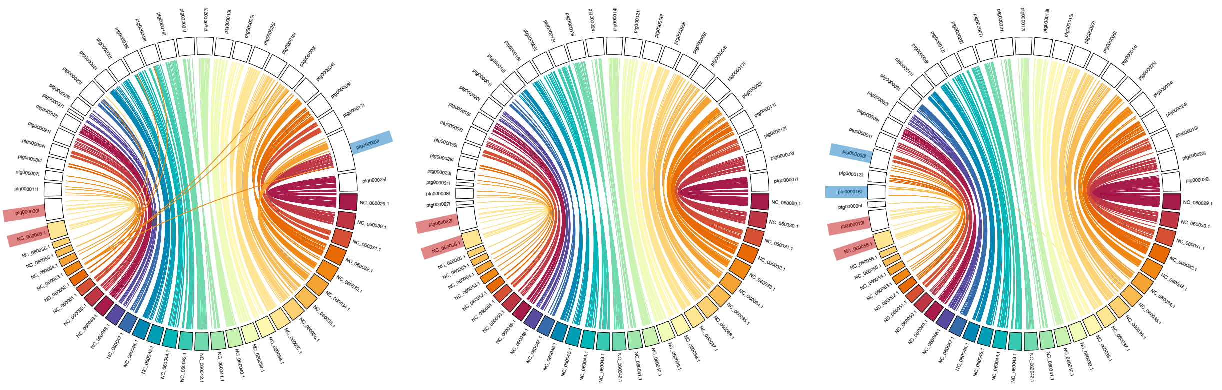

a) *M. jurtina vs M. helenor* b) *M. jurtina vs M. achilles* c) *M. jurtina vs M. deidamia*

Figure 3. Synteny between the chromosome-assembled genome of *Maniola jurtina* (colored chromosomes) and the genome assemblies of the species *Morpho helenor* (a),*M. achilles* (b) and *M. deidamia* (c). Equivalent chromosomes/contigs are linked by same color ribbons. Chromosome Z for each species is labeled in red. Single chromosomes in *Morpho* that are not assigned to a single chromosome in *M. jurtina* are labeled in blue

bly (chromosomes 2 and 6, NC_060030.1 and NC_060034.1). Contig ptg000028l is twice the size of any other contig found in the three *Morpho* species analyzed here. Based in the differences between the number of contigs and the karyotype and the unexpectedly big size of the contig ptg000028l, we believe the difference in chromosome number between *M. jurtina* and *M. helenor* can be explained by an overassembly of the genome of *M. helenor* by hifiasm, that assigned one single contig to two different chromosomes from the *M. jurtina* assembly (Fig. 3). In *M. deidamia*, the Hifiasm assembly showed a single contig ptg000008l (size 20.29 Mb) containing chromosomes NC_060051.1 and NC_060052.1 (sizes 10.05 Mb and 9.43 Mb respectively) from *M. jurtina*. Because the number of contigs recovered for this species is in accordance to the karyotype of n=27, the differences between *M. deidamia* and *M. jurtina* suggest that in this case chromosomes NC_060051.1 and NC_060052.1 in *M. jurtina* may have fused to form contig ptg000008l in *M. deidamia*. Other rearrangement in this species compared to *M. jurtina* seems to be the contig ptg0000161l, that appears to contain small portions of chromosomes NC_060054.1 and NC_060055.1 from *M. jurtina*.

For the three *Morpho* species, we were able to identify a single contig corresponding to the chromosome Z (NC_060058.1) in *M. jurtina* (contig ptg000030l in *M. helenor*, contig ptg000024l in *M. achilles* and contig ptg000019l in *M. deidamia*).

We also found a high level of colinearity between the genomes of the three *Morpho* species and the five Nymphalidae species used for comparisons. The alignment between *M. jurtina* and the three *Morpho* species (Fig. 3) was very similar to the alignments obtained for the other Nymphalidae (Supplementary Fig. 6) and confirmed that the assembly of the genome of *M. helenor* by hifiasm might have merged together two chromsomes: the single contig ptg000028l was scattered into two chromosomes in the other Nymphalidae. Although collinearity was generally high, we detected some putative inversions located in regions that varied among pairs for the three *Morpho* species in comparison with the Nymphalidae (see Supplementary Fig. 6). Interestingly, the contig corresponding to the chromosome Z was the only one consistently showing inversions in the pairwise genome-wide alignments (see Supplementary Fig. 6).

Inversions in the Z-chromosome between the three sympatric Morpho species

Pairwise whole genome alignments of the three *Morpho* species showed a very high similarity between genomes (see Supplementary Fig. 7). The only contig that differed between species was the one corresponding to the Z chromosome. SyRI identified one inversion of 1.6 Mb between *M. helenor* and *M. deidamia*, five inversions (comprising one of more than 1.8 Mb) between *M. helenor* and *M. achilles* and two between *M. deidamia* and *M. achilles* with one of 1.6 Mb (Fig. 4). Interestingly, the inversion found in *M. deidamia* when compared to *M. achilles* or *M. helenor* has the same size and is located in exactly the same position of the chromosome (from bp 1567583 to 3192401), suggesting that this inversion is ancestral to the speciation of *M. achilles* and *M. helenor*. In the case of *M. achilles vs. M. helenor* two inversions were found flanking the site of the putative ancient inversion and a bigger inversion was found at the end of the chromosome (Fig. 4).

Discussion

Assembly of heterozygous Lepidoptera genomes with a high proportion of repeated elements

We generated *de novo*, reference-quality genome assemblies for three emblematic species of Amazonian butterflies: *M. helenor*, *M. achilles* and *M. deidamia*. Our results indicate genome sizes comprised between 470 Mb and 489 Mb, similarly to most of the closely-related Nymphalidae species sequenced so far, *e.g. B. anynana* (475 Mb), *P. aegeria* (479 Mb) or *M. jurtina* (429 Mb). This is also close to the 479 Mb estimated from phylogenetic comparison using the taxon-centred database "Genomes on a Tree" (GoaT) [51]. The final number of contig within each of the three species ranged from 27 to 29, close to the number of chromosome pairs observed in our cytogenetics study. The numbers of chromosomes found in

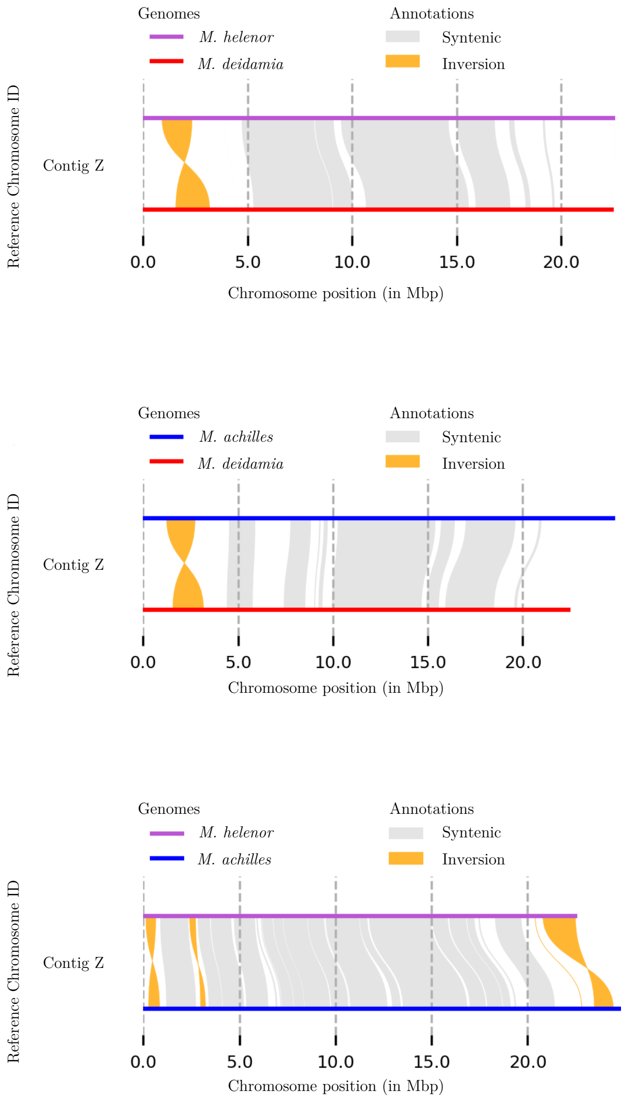

Figure 4. Synteny and rearrangement (SyRI) plot of the paired comparisons for the Z contig between the three *Morpho* species. Upper figure: *M. helenor* and *M. deidamia*; middle: *M. deidamia* and *M. achilles*; lower: *M. helenor* and *M. achilles*.

those French Guiana samples (*i.e.* in the subspecies *M. helenor helenor* and *M. achilles achilles*) is consistent with those found in other subspecies of both species in previous studies [48]. The available sequenced species of Nymphalidae that are closely-related to the genus *Morpho* also generally show 29 pairs of chromosomes (28 autosomes, plus Z and W sex chromosomes), which is close to the chromosomal numbers observed in the three *Morpho* species studied here. The mapping between the assemblies of *Morpho* species to the chromosome-level assembly of *Maniola jurtina* and the post-treatment to eliminate small contigs allowed us to identify between 27 and 29 contigs in *Morpho* that were homologous to *Maniola jurtina* chromosomes, including the contig corresponding to the Z chromosome. This suggests a high conservation of chromosomal synteny among closely-related Nymphalidae species, which is consistent with the high level of synteny observed throughout the whole Lepidoptera clade [52]. In the three species, genome heterozygosity was very high (from 1.68% in *M. deidamia* to 3.35% in *Morpho helenor*) and heterozygosity presents a major challenge in *de novo* assembly of diploid genomes. Indeed, levels of heterozygosity of 1% or above are considered "moderate to high" and most assemblers struggle when two divergent haplotypes are sequenced together, as heterozygosity may impair the distinction of different alleles at the same locus from paralogs at different loci [53]. Then, final assemblies of heterozygous genomes are expected to be of poor-quality, highly fragmented and containing redundant contigs [54]. Hifiasm generated the most completely haplotype-resolved assemblies, nevertheless the level of heterozygosity clearly impacted the quality of the assemblies and a post treatment to remove duplicated sequences was necessary for the two most heterozygous genomes (*M. helenor* and *M. achilles*), showing the difficulty that heterozygosity still imposes to long-read heterozygosity-aware assemblers. Such a high heterozygosity has been observed in other genomes of Lepidoptera [31] and can be a signature of high effective population sizes. The wide Amazonian distribution of these species, and their flight activity could contribute to such high level of genetic diversity within population, because elevated dispersal contribute to increase gene flow within each species throughout their geographic range. Our results also showed that around 50% of the genomes of the sequenced *Morpho* was composed of repeated elements, a very high proportion as compared to other genomes of Lepidoptera. In Lepidoptera, TE content has been found to be correlated with genome size [55], but in the case of the three *Morpho* species studied here, the repeat content is higher than for other species with similar genome sizes such as the *Bombyx mori* moth, with a genome size estimated at 530 Mb and a TE content of 35% [56] or the more closely-related species *Bicyclus anynana* with a genome size of 475 Mb and a repeat content of 26% [31].

Structural variations between genomes of sympatric species

The karyotype and assembly analyses suggest some differences in chromosome number between the three sympatric *Morpho* species studied here, particularly between *M. deidamia* (27 chromosome pairs) *vs M. helenor* and *M. achilles* (28 chromosome pairs). Differences in chromosome numbers and other chromosomal rearrangements may strongly affect reproductive barriers. Two groups of models have been proposed to explain how chromosomal rearrangements prevent gene-flow and contribute to species maintenance and speciation. First, hybrid-sterility models suggest reduced fertility or viability in individuals heterozygous for chromosomal rearrangements. These models are considered to be inconsistent and difficult to evaluate [4]. More recently, suppressed-recombination models propose that chromosomal rearrangements permit speciation in sympatry because they reduce recombination between chromosomes carrying different rearrangements [4]. Indeed, in Lepidoptera, differences in chromosome number are proposed to be an important mechanism leading to species diversification in *Agrodiaetus*, *Erebia* and *Lysandra* butterflies (57, 58, 59).

Besides differences in chromosome numbers, we systematically found inversions in the contig corresponding to the Z chromosome when comparing the genomes of *Morpho* to the other Nymphalidae and between the three different *Morpho* species. Inversions are also a type of chromosomal rearrangement known to occur throughout evolution and are considered an important mechanism for speciation particularly for species living in sympatry (1, 4). Empirically and theoretically, it has been suggested that inversions may have contributed to speciation in sympatry in different groups of animals. In two ascidians species of the genus *Ciona* and in insects like *Drosophila* inversions may promote speciation by reduction of the fitness or by causing sterility of heterozygotes (60, 61). In the *Anopheles gambiae* species complex, inversions may allow for ecotypic differentiation and niche partitioning leading to different sympatric and genetically isolated populations [62]. In groups like paserine birds where sexual differentiation is controlled by a ZW sex chromosome system (females being the heterogametic sex), inversions in the Z chromosome in particular seem to explain speciation in sympatry between close species. Cytological data show that across the Passeriformes, the Z chromosome has accumulated more inversions than any other autosome and that the inversion

fixation rate on the Z chromosome is 1.4 times greater than the average autosome. Interestingly, inversions on the Z chromosome are significantly more common in sympatric than in allopatric closely related clades (63, 64).

In Lepidoptera, the role of inversions in speciation in sympatry has been studied in the species *Heliconius melpomene* and *H. cydno*, two sympatric species that can hybridize (although rarely) in the wild [65]. The analyses of the genomic differences between the two species showed some small inversions (less than 50 kb) and there was no evidence for a reduction of recombination in hybrids, suggesting that in this case, inversions were not involved in the maintenance of the species barriers and other processes such as strong mate preference could prevent hybridisation in the wild [65]. In the *Morpho* species studied here however, we found inversions between *Morpho* Z chromosomes that were longer than 1.5 Mb. Models suggest that to be associated with adaptive traits or species barriers, inversions should typically be megabases long in order to be fixed in populations [65]. The position of the inversion in the Z contig when comparing *M. helenor* or *M. achilles* to *M. deidamia* is at the exact same place in *M. deidamia*'s genome, suggesting that this specific inversion likely occurred before the speciation between *M. achilles* and *M. helenor*. When comparing *M. helenor* to *M. achilles*, we found two different smaller inversions that are not found in *M. deidamia* and that are close to the putative ancestral inversion region, suggesting that these two smaller inversions could have appeared after the speciation between *M. achilles* and *M. helenor*. At the moment, we do not know if the inversions segregate at different frequencies in the *Morpho* populations or if they are fixed. Population analyses are needed to answer this question and to enlighten what evolutionary forces could be acting to maintain them. The copy number variation detected in genes involved in colour perception (*i.e. L-opsin*) may also play a significant role in reproductive isolation in these sympatric species. For instance, the three copies of L opsins found in the Papilio genus (Fig. 2) have been found to also show subfunctionalization and neofunctionalization [66]. The duplication followed by genetic divergence observed in these three mimetic *Morpho* species may improve their visual discrimination capacities, and facilitate species recognition, therefore reinforcing barriers to gene flow in sympatry. Genes potentially involved in colour pattern variations (*e.g.* $bric-a-brac$ or *bab*) may also play a role in prezygotic isolation but they were not thoroughly investigated here as their functional evolution involves changes in regulatory sequences rather than events of duplication or gene loss [67]. Interestingly, an orthologous search of the putative proteic sequences of each *Morpho* species against those of *M. jurtina* allowed us to uncover different copy numbers of the gene $bric-a-brac$, which play a significant role in differences of UV iridescence between males of two incipient species of sulphur butterflies [68]. The copy responsible for the presence/absence of UV iridescence is located on the Z chromosome and in the three *Morpho* species, we found one or more copies of $bric-a-brac$ on the contigs that correspond to the Z chromosome: *M. deidamia* had one copy of $bric-a-brac$, while *M. helenor* and *M. achilles* displayed two copies of this gene. It seems however that the second copy in *M. helenor* and *M. achilles* correspond to truncated copies of $bric-a-brac$. While this is certainly the sign of an ancient duplication followed by a pseudogenization event, this could lead to further investigations of putative functions of the truncated copies. It is worth noting that variations in the number of *bab* copies was also observed in the three reference genomes used for the blast: *M. jurtina* had two copies on the Z chromosome (including a truncated copy), *B. anynana* had only one and *P. aegeria* had none. The investigation of gene levels of polymorphism on the Z chromosomes would also be of great interest as genes linked to the Z chromosome are often among the most divergent between closely-related species [69].

Altogether, the assembly and annotation of these three mimetic species of *Morpho* butterflies reveal differences in chromosome numbers, the presence of several Mb-long inversions in the Z chromosome, as well as copy number variation and genetic divergence among copies of genes that may play a significant role in reproductive isolation. Our study thus open new avenues into the investigation of the ecological and genomic factors involved in sympatric speciation and its reinforcement.

Availability of supporting data and materials

Fastq files, genome assemblies, assembly methods and collection information were uploaded at the ENA web site (https://www.ebi.ac.uk/ena/browser/home) under the project number PRJEB56642. Genome accession numbers are ERZ14213098 for *Morpho helenor*, ERZ14213099 for *M. achilles* and ERZ14213100 for *M. deidamia*. Transcriptomic and proteic sequences of predicted genes as well as gff files are available for each species upon request.

Ethical Approval

All butterflies used in this study were sampled in French Guiana and used by researchers from French Institutions. Following the recommendation of the Nagoya protocol, our results were presented to the Université Antilles/Guyane in French Guiana during a seminar by VL. VL and VD also produced a popularization movie and performed several talks in public school from French Guiana, insuring a feed-back from our research on natural resources toward local populations.

Consent for publication

Not applicable

Competing Interests

The authors do not declare any competing interests.

Funding

This work was funded by three programs provided to VL as a PI: the MITI program from the CNRS, the ATM program from the Museum National d'Histoire Naturelle (Paris, France), and the ANR-T-ERC-OUTOFTHEBLUE from the French National Research Agency.

Author's Contributions

VL and VD collected the butterflies in the wild. AMD performed the karyotype analyses. VL and DO extracted the DNA. JL performed the PacBio sequencing. MLV and DO performed the genome assembly. MLV performed the TE analyses. MLV and HB performed the structural variation detection. HB performed the genome annotations. VL supervised the whole project. All authors contributed to the writing of the manuscript.

Acknowledgements

The authors would like to thank Patrick Blandin for his continuous support on our *Morpho* studies. They also thank Mélanie McClure, Mathieu Chouteau, Camille Le Roy and Ombeline Sculfort for their help during field work in French Guiana. We thank Elise Gay, Romuald Laso-Jadart, Pierre Lesturgie, Christelle Fraisse, Clément Gilbert and Quentin Rougemont for help with some scripts and for discussions of previous versions of the manuscript. We thank Charlotte J. Wright and Niklas Wahlberg for their careful reading of our manuscript and their many insightful comments and suggestions. We are thankful to the Plateforme de Calcul Intensif

et Algorithmique PCIA, Muséum national d'histoire naturelle, Centre national de la recherche scientifique, UAR 2700 2AD, CP 26, 57 rue Cuvier, F-75231 Paris Cedex 05, France and the Genotoul bioinformatics platform Toulouse Occitanie (Bioinfo Genotoul, https://doi.org/10.15454/1.5572369328961167E12).

References

1. Kirkpatrick M, Barton N. Chromosome inversions, local adaptation and speciation. Genetics 2006;173(1):419–434.
2. De-Kayne R, Selz OM, Marques DA, Frei D, Seehausen O, Feulner PGD. Genomic architecture of adaptive radiation and hybridization in Alpine whitefish. NATURE COMMUNICATIONS 2022 AUG 2;13(1).
3. Hoffmann AA, Sgrò CM, Weeks AR. Chromosomal inversion polymorphisms and adaptation. Trends in Ecology & Evolution 2004;19(9):482–488.
4. Faria R, Navarro A. Chromosomal speciation revisited: rearranging theory with pieces of evidence. Trends in ecology & evolution 2010;25(11):660–669.
5. Mérot C, Oomen RA, Tigano A, Wellenreuther M. A roadmap for understanding the evolutionary significance of structural genomic variation. Trends in Ecology & Evolution 2020;35(7):561–572.
6. Blandin P, Purser B. Evolution and diversification of Neotropical butterflies: Insights from the biogeography and phylogeny of the genus Morpho Fabricius, 1807 (Nymphalidae: Morphinae), with a review of the geodynamics of South AAmerica. Tropical Lepidoptera Research 2013;p. 62–85.
7. Allio R, Nabholz B, Wanke S, Chomicki G, Pérez-Escobar OA, Cotton AM, et al. Genome-wide macroevolutionary signatures of key innovations in butterflies colonizing new host plants. Nature communications 2021;12(1):1–15.
8. Briscoe AD, Macias-Munoz A, Kozak KM, Walters JR, Yuan F, Jamie GA, et al. Female behaviour drives expression and evolution of gustatory receptors in butterflies. PLoS genetics 2013;9(7):e1003620.
9. Naisbit RE, Jiggins CD, Mallet J. Disruptive sexual selection against hybrids contributes to speciation between Heliconius cydno and Heliconius melpomene. Proceedings of the Royal Society of London Series B: Biological Sciences 2001;268(1478):1849–1854.
10. Smadja C, Butlin R. On the scent of speciation: the chemosensory system and its role in premating isolation. Heredity 2009;102(1):77–97.
11. Debat V, Berthier S, Blandin P, Chazot N, Elias M, Gomez D, et al. Why are Morpho Blue? In: Biodiversity and evolution Elsevier; 2018.p. 139–174.
12. Pinheiro C, Freitas A, Campos V, DeVries P, Penz C. Both palatable and unpalatable butterflies use bright colors to signal difficulty of capture to predators. Neotropical Entomology 2016;45(2):107–113.
13. Llaurens V, Le Poul Y, Puissant A, Blandin P, Debat V. Convergence in sympatry: Evolution of blue-banded wing pattern in Morpho butterflies. Journal of Evolutionary Biology 2021;34(2):284–295.
14. Boussens-Dumon G, Llaurens V. Sex, competition and mimicry: an eco-evolutionary model reveals unexpected impacts of ecological interactions on the evolution of phenotypes in sympatry. Oikos 2021;130(11):2028–2039.
15. Le Roy C, Roux C, Authier E, Parrinello H, Bastide H, Debat V, et al. Convergent morphology and divergent phenology promote the coexistence of Morpho butterfly species. Nature communications 2021;12(1):1–9.
16. de Vos JM, Augustijnen H, Bätscher L, Lucek K. Speciation through chromosomal fusion and fission in Lepidoptera. Philosophical Transactions of the Royal Society B 2020;375(1806):20190539.
17. Chazot N, Blandin P, Debat V, Elias M, Condamine FL. Punctuational ecological changes rather than global factors drive species diversification and the evolution of wing phenotypes in Morpho butterflies. Journal of Evolutionary Biology 2021;34(10):1592–1607.
18. McClure M, Dutrillaux B, Dutrillaux AM, Lukhtanov V, Elias M. Heterozygosity and chain multivalents during meiosis illustrate ongoing evolution as a result of multiple holokinetic chromosome fusions in the genus Melinaea (Lepidoptera, Nymphalidae). Cytogenetic and Genome Research 2017;153(4):213–222.
19. Marcais G, Kingsford C. A fast, lock-free approach for efficient parallel counting of occurrences of k-mers. BIOINFORMATICS 2011 MAR 15;27(6):764–770.
20. Ranallo-Benavidez TR, Jaron KS, Schatz MC. GenomeScope 2.0 and Smudgeplot for reference-free profiling of polyploid genomes. NATURE COMMUNICATIONS 2020 MAR 18;11(1).
21. Kolmogorov M, Yuan J, Lin Y, Pevzner PA. Assembly of long, error-prone reads using repeat graphs. NATURE BIOTECHNOLOGY 2019 MAY;37(5):540+.
22. Cheng H, Concepcion GT, Feng X, Zhang H, Li H. Haplotype-resolved de novo assembly using phased assembly graphs with hifiasm. NATURE METHODS 2021 FEB;18(2):170+.
23. Bushnell B. BBMap: A Fast, Accurate, Splice-Aware Aligner.[WWW document]. URL https://www osti gov/se rvlets/purl/1241166 2014;.
24. Manni M, Berkeley MR, Seppey M, Simao FA, Zdobnov EM. BUSCO Update: Novel and Streamlined Workflows along with Broader and Deeper Phylogenetic Coverage for Scoring of Eukaryotic, Prokaryotic, and Viral Genomes. MOLECULAR BIOLOGY AND EVOLUTION 2021 OCT;38(10):4647–4654.
25. Guan D, McCarthy SA, Wood J, Howe K, Wang Y, Durbin R. Identifying and removing haplotypic duplication in primary genome assemblies. BIOINFORMATICS 2020 MAY 1;36(9):2896–2898.
26. Flynn JM, Hubley R, Goubert C, Rosen J, Clark AG, Feschotte C, et al. RepeatModeler2 for automated genomic discovery of transposable element families. PROCEEDINGS OF THE NATIONAL ACADEMY OF SCIENCES OF THE UNITED STATES OF AMERICA 2020 APR 28;117(17):9451–9457.
27. Cantarel BL, Korf I, Robb SMC, Parra G, Ross E, Moore B, et al. MAKER: An easy-to-use annotation pipeline designed for emerging model organism genomes. Genome Research 2008;18(1):188–196. http://genome.cshlp.org/content/18/1/188.abstract.
28. Muller H, Ogereau D, Da Lage JL, Capdevielle C, Pollet N, Fortuna T, et al. Draft nuclear genome and complete mitogenome of the Mediterranean corn borer, *Sesamia nonagrioides*, a major pest of maize. G3 Genes|Genomes|Genetics 2021 05;11(7). https://doi.org/10.1093/g3journal/jkab155, jkab155.
29. Ellis EA, Storer CG, Kawahara AY. *De novo* genome assemblies of butterflies. GigaScience 2021 06;10(6). https://doi.org/10.1093/gigascience/giab041, giab041.
30. Mead D, Saccheri I, Yung CJ, Lohse K, Lohse C, Ashmole P, et al. The genome sequence of the ringlet, *Aphantopus hyperantus* Linnaeus 1758. Wellcome Open Research 2021;6(165):165.
31. Nowell RW, Elsworth B, Oostra V, Zwaan BJ, Wheat CW, Saastamoinen M, et al. A high-coverage draft genome of the mycalesine butterfly *Bicyclus anynana*. GigaScience 2017 05;6(7). https://doi.org/10.1093/gigascience/gix035, gix035.
32. Korf I. Gene finding in novel genomes. BMC bioinformatics 2004;5(1):1–9.
33. König S, Romoth LW, Gerischer L, Stanke M. Simultaneous gene finding in multiple genomes. Bioinformatics 2016 07;32(22):3388–3395. https://doi.org/10.1093/bioinformatics/btw494.

34. De Fouchier A, Walker III WB, Montagné N, Steiner C, Binyameen M, Schlyter F, et al. Functional evolution of Lepidoptera olfactory receptors revealed by deorphanization of a moth repertoire. Nature communications 2017;8(1):15709.
35. Tamura K, Stecher G, Kumar S. MEGA11: molecular evolutionary genetics analysis version 11. Molecular biology and evolution 2021;38(7):3022–3027.
36. Slater GSC, Birney E. Automated generation of heuristics for biological sequence comparison. BMC bioinformatics 2005;6:1–11.
37. Eddy SR. Accelerated profile HMM searches. PLoS computational biology 2011;7(10):e1002195.
38. Finn RD, Coggill P, Eberhardt RY, Eddy SR, Mistry J, Mitchell AL, et al. The Pfam protein families database: towards a more sustainable future. Nucleic acids research 2016;44(D1):D279–D285.
39. Katoh K, Standley DM. MAFFT multiple sequence alignment software version 7: improvements in performance and usability. Molecular biology and evolution 2013;30(4):772–780.
40. Minh BQ, Schmidt HA, Chernomor O, Schrempf D, Woodhams MD, Von Haeseler A, et al. IQ-TREE 2: new models and efficient methods for phylogenetic inference in the genomic era. Molecular biology and evolution 2020;37(5):1530–1534.
41. Kurtz S, Phillippy A, Delcher AL, Smoot M, Shumway M, Antonescu C, et al. Versatile and open software for comparing large genomes. Genome biology 2004;5(2):1–9.
42. Gu Z, Gu L, Eils R, Schlesner M, Brors B. Circlize implements and enhances circular visualization in R. Bioinformatics 2014;30(19):2811–2812.
43. Celorio-Mancera MdlP, Rastas P, Steward RA, Nylin S, Wheat CW. Chromosome level assembly of the comma butterfly (Polygonia c-album). Genome biology and evolution 2021;13(5):evab054.
44. Cabanettes F, Klopp C. D-GENIES: dot plot large genomes in an interactive, efficient and simple way. PeerJ 2018;6:e4958.
45. Li H. Minimap2: pairwise alignment for nucleotide sequences. Bioinformatics 2018;34(18):3094–3100.
46. Goel M, Sun H, Jiao WB, Schneeberger K. SyRI: finding genomic rearrangements and local sequence differences from whole-genome assemblies. Genome biology 2019;20(1):1–13.
47. Goel M, Schneeberger K. plotsr: visualizing structural similarities and rearrangements between multiple genomes. Bioinformatics 2022;38(10):2922–2926.
48. Brown KS, Freitas AV, Von Schoultz B, Saura AO, Saura A. Chromosomal evolution of South American frugivorous butterflies in the Satyroid clade (Nymphalidae: Charaxinae, Morphinae and Satyrinae). Biological Journal of the Linnean Society 2007;92(3):467–481.
49. Yandell M, Ence D. A beginner's guide to eukaryotic genome annotation. Nature reviews Genetics 2012 April;13(5):329—342. https://doi.org/10.1038/nrg3174.
50. Walker WB, Roy A, Anderson P, Schlyter F, Hansson BS, Larsson MC. Transcriptome analysis of gene families involved in chemosensory function in Spodoptera littoralis (Lepidoptera: Noctuidae). BMC genomics 2019;20(1):1–20.
51. Gomes Sotero-Caio C, Challis R, Kumar S, Blaxter M. Genomes on a Tree (GoaT): A centralized resource for eukaryotic genome sequencing initiatives. Biodiversity Information Science and Standards 2021;5:e74138. https://doi.org/10.3897/biss.5.74138.
52. d'Alencon E, Sezutsu H, Legeai F, Permal E, Bernard-Samain S, Gimenez S, et al. Extensive synteny conservation of holocentric chromosomes in Lepidoptera despite high rates of local genome rearrangements. Proceedings of the National Academy of Sciences 2010;107(17):7680–7685.
53. Asalone KC, Ryan KM, Yamadi M, Cohen AL, Farmer WG, George DJ, et al. Regional sequence expansion or collapse in heterozygous genome assemblies. PLOS COMPUTATIONAL BIOLOGY 2020 JUL;16(7).
54. Pryszcz LP, Gabaldon T. Redundans: an assembly pipeline for highly heterozygous genomes. NUCLEIC ACIDS RESEARCH 2016 JUL 8;44(12).
55. Talla V, Suh A, Kalsoom F, Dinca V, Vila R, Friberg M, et al. Rapid Increase in Genome Size as a Consequence of Transposable Element Hyperactivity in Wood-White (Leptidea) Butterflies. GENOME BIOLOGY AND EVOLUTION 2017 OCT;9(10):2491–2505.
56. Osanai-Futahashi M, Suetsugu Y, Mita K, Fujiwara H. Genome-wide screening and characterization of transposable elements and their distribution analysis in the silkworm, Bombyx mori. INSECT BIOCHEMISTRY AND MOLECULAR BIOLOGY 2008 DEC;38(12):1046–1057.
57. Lucek K. Evolutionary Mechanisms of Varying Chromosome Numbers in the Radiation of Erebia Butterflies. GENES 2018 MAR;9(3).
58. Talavera G, Lukhtanov VA, Rieppel L, Pierce NE, Vila R. In the shadow of phylogenetic uncertainty: The recent diversification of Lysandra butterflies through chromosomal change. MOLECULAR PHYLOGENETICS AND EVOLUTION 2013 DEC;69(3):469–478.
59. Lukhtanov V, Kandul N, Plotkin J, Dantchenko A, Haig D, Pierce N. Reinforcement of pre-zygotic isolation and karyotype evolution in Agrodiaetus butterflies. NATURE 2005 JUL 21;436(7049):385–389.
60. Noor M, Grams K, Bertucci L, Reiland J. Chromosomal inversions and the reproductive isolation of species. PROCEEDINGS OF THE NATIONAL ACADEMY OF SCIENCES OF THE UNITED STATES OF AMERICA 2001 OCT 9;98(21):12084–12088.
61. Satou Y, Sato A, Yasuo H, Mihirogi Y, Bishop J, Fujie M, et al. Chromosomal Inversion Polymorphisms in Two Sympatric Ascidian Lineages. GENOME BIOLOGY AND EVOLUTION 2021 JUN;13(6).
62. Coluzzi M, Sabatini A, della Torre A, Di Deco M, Petrarca V. A polytene chromosome analysis of the Anopheles gambiae species complex. SCIENCE 2002 NOV 15;298(5597):1415–1418.
63. Hooper DM, Price TD. Chromosomal inversion differences correlate with range overlap in passerine birds. NATURE ECOLOGY & EVOLUTION 2017 OCT;1(10):1526–1534.
64. Hooper DM, Griffith SC, Price TD. Sex chromosome inversions enforce reproductive isolation across an avian hybrid zone. MOLECULAR ECOLOGY 2019 MAR;28(6, SI):1246–1262.
65. Davey JW, Barker SL, Rastas PM, Pinharanda A, Martin SH, Durbin R, et al. No evidence for maintenance of a sympatric Heliconius species barrier by chromosomal inversions. EVOLUTION LETTERS 2017 AUG;1(3):138–154.
66. Arikawa K. Spectral organization of the eye of a butterfly, Papilio. Journal of comparative physiology A, Neuroethology, sensory, neural, and behavioral physiology 2003 November;189(11):791—800. https://doi.org/10.1007/s00359-003-0454-7.
67. Rebeiz M, Williams TM. Using Drosophila pigmentation traits to study the mechanisms of *cis*-regulatory evolution. Current Opinion in Insect Science 2017;19:1–7. https://www.sciencedirect.com/science/article/pii/S2214574516301456.
68. Ficarrotta V, Hanly JJ, Loh LS, Francescutti CM, Ren A, Tunström K, et al. A genetic switch for male UV iridescence in an incipient species pair of sulphur butterflies. Proceedings of the National Academy of Sciences 2022;119(3):e2109255118. https://www.pnas.org/doi/abs/10.1073/pnas.2109255118.
69. Cong Q, Zhang J, Shen J, Cao X, Brévignon C, Grishin NV. Speciation in North American Junonia from a genomic perspective. Systematic Entomology 2020;45(4):803–837.

Figure 1

Click here to access/download;Figure;Figure_1.png

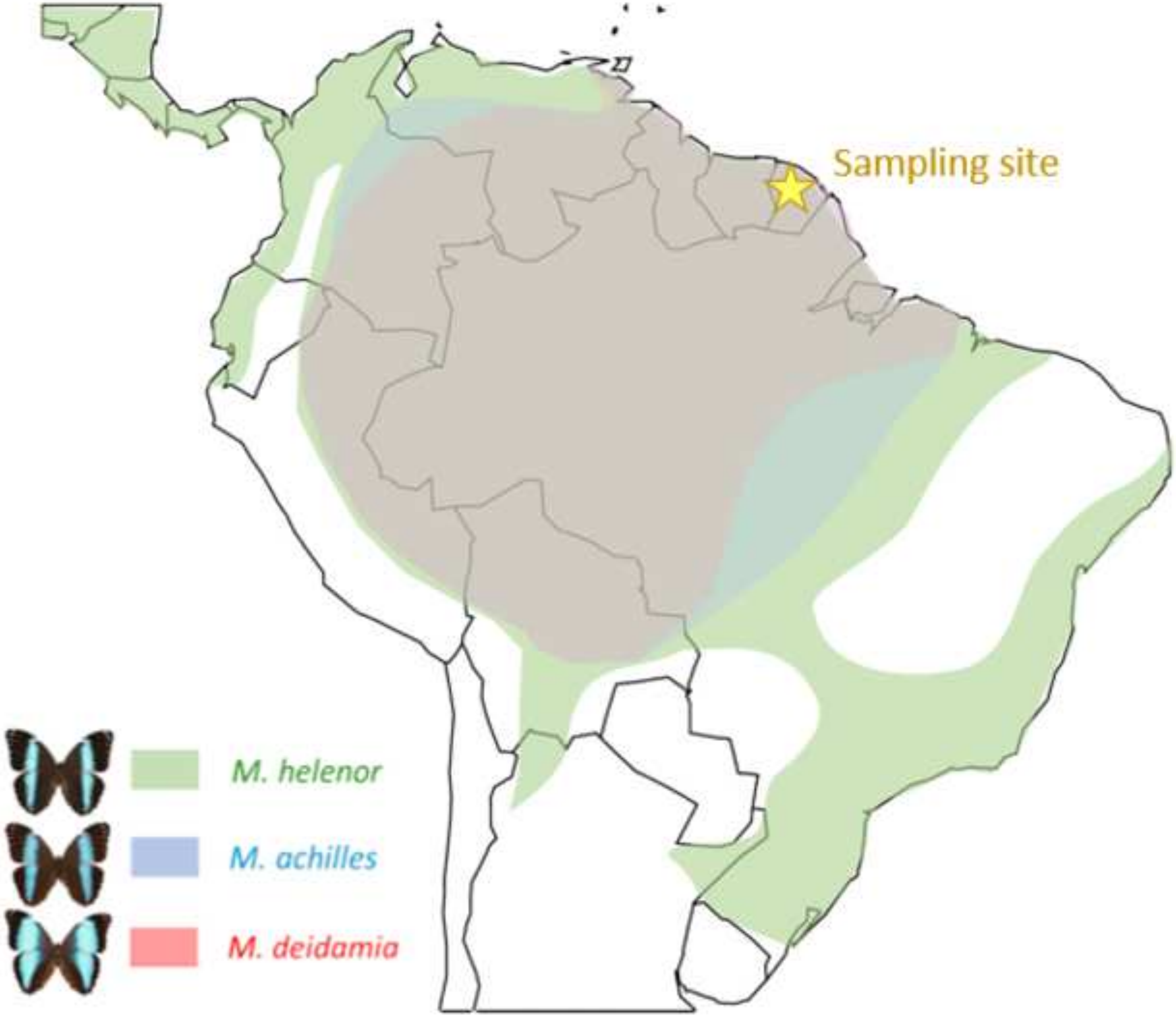

Figure 2

Click here to access/download;Figure;Figure_2.png

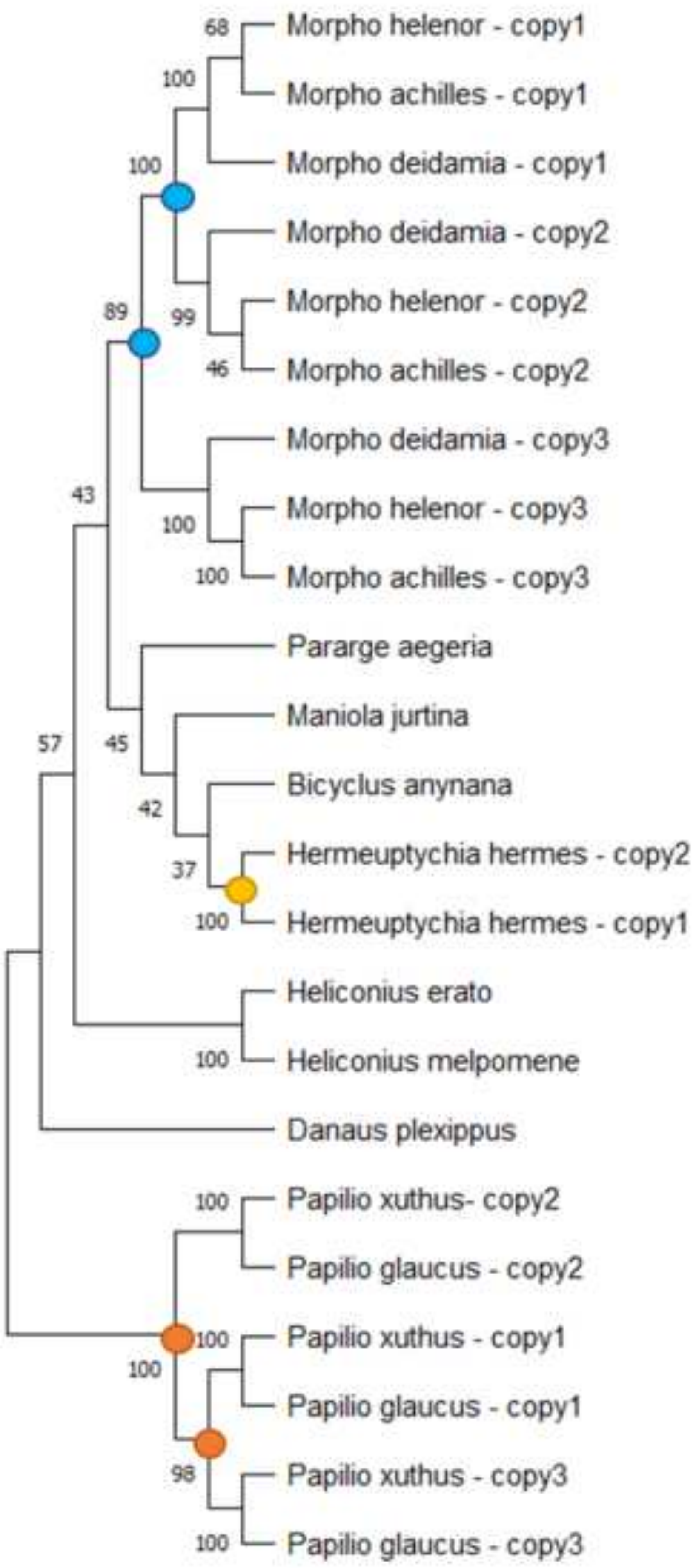

Figure 3

Click here to access/download;Figure;Figure_3.pdf

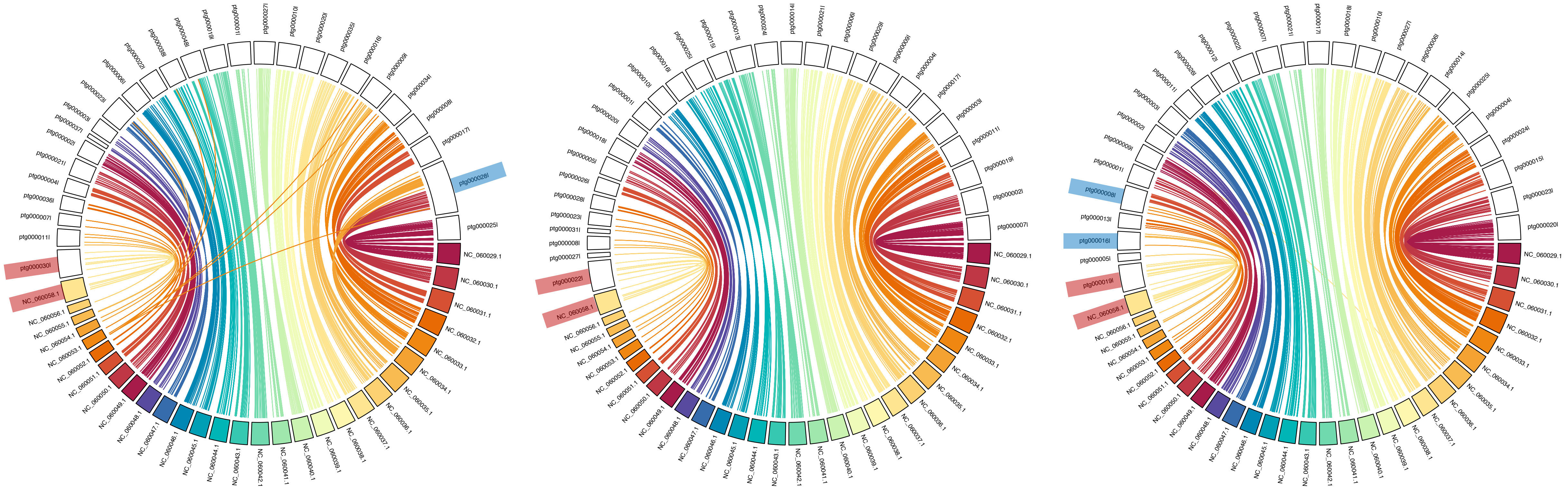

a) *M. jurtina vs M. helenor* b) *M. jurtina vs M. achilles* c) *M. jurtina vs M. deidamia*

Figure 4

Click here to access/download;Figure;Figure_4.pdf

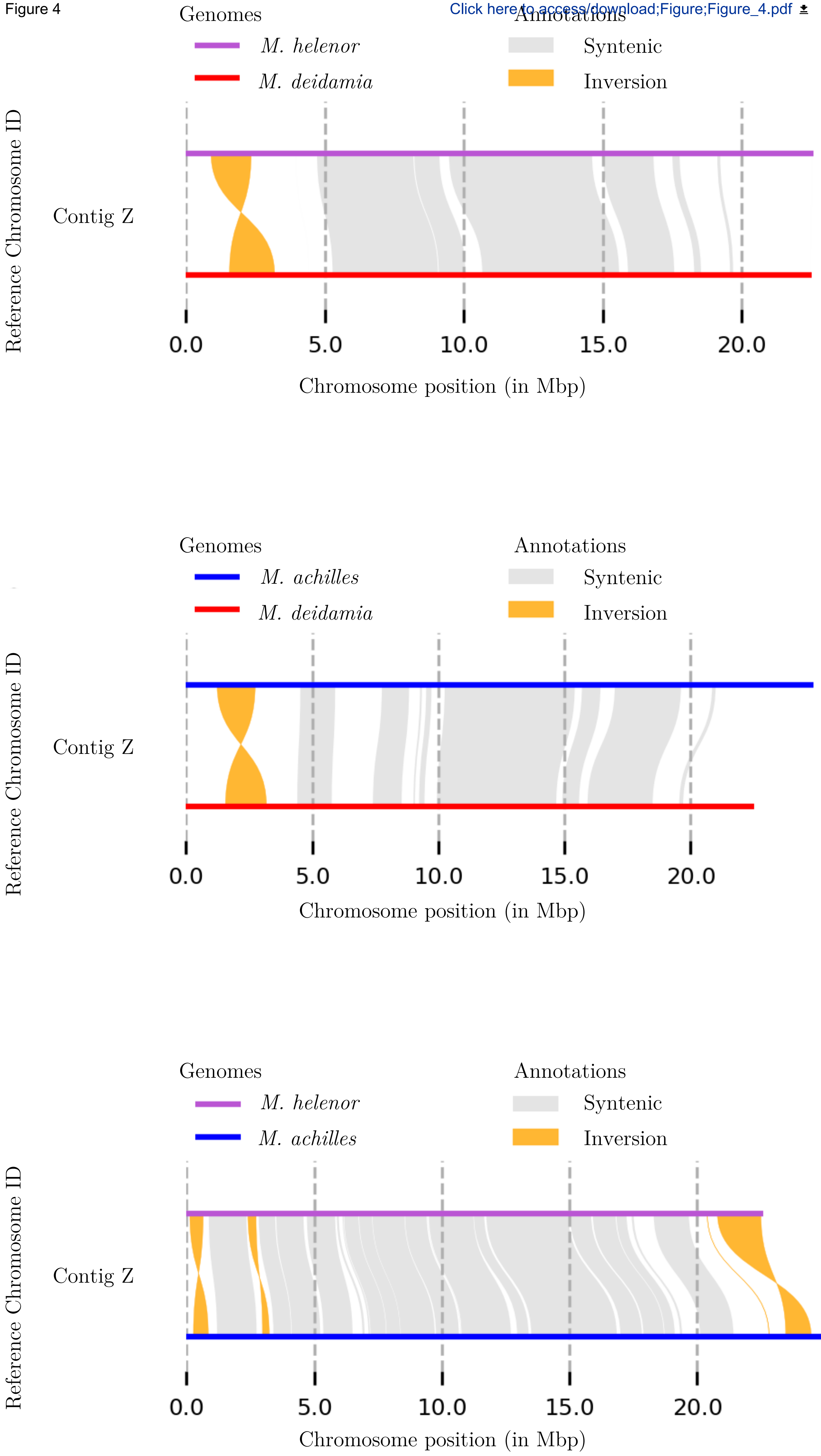

Click here to access/download
Supplementary Material
Supplementary_Material.pdf

Personal Cover Click here to access/download;Personal Cover;CoverLetter.docx

Dear editor,

We are very pleased to resubmit our manuscript entitled ‘Genome assembly of three Amazonian *Morpho* butterfly species reveals Z-chromosome rearrangements between closely-related species living in sympatry’ to Gigascience. We considered all reviewers’ comments and addressed them as it is found in the accompanying response. We hope this revision would be found suitable for publication in your journal.

Sincerely yours,

Héloïse Bastide, on behalf of all authors.
