# Supplementary figures and images for: Genome assembly of 3 Amazonian Morpho butterfly species reveals Z-chromosome rearrangements between closely related species living in sympatry
Source: Gigascience. 2023 May 22;12:giad033. doi: 10.1093/gigascience/giad033 (PMC10202424; doi:10.1093/gigascience/giad033)

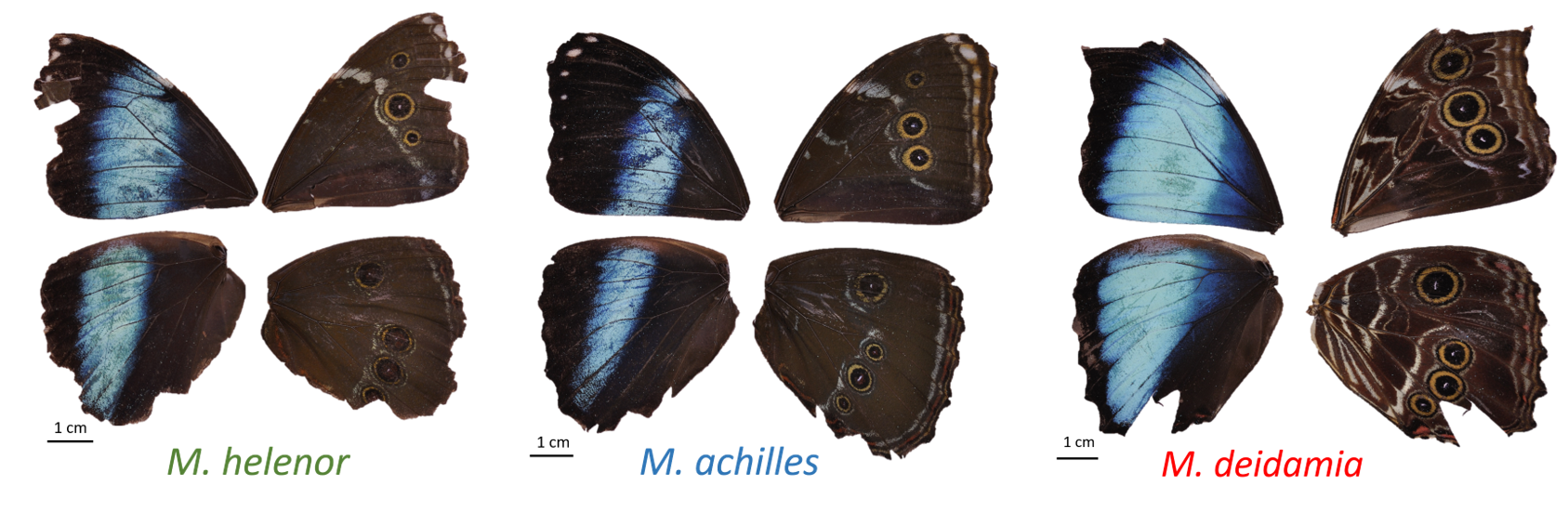

Supplement: giad033_Supplemental_Files [file giad033_supplemental_files.zip › Supp_Figure_1.png]

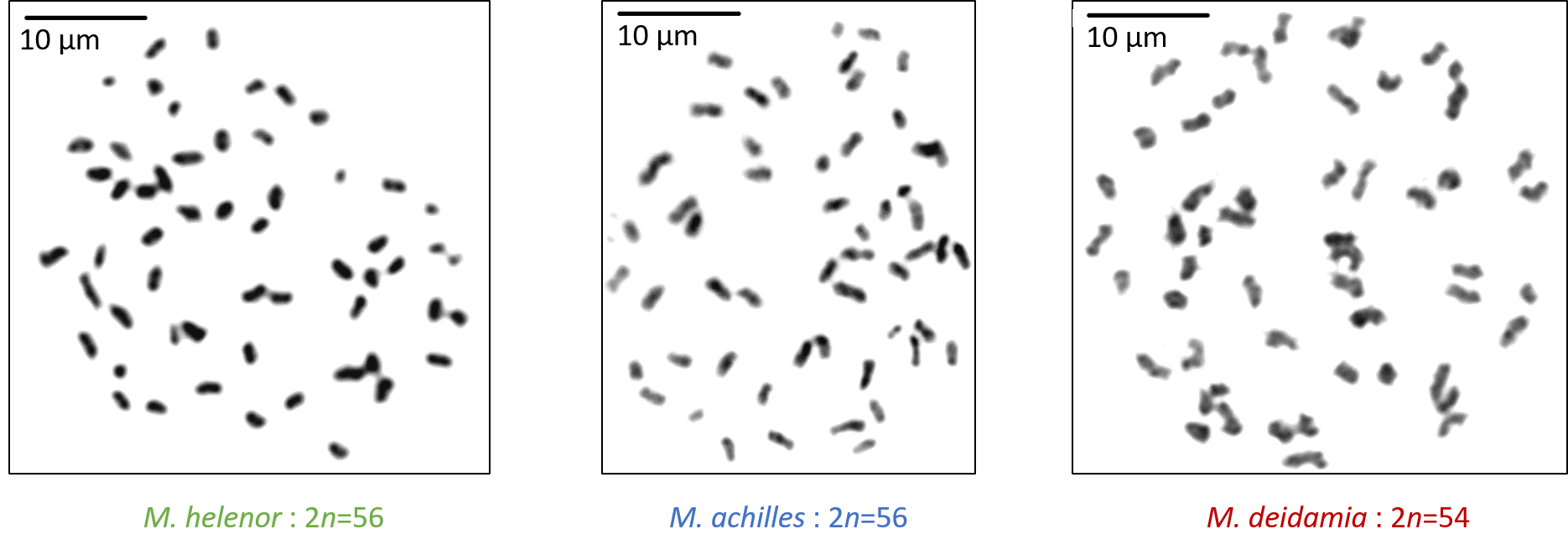

Supplement: giad033_Supplemental_Files [file giad033_supplemental_files.zip › Supp_Figure_2.png]

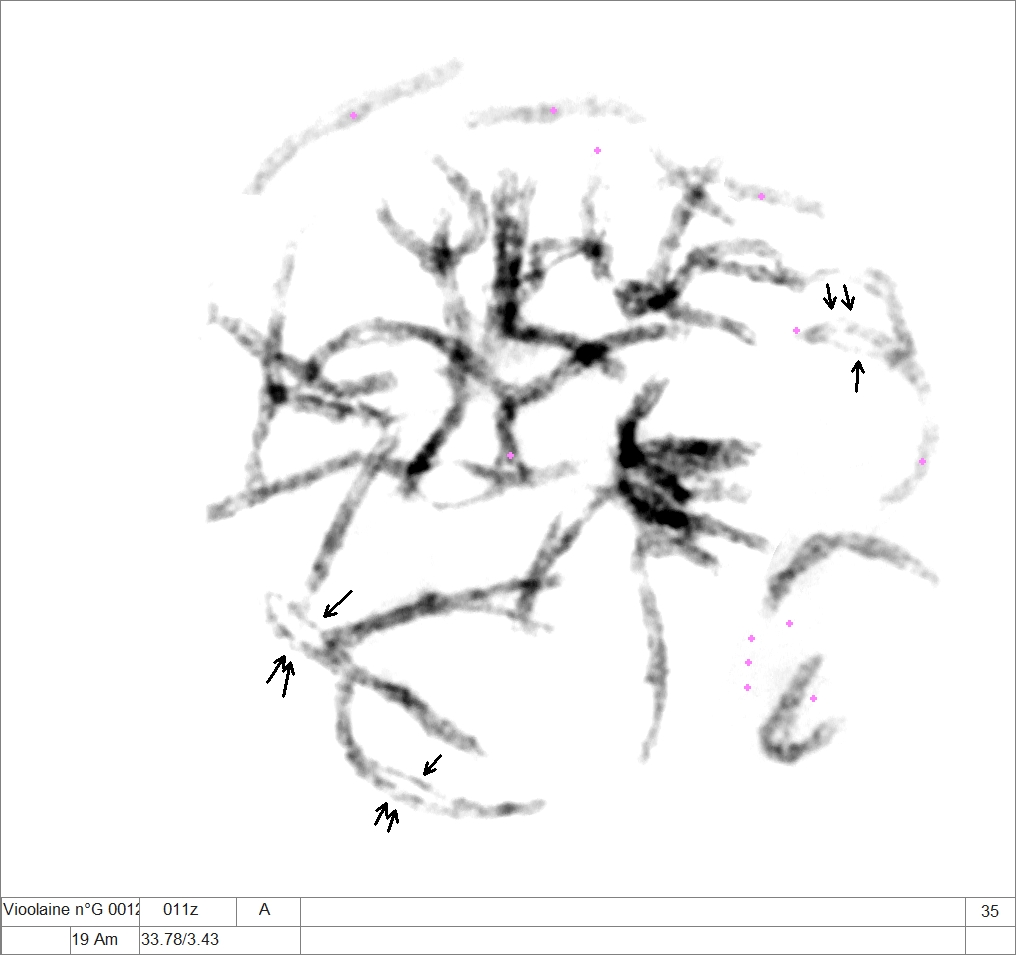

Supplement: giad033_Supplemental_Files [file giad033_supplemental_files.zip › Supp_Figure_3.JPG]

## BUSCO Assessment Results before Purge\_dups

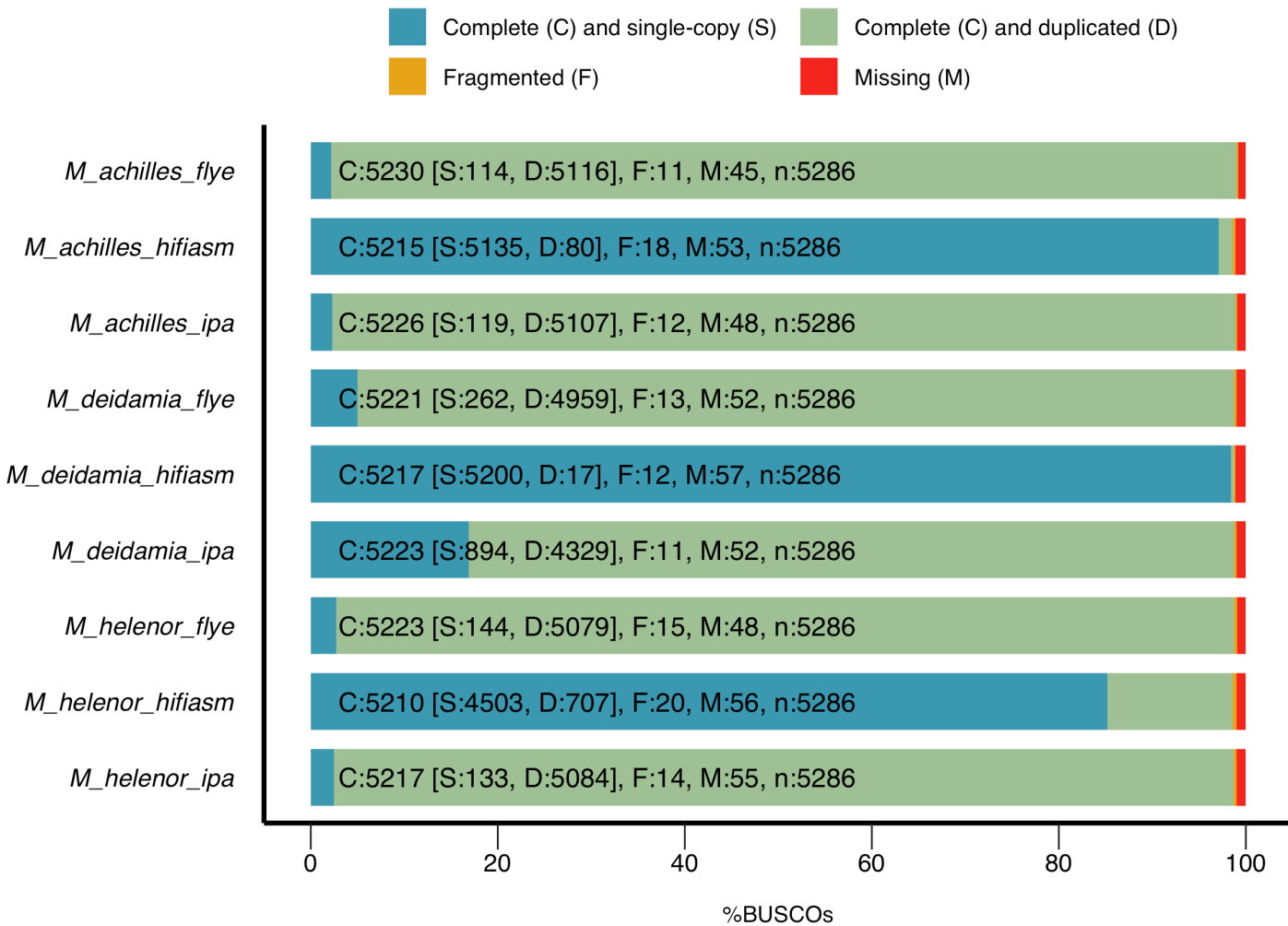

## BUSCO Assessment Results after Purge\_dups

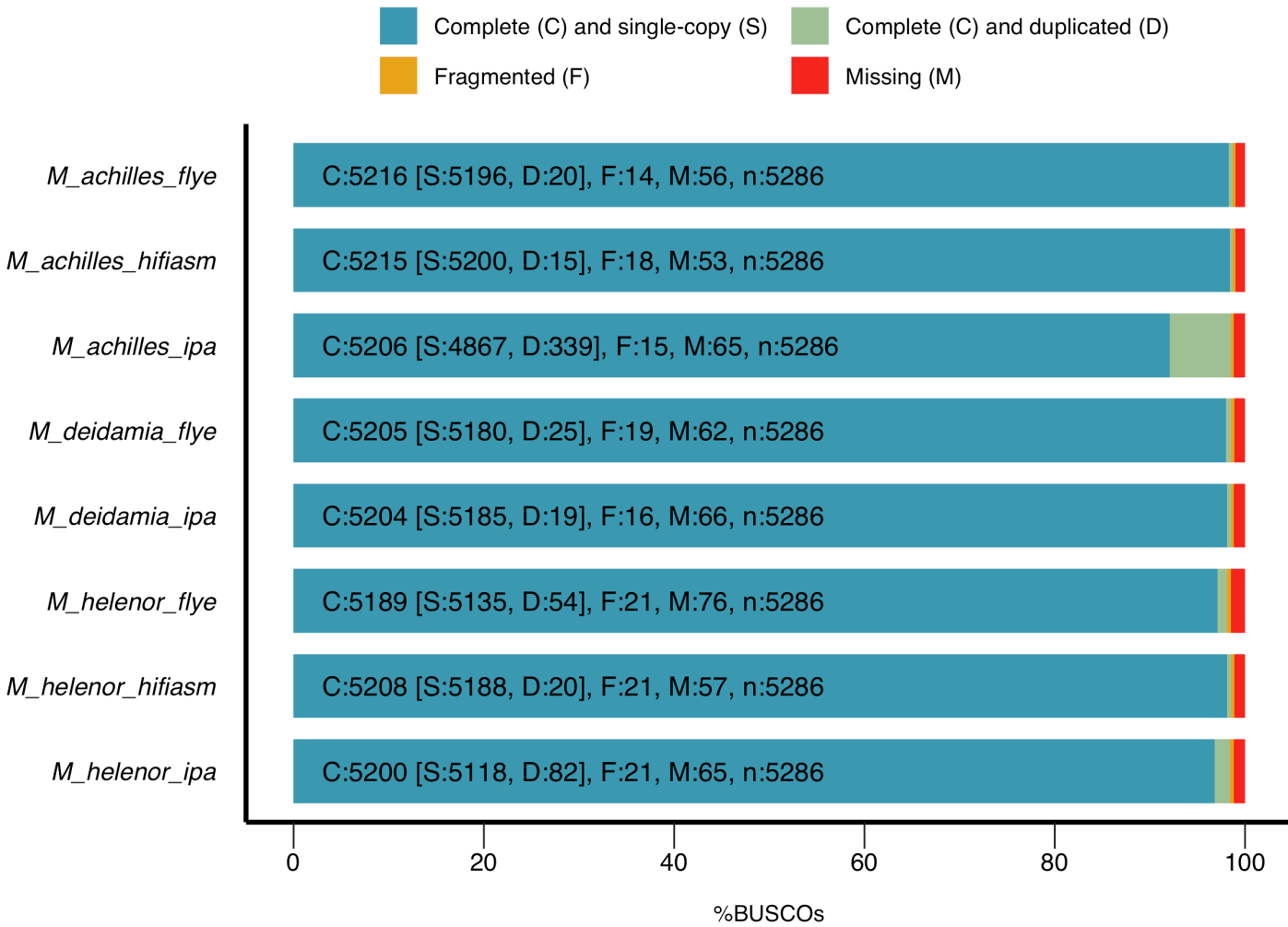

Supplement: giad033_Supplemental_Files [file giad033_supplemental_files.zip › Supp_Figure_4.pdf]

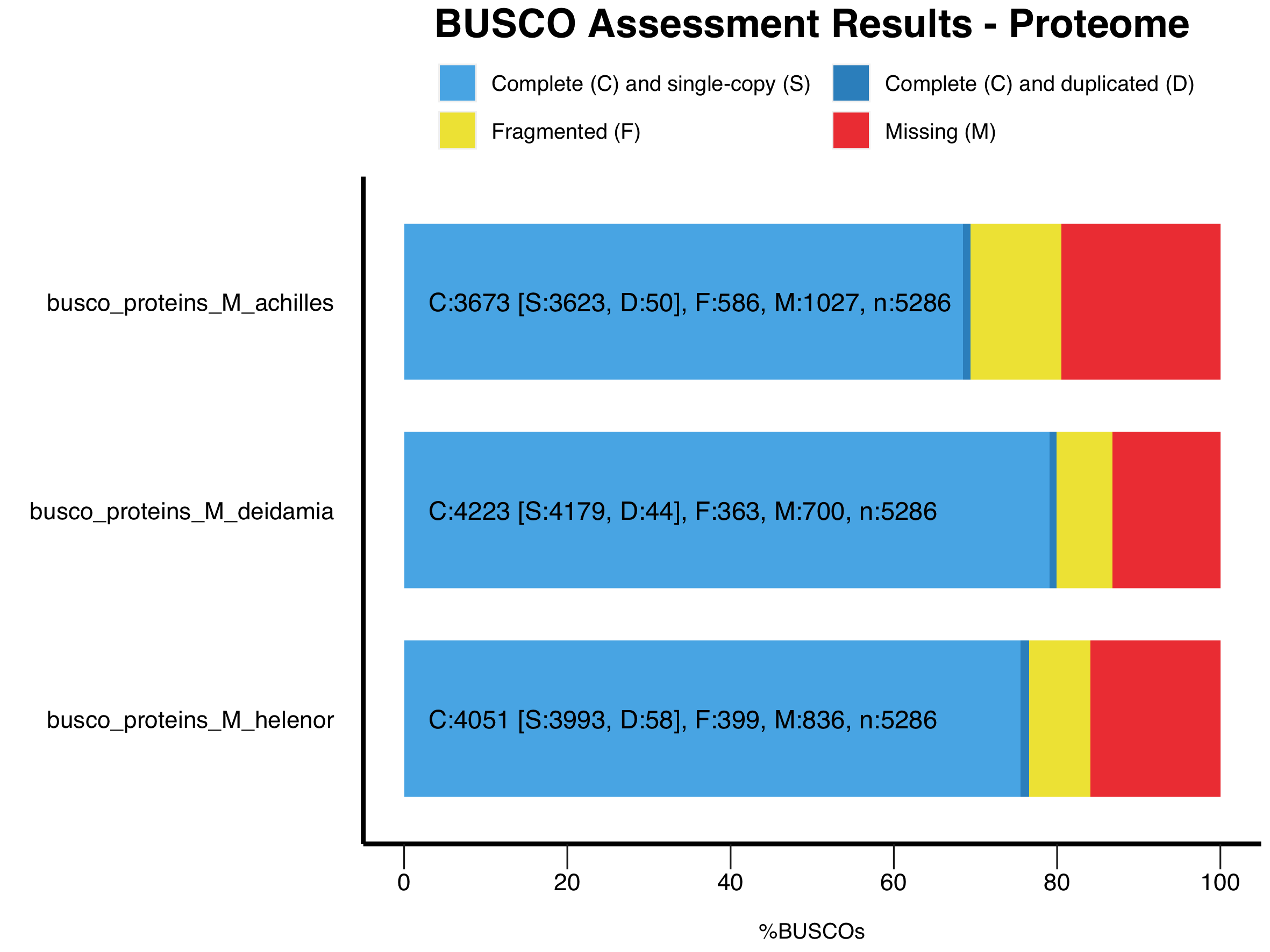

Supplement: giad033_Supplemental_Files [file giad033_supplemental_files.zip › Supp_Figure_5.png]

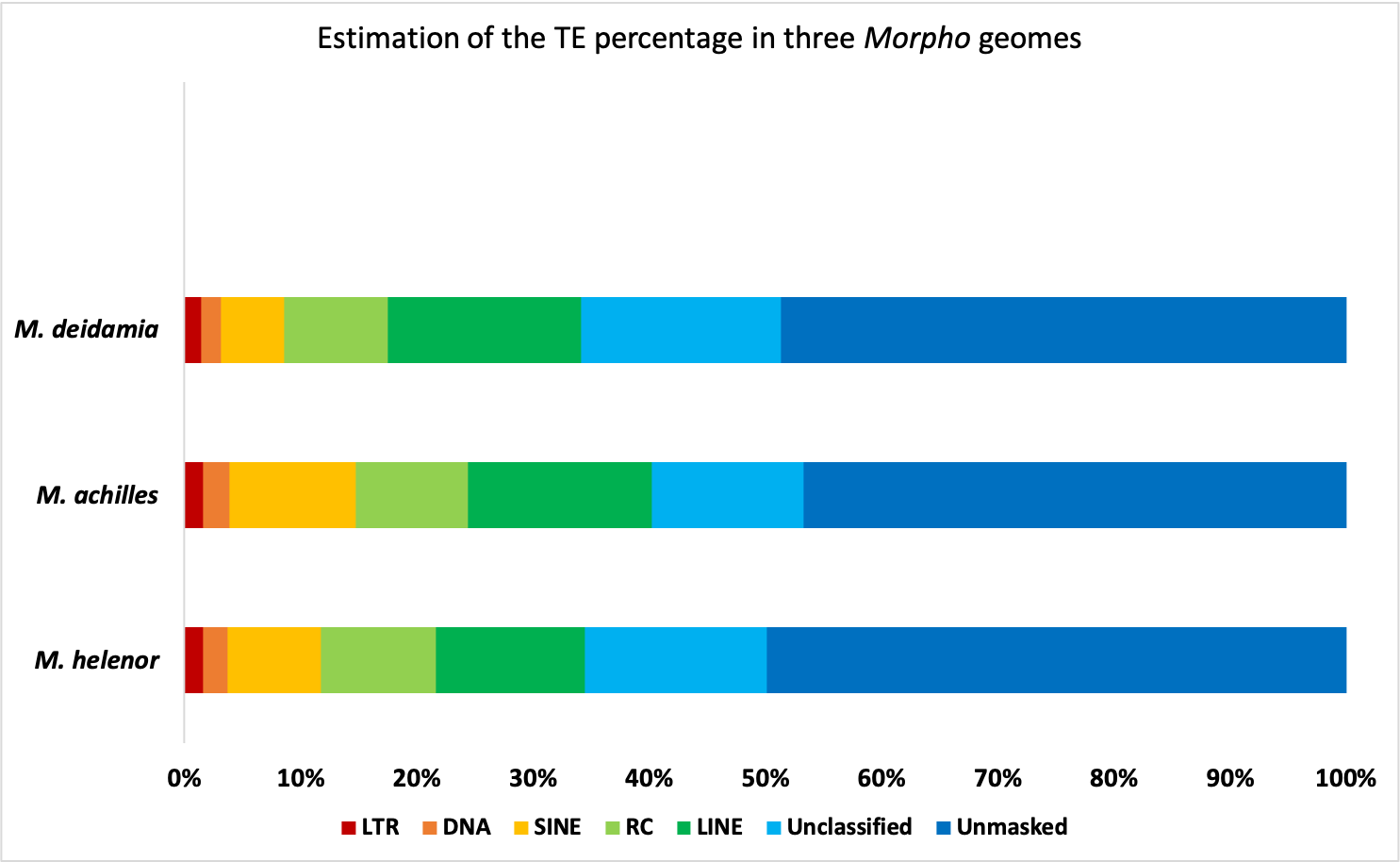

Supplement: giad033_Supplemental_Files [file giad033_supplemental_files.zip › Supp_Figure_6.png]

*M. jurtina* chromosomes

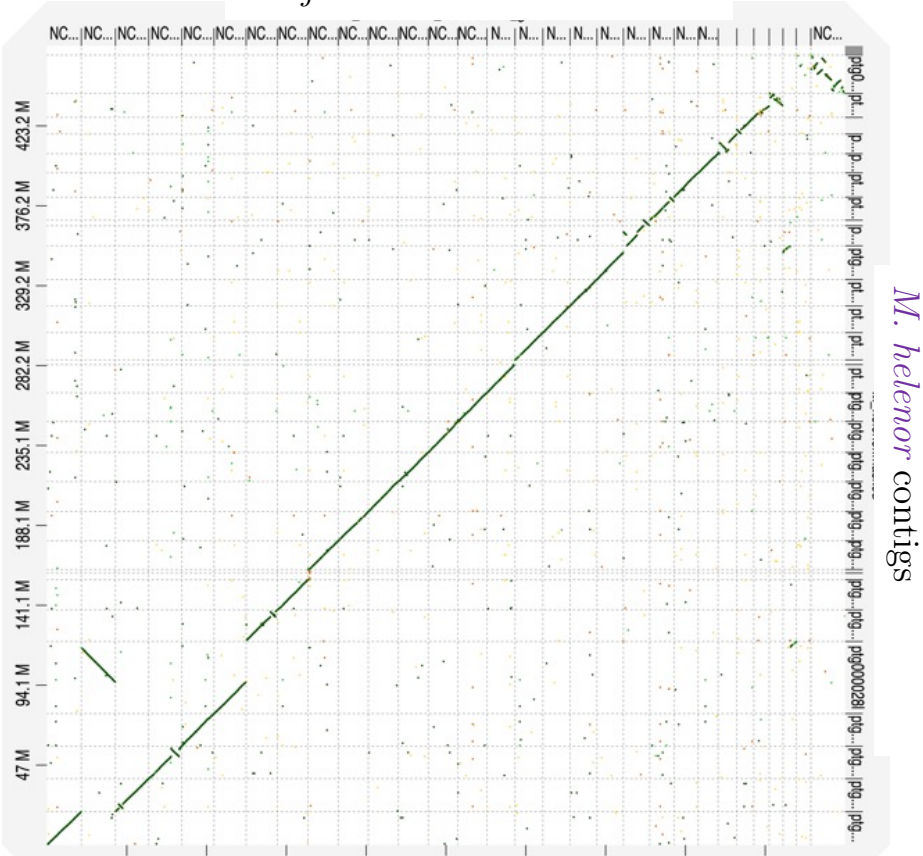

*M. helenor* contigs

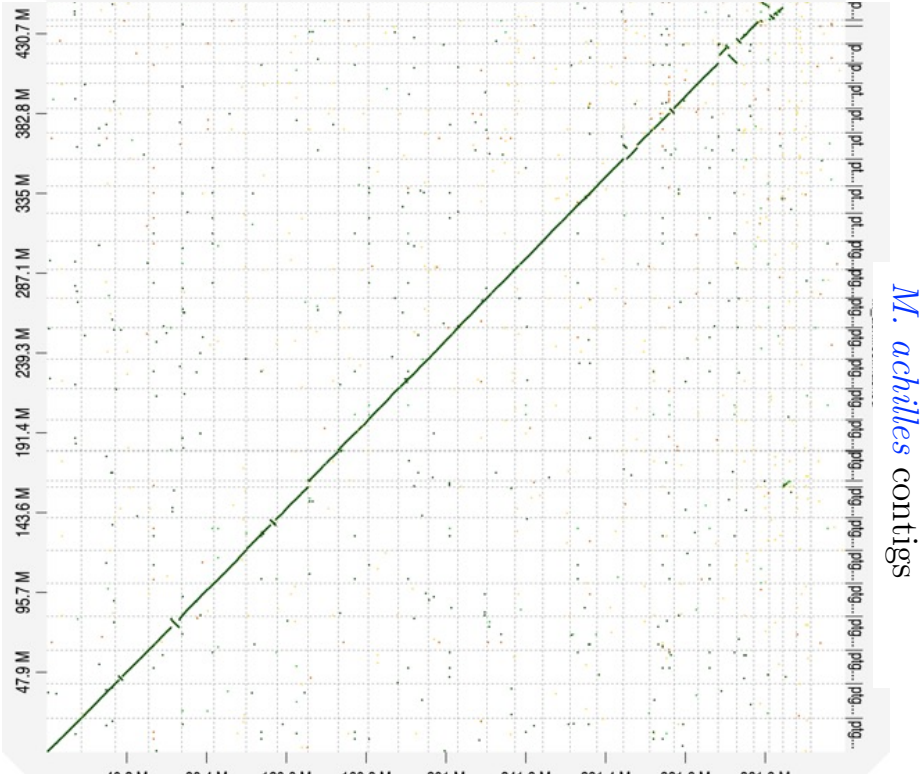

*M. achilles* contigs

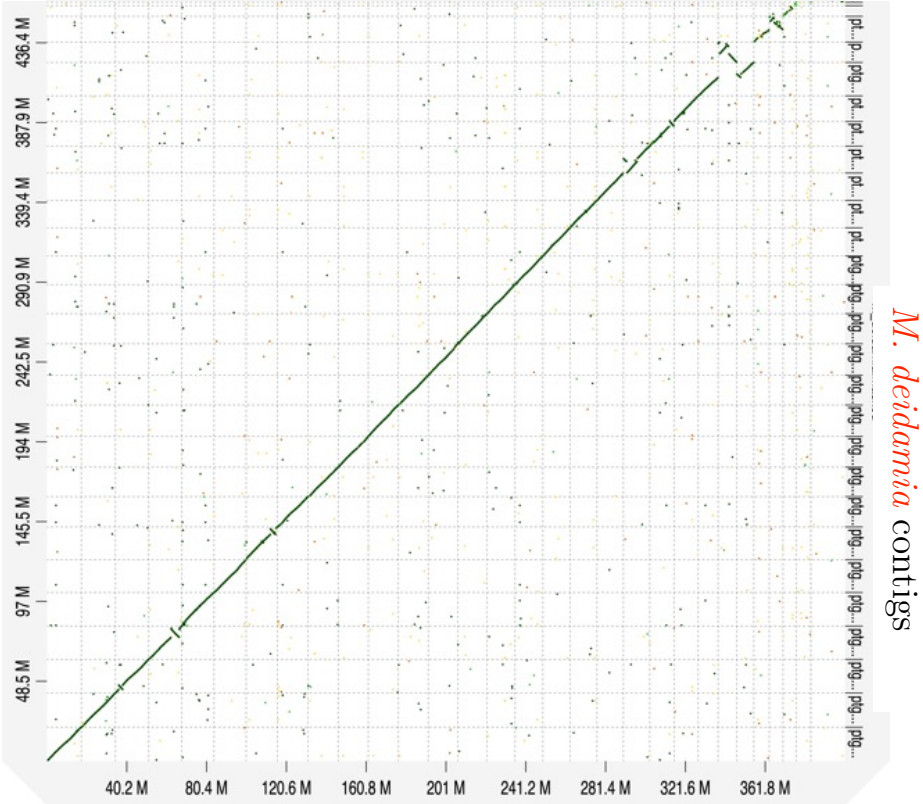

*M. deidamia* contigs

Supplement: giad033_Supplemental_Files [file giad033_supplemental_files.zip › Supp_Figure_7.pdf]

Contigs *M. helenor*

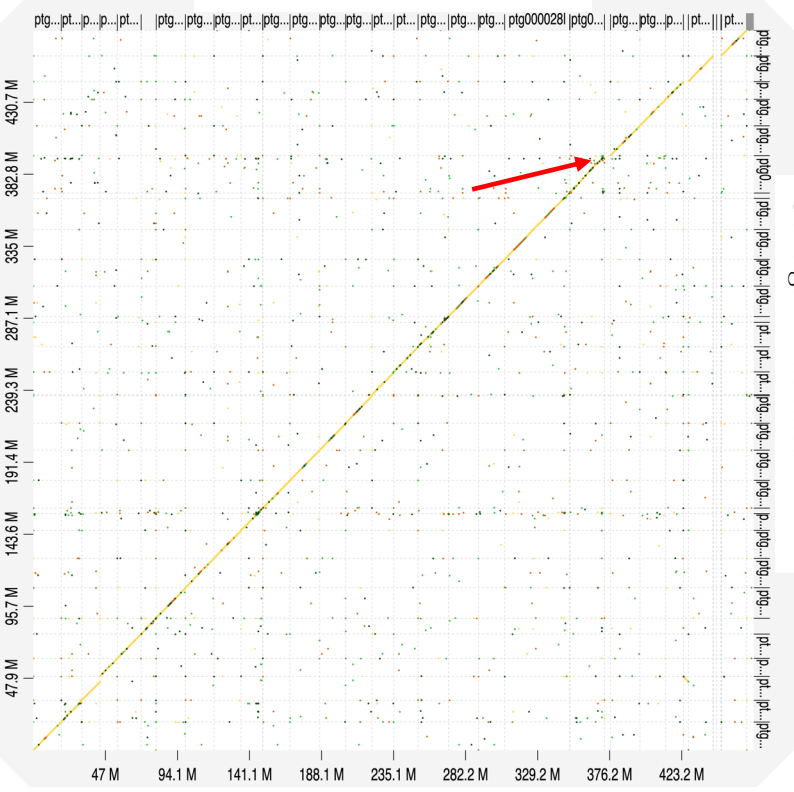

Contigs *M. helenor*

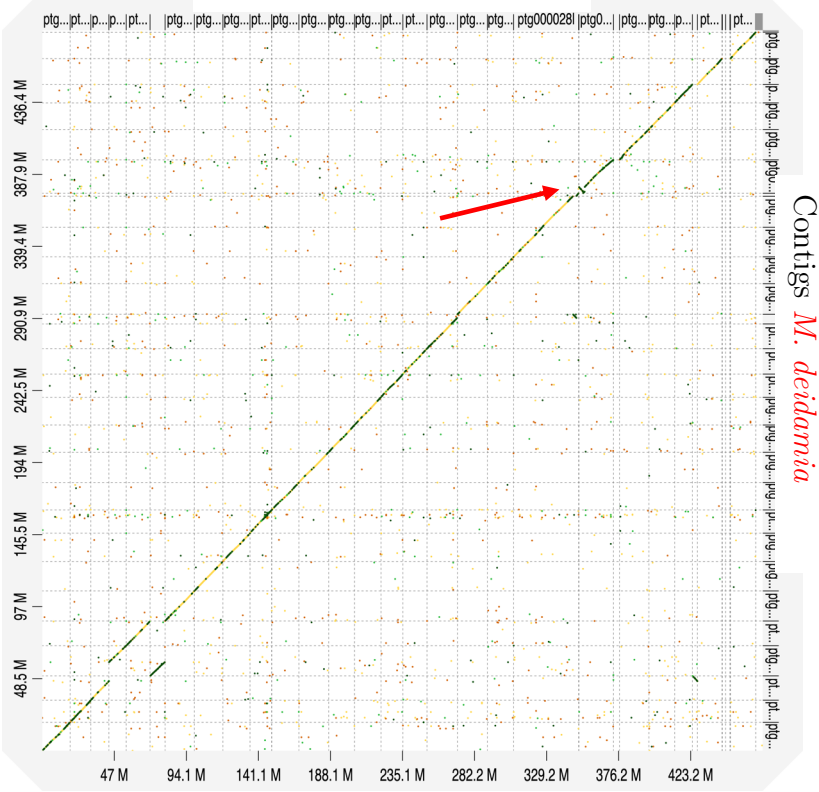

Contigs *M. achilles*

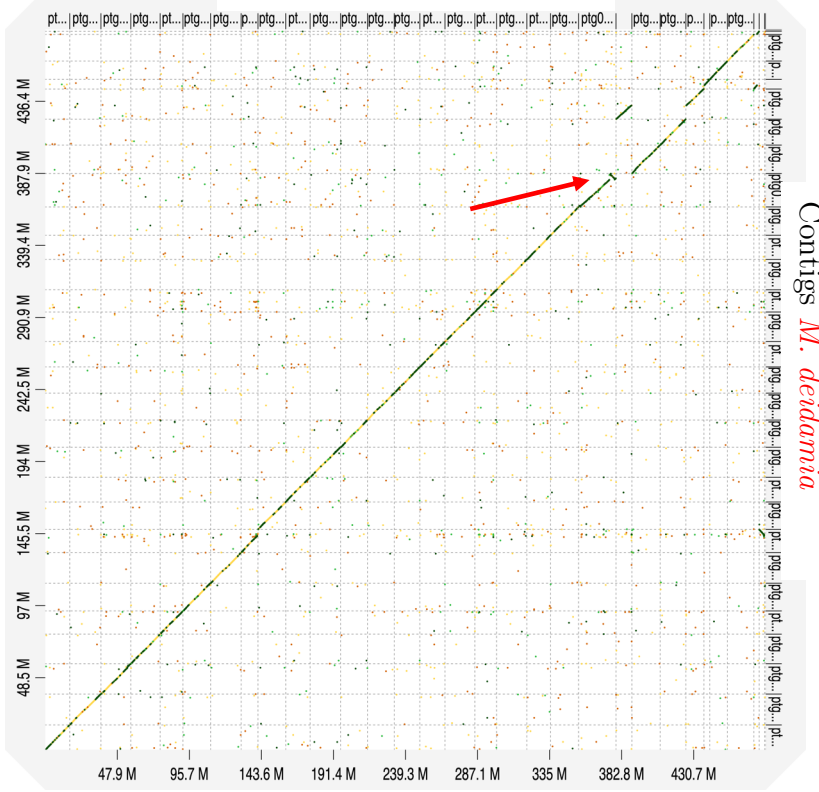

Supplement: giad033_Supplemental_Files [file giad033_supplemental_files.zip › Supp_Figure_8.pdf]

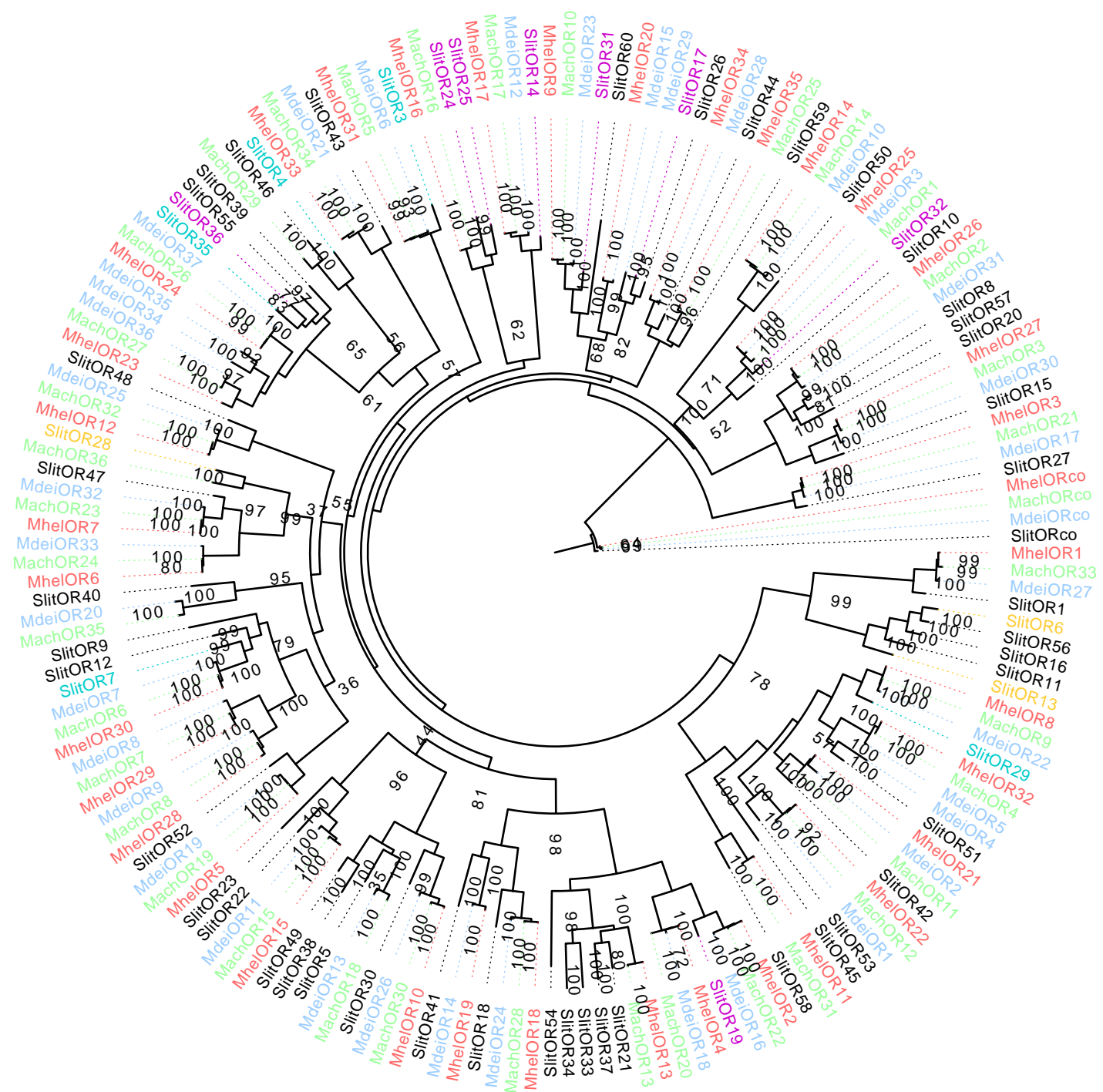

Supplement: giad033_Supplemental_Files [file giad033_supplemental_files.zip › Supp_Figure_9.pdf]
