# Supplementary material for: Genome assembly of 3 Amazonian Morpho butterfly species reveals Z-chromosome rearrangements between closely related species living in sympatry
Source: Gigascience. 2023 May 22;12:giad033. doi: 10.1093/gigascience/giad033 (PMC10202424; doi:10.1093/gigascience/giad033)
Supplement: giad033_Response_to_Reviewer_Comments_Original_Submission [file giad033_response_to_reviewer_comments_original_submission.pdf]

Author's Response To Reviewer Comments

Close

Reviewer reports:
Reviewer #1: Summary

This manuscript presents three, high-quality genomes for three charismatic butterfly species. These represent the first genon
and serve as an important resource for the community. The analyses conducted on these genomes demonstrate their value a
species as a model for exploring evolutionary processes such as speciation and exploring the role of chromosome change in s
methods are appropriate and have been carried out in a considered way.

In the most part, the manuscript clearly describes how the data was obtained and analysed. In particular, the descriptions of
sequenced, assembled and annotated are very thorough. The clarity of the text could be improved in some places. However,
overall it reads well and clearly describes how analyses were conducted and presents an interesting discussion of the results.

We thank the referees for their time and for the comments made to our manuscript. Our answers are included in the text.

The following minor comments apply to the methods:

The description of how the karyotype analyses were conducted was more limited compared to the genomic descriptions. Whil
understandable as the authors used a protocol similar to what was described in a previous study, additional details such as w
required as two were used in the cited study (McClure et al. 2017).

The Giemsa method was used to stain the Morpho chromosomes and this precision is now added to the manuscript (l. 113-11

I could not find any project records when I searched for project number PRJEB56642 on ENA. Please could the data be made
the manuscript is accepted.

The data will be released as soon as the manuscript is accepted.

Genome annotations are not currently included in the supporting information, please could they be added as part of the geno
or at the very least as a citable data object on zenodo.org etc as they serve as a valuable resource to other researchers.

The transcriptomic and proteic sequences of the putative proteins predicted by Maker along with the gff files will be available
information was added in the manuscript (l. 636-637 and l. 641-642).

In the assembly section the authors state that 'Purge_dups v1.2.5 setting the cutoffs manually'. It would be helpful to state w
cutoffs for the final analysis for reproducibility.

Done (l. 170-172).

State whether BUSCOs was run in augustus or metaeuk mode (metaeuk is the default in BUSCO v5).

Done (l. 163).

"We removed short scaffolds, short alignments and low identity alignments with the R script proposed in [34]" - did you use t
ref [34]? If so explicitly state this. In Ref [34] they state that "The alignment file was filtered to retain only those aligned seq
than 200 bp and had less than 90% identity between the two genomes in contigs that were at least 1 Mb long."

Done (l. 249-254).

Filtering of scaffolds and visualisation of synteny was achieved using two Rscripts. Currently, the references to the papers wh
originally used are given but getting from these to the actual scripts takes some effort.
For the first (filtering, ref[34]) I could find the relevant script:
https://github.com/bioinfowheat/Polygonia_calbum_genomics/blob/7c75aac624157faa3ab229e3fc1e0e315302194d/synteny/
. I think it would be helpful to include this location in the manuscript for greater visibility.
I could not find the script referenced in ref[36] for visualising synteny. If the link to this script could be found, that would als
included.

Done. The script used in this section is now included in the text (l. 246-254).

Minor comments on rest of text:

Given that the close relatedness of these three species is a key feature of this study, and is what makes this such an interest
would be helpful to mention the estimated divergence time between the species pairs from previous studies (M. helenor vs M
helenor vs M. deidamia: 11.9 mya) (Penz et al. 2012)

We agree with this relevant suggestion, and we added the divergence times estimated in the latest publication on the diversi
(Chazot et al. 2021) that slightly differ from the numbers reported in Penz et al. 2012 (l. 62-67).

While it is mentioned in the discussion that two of the three species have previously been karyotypically described, this is not
in the text. It would be good to acknowledge this previous work also in the results section briefly to state that the results her
previous work. For example here, "First, we characterized the caryotypes of the three studied species (see sup. figure 2 to vi
chromosomes)."

This is again a highly relevant comment. We now state that the modal number of chromosomes in the Morphinae is n=28 in t
2007, and that n=28 chromosomes were found in M. helenor and M. achilles in the same study, consistent with our own findi

"Chromosomal rearrangements are likely to play a major role in both adaptation and speciation processes [1]"

The reference used only references inversions, not other sources of rearrangements. Suggest using either a more general ref
De Kayne, 2021 or adding extra references for other types of rearrangements. May also be worth explicitly listing types of rea
duplications given these are a key aspect of the manuscript.

This reference has been added (l. 70).

Consider combining the last two paragraphs of the introduction which are short into one paragraph, and moving the sentence
to flow better. For example, "By obtaining karyotype data and generating whole genome sequences for three sympatric speci
we.."

We tried to combine these two short paragraphs in a single one as suggested by the referee (l. 60-78).

"Males from the species M. helenor, M. achilles and M. deidamia were caught with a handnet at the Patawa waterfall, located
of French Guiana"

Explicitly state the number per species in brackets after each species.

This sampling was performed during a mark-recapture experiments carried out in July 2019 and July 2021, where we caught
Morphos (n=395) but kept only few specimens for sequencing and karyotyping. We added the number of specimens per spec
extraction, sequencing and karyotyping in brackets in the manuscript (l. 92-93 and l. 101-102).

"To assess variations in chromosome-scale synteny, we compared the assemblies of each Morpho to the assembly of M. jurti
Morpho for which a high quality chromosome level assembly (based in N50 values and Busco score, accession ID GCF_90533

It would be helpful to state the chromosome number of M. jurtina (n=29) in this context.

Done (l. 224).

The authors state the number of M. achilles individuals which were used for karyotype analyses but do not state the number
achilles.
We used n=3 specimens of M. helenor, n=2 specimens of M. deidamia and n=4 specimens of M. achilles for karyotypic analys
now provided in the manuscript (l. 92-93).

"GenomeScope analyses suggested relatively similar genome sizes (between 470 and 489 Mb) and very high levels of hetero
species 1."

Should this say Table 1? If so, why are the genome sizes in the table different to the text (335-380 Mb in Table 1)? Values 47
final assemblies (post-purging for H. helene and H. achilles) based in Sup Tab 1.

I would suggest that Table 1 is altered to show the statistics for the final assemblies per species (haploid genome size, numb
heterozygosity, BUSCO genome complete/fragmented %). It could also include the number of predicted genes and BUSCO pr
complete/fragmented %.

We have now fused the genomescope heterozygosity results and statistics table in one single table following the suggestions
(Table 1).
We have also added the results of BUSCO run in the protein mode to get the BUSCO score of the proteome. Nevertheless, we
as figures in the supplementary material because when added to a general table, the size of the table was difficult to read (su
5).

"The use of purge_dups significantly reduced thenumber ofduplicates"

I'd say "strongly" rather than "significantly" as that can imply that statistics have been carried out.

Corrected (l. 298).

"In order to assess if the annotations were complete, we estimated in each species the percentage of proteins with a Pfam do
achilles to 71,32% in M. helenor …showing that the annotations were of good quality."

While I agree that this shows that the predicted genes are of good quality, it doesn't necessarily show how complete the anno
piece of information would be to run BUSCO in proteome mode on the annotation and compare the resulting completeness re
genome.

Done. These results have been added to the supplementary material (supplementary figure 5) and a sentence has been adde

"The phylogenetic relationships between the copies in the three species reveal that the duplications observed in the three Mo
occurred before their speciation"

I agree with the authors that this is by far the most likely explanation for the data. It might be worth saying however that the
(which is far less likely!) is that the three species share ancient haplotypic variation. It might be helpful to show Fig 2 as a tre
cladogram, thus including branch lengths to evaluate this but I don't think this is necessary.

The tree indeed shows that the level of resemblance of the opsin copy sequences among the three species was correlated wit
divergence, suggesting that theses copies were ancestral to the diversification of these three species and then evolved indepe
lineage. Nevertheless, we agree that we cannot precisely date the duplication events, we are currently aiming at detecting th
genus Morpho to infer their putative origin (within the genus Morpho or ancestral to the diversification of this genus). We thu
lack of precision we currently have on the evolutionary origin of these two duplications (l. 361-363).

"The dot-plots from the paired comparisons between the three Morpho using D-GENIES showed a very high similarity betwee
figure 7)."

The use of D-GENIES is mentioned in the methods so is not needed again in the results. Instead of stating the tool, it might b
reader to describe the analysis e.g. "pairwise comparisons of synteny…"

The sentence has been modified (l. 445-447).

"In M. deidamia, the Hifiasm assembly showed a single scaffold ptg000028l containing chromosomes NC_060051.1 and NC_(

I believe this should be ptg000008l rather than ptg000028l. It also looks like ptg0000161l is syntenic to two chromosomes in
and NC_060055.1). I

The referee is right, we have changed the sentences (l. 413-423).

Based on the synteny plots in Fig 3, it seems that after filtering, H. helenor and H. deidomia have scaffolds with synteny to 2
of M. jurtina. In contrast, H. achilles has scaffolds with synteny to all 29 chromosomes of M. jurtina. Presumably, the sequen
two missing homologous chromosomes in H. helenor and H. deidomia are in the filtered out scaffolds. It might be good to che
scaffold(s) do map to these two M. jurtina chromosomes and which filter caused them to be dropped from the analysis as oth
be interested in these. It is also a good sanity check that no data has been lost.

We think the difference in the chromosome and contig number between M. helenor and M. deidamia (27 contigs each) vs M. j
pairs) is not due to the filtering of sequences in Morpho.
In the case of M. helenor, the biggest contig in the assembly (ptg000028l with a size of 42 Mb), appears syntenic in the plot t
jurtina (chromosomes 2 and 6 with sizes of 17.19 Mb and 16.2 Mb respectively). As the number of contigs recovered by the s
is less than the number of chromosomes observed in the karyotype of this species and as the size of contig ptg000028l is twi
contig in any Morpho species we analyzed, we believe this very large contig was produced by an overassembly of two chromo
by hifiasm.
In the case of M. deidamia, Maniola's chromosomes 24 and 25 (with sizes 10.05 and 9.43 Mb respectively) appear syntenic to
deidamia (contig ptg000008l with a size of 20.29 Mb). Nevertheless, synteny plots and karyotype of this species gave the sar
contigs/chromosomes (27). We then suggest that in this case, the chromosome corresponding to the contig ptg000008l in M.
formed by fusion of two chromosomes of M. jurtina.
We have tried to clarify this in the text (l. 413-423).

In Fig 3, could the scaffolds that are described in the text (e.g. ptg000008l) be annotated in some way to allow the reader to
for example by making the text label red?

Done. We have now labeled Z chromosomes in red and single chromosomes in
Morpho that are not assigned to a single chromosome in M. jurtina in blue.

The Morpho species labels in Fig 3 and Fig4 are red, green and blue which may prove difficult for colour blind readers. It may
of these colours.

Done. We have changed the green color used in M. helenor.

Genomes on a Tree (GoaT) (https://goat.genomehubs.org/) predicts a genome size of 474 Mb for Morpho species so the valu
indeed very close to the estimate. This may be worth adding but not essential!

Done. This was added in the discussion (l. 468-470).

"The karyotype and assembly analyses suggest some differences in chromosome number between the three sympatric Morph
particularly between M. deidamia (27 chromosome pairs) and M. achilles (28 or 29 chromosome pairs)."

As in the results, M. achilles is described as having n=28 in all four specimens studied, I am not sure why "28 or 29" is stated

This sentence has been corrected (l. 523-526).

"In two ascidians species of the genus Ciona and in insects like Drosophila inversions may promote speciation by reduction of

sterility of heterozygotes."

Reference(s) missing.

The references have been added (l. 550-552).

"Figure 4. Rearrangement (SyRI) plot of the paired comparisons for the Z scaffold between the three Morpho species. Upper f
deidamia; middle: M. deidamia andM. achilles; lower: M. helenor andM. deidamia. SyRI results were plotted using plotsr"

"SYRI results were plotted using plotter" should be in methods rather than the figure legend.

This was removed from the figure caption and highlighted in the methods (l. 263-264).

Lower text should say 'M. helenor and M. achilles' rather than 'M. helenor and M. deidamia'.

Corrected.

'Synteny and rearrangement plot' is more descriptive than 'rearrangement plot'.

The sentence has been modified.

"Data show that across the Passeriformes, the Z chromosome has accumulated more inversions than any other autosome anc
fixation rate on the Z chromosome is 1.4 times greater than the average autosome."

Explicitly describe the type of data e.g. 'cytological and genomic data..'.

This has been added (l. 559).

"Further population analyses are needed to answer this question and to enlighten what evolutionary forces could be acting to
I suggest removing 'Further' as that suggests population analyses have been presented here.

Done (l. 589).

"For instance, the three copies of LW opsins found in the Papilio genus (fig.)"
I think this should read as "Fig 2"?

Corrected (l. 595).

"Interestingly, a blast of the putative proteic sequences of each Morpho species against those of M. jurtina allowed us to unco
numbers of the gene bric - a - brac, which play a significant role in differences of UV iridescence between males of two incipie
butterflies [60]."

Similar to comment 13, While a blast search is what was carried out, it would be more helpful to the reader to describe the a
used e.g. "a homology search"

Done (l. 605).

Scale bar is missing from Sup Fig 2.

The scale bar was added to Sup Fig 2.

Reviewer #2: The authors report three high quality genomes of three closely related species of Morpho butterflies. While the
normal butterfly genomes, with no surprising features, the species involved are iconic and the whole genus does not have a g
previously. For the research community, one might say finally a Morpho has been genomed! I did find it interesting that three

lower numbers of genes for the olfactory receptors, these butterflies are well known to be easy to catch in butterfly bait traps [illegible] and other smelly types of bait. Perhaps the remaining ORs are those that help them find rotting fruit in the forests?

We added a phylogenetic analysis of the OR sequences uncovered in the three Morpho species along with those of the moth S[illegible] which a number of ORs have been previously deorphanized. Our results show that the remaining ORs were not clustered arou[illegible] particular. We would thus need further functional characterization of the remaining ORs in the Morpho species to fully answer [illegible] modified the results to include this information, added a supplementary figure with the phylogenetic tree (Supplementary Fig[illegible] methods accordingly (l. 365-390 and l. 207-220 respectively).

Another interesting point made in the manuscript is that the Z chromosome might have something to do with keeping the spe[illegible] authors did not cite another paper suggesting this for Junonia butterflies (DOI: 10.1111/syen.12428), and although the quali[illegible] paper is much lower than for this manuscript, the authors of the Junonia paper do extensively discuss the role of the Z chrom[illegible] species boundaries in closely related species. Perhaps something to include in the discussion?

We added the reference in the discussion (l. 622-626).

Otherwise the paper is very well written, and the availability of these new genomes to the research community is highly excit[illegible] point to make, the GPS coordinates place the collection site in the middle of the Atlantic off the coast of West Africa, i.e. the l[illegible] 52.15832.

Corrected (l. 83-84).

Close
