# Supplementary material for: Genome assembly of 3 Amazonian Morpho butterfly species reveals Z-chromosome rearrangements between closely related species living in sympatry
Source: Gigascience. 2023 May 22;12:giad033. doi: 10.1093/gigascience/giad033 (PMC10202424; doi:10.1093/gigascience/giad033)
Supplement: giad033_Response_to_Reviewer_Comments_Revision_1 [file giad033_response_to_reviewer_comments_revision_1.pdf]

Author's Response To Reviewer Comments

Close

All required changes were made.

Close
