# Supplementary material for: Genome assembly of 3 Amazonian Morpho butterfly species reveals Z-chromosome rearrangements between closely related species living in sympatry
Source: Gigascience. 2023 May 22;12:giad033. doi: 10.1093/gigascience/giad033 (PMC10202424; doi:10.1093/gigascience/giad033)
Supplement: giad033_Reviewer_1_Report_Original_Submission — Charlotte Julie Wright, MPhil -- 12/8/2022 Reviewed [file giad033_reviewer_1_report_original_submission.pdf]

Reviewer Report

Title: Genome assembly of three Amazonian Morpho butterfly species reveals Z-chromosome rearrangements between closely-related species living in sympatry

Version: Original Submission **Date:** 12/8/2022

Reviewer name: Charlotte Julie Wright, MPhil

Reviewer Comments to Author:

Summary
This manuscript presents three, high-quality genomes for three charismatic butterfly species. These represent the first genomes for any Morpho species and serve as an important resource for the community. The analyses conducted on these genomes demonstrate their value and the use of these species as a model for exploring evolutionary processes such as speciation and exploring the role of chromosome change in such processes. The methods are appropriate and have been carried out in a considered way.
In the most part, the manuscript clearly describes how the data was obtained and analysed. In particular, the descriptions of how the genomes were sequenced, assembled and annotated are very thorough. The clarity of the text could be improved in some places. However, this is very minor and overall it reads well and clearly describes how analyses were conducted and presents an interesting discussion of the results.
The following minor comments apply to the methods:
The description of how the karyotype analyses were conducted was more limited compared to the genomic descriptions. While the brevity is understandable as the authors used a protocol similar to what was described in a previous study, additional details such as which stain was used is required as two were used in the cited study (McClure et al. 2017).
I could not find any project records when I searched for project number PRJEB56642 on ENA. Please could the data be made publicly available before the manuscript is accepted.
Genome annotations are not currently included in the supporting information, please could they be added as part of the genome assembly submissions, or at the very least as a citable data object on zenodo.org etc as they serve as a valuable resource to other researchers.
In the assembly section the authors state that 'Purge_dups v1.2.5 setting the cutoffs manually'. It would be helpful to state what values were used as cutoffs for the final analysis for reproducibility.
State whether BUSCOs was run in augustus or metaeuk mode (metaeuk is the default in BUSCO v5).
"We removed short scaffolds, short alignments and low identity alignments with the R script proposed in [34]" - did you use the same thresholds as in ref [34]? If so explicitly state this. In Ref [34] they state that "The alignment file was filtered to retain only those aligned sequences that were longer than 200â€‰bp and had less than 90% identity between the two genomes in contigs that were at least 1â€‰Mb long."
Filtering of scaffolds and visualisation of synteny was achieved using two Rscripts. Currently, the references to the papers where the Rscripts were originally used are given but getting from these to the

actual scripts takes some effort.
For the first (filtering, ref[34]) I could find the relevant script:
https://github.com/bioinfowheat/Polygonia_calbum_genomics/blob/7c75aac624157faa3ab229e3fc1e0e315302194d/synteny/circlePlot_nucmerOutput.R . I think it would be helpful to include this location in the manuscript for greater visibility.
I could not find the script referenced in ref[36] for visualising synteny. If the link to this script could be found, that would also be beneficial to be included.
Minor comments on rest of text:
Given that the close relatedness of these three species is a key feature of this study, and is what makes this such an interesting system, I think it would be helpful to mention the estimated divergence time between the species pairs from previous studies (M. helenor vs M. achilles: 6.8 mya; M. helenor vs M. deidamia: 11.9 mya) (Penz et al. 2012)
While it is mentioned in the discussion that two of the three species have previously been karyotypically described, this is not mentioned anywhere else in the text. It would be good to acknowledge this previous work also in the results section briefly to state that the results here are consistent with previous work. For example here, "First, we characterized the caryotypes of the three studied species (see sup. figure 2 to visualize the chromosomes)."
"Chromosomal rearrangements are likely to play a major role in both adaptation and speciation processes [1]"
The reference used only references inversions, not other sources of rearrangements. Suggest using either a more general reference such as Feulner & De Kayne, 2021 or adding extra references for other types of rearrangements. May also be worth explicitly listing types of rearrangements such as duplications given these are a key aspect of the manuscript.
Consider combining the last two paragraphs of the introduction which are short into one paragraph, and moving the sentences around to allow the text to flow better. For example, "By obtaining karyotype data and generating whole genome sequences for three sympatric species of Morpho buttterflies, we.."
"Males from the species M. helenor, M. achilles and M. deidamia were caught with a handnet at the Patawa waterfall, located in the Kaw mountain area of French Guiana"
Explicitly state the number per species in brackets after each species.
"To assess variations in chromosome-scale synteny, we compared the assemblies of each Morpho to the assembly of M. jurtina, the closest relative of Morpho for which a high quality chromosome level assembly (based in N50 values and Busco score, accession ID GCF_905333055.1) is available [27]."
It would be helpful to state the chromosome number of M. jurtina (n=29) in this context.
The authors state the number of M. achilles individuals which were used for karyotype analyses but do not state the number for M. helenor or M. achilles.
"GenomeScope analyses suggested relatively similar genome sizes (between 470 and 489 Mb) and very high levels of heterozygosity for the three species 1."
Should this say Table 1? If so, why are the genome sizes in the table different to the text (335-380 Mb in Table 1)? Values 470-489 Mb correspond to final assemblies (post-purging for H. helene and H. achilles) based in Sup Tab 1.
I would suggest that Table 1 is altered to show the statistics for the final assemblies per species (haploid genome size, number of scaffolds, N50, heterozygosity, BUSCO genome complete/fragmented %). It

could also include the number of predicted genes and BUSCO proteome complete/fragmented %.
"The use of purge_dups significantly reduced thenumber ofduplicates"
I'd say "strongly" rather than "significantly" as that can imply that statistics have been carried out.
"In order to assess if the annotations were complete, we estimated in each species the percentage of proteins with a Pfam domain … 65,50% in M. achilles to 71,32% in M. helenor …showing that the annotations were of good quality."
While I agree that this shows that the predicted genes are of good quality, it doesn't necessarily show how complete the annotation is. A quick extra piece of information would be to run BUSCO in proteome mode on the annotation and compare the resulting completeness result to that of the genome.
"The phylogenetic relationships between the copies in the three species reveal that the duplications observed in the three Morpho species probably occurred before their speciation"
I agree with the authors that this is by far the most likely explanation for the data. It might be worth saying however that the alternative explanation (which is far less likely!) is that the three species share ancient haplotypic variation. It might be helpful to show Fig 2 as a tree rather than a cladogram, thus including branch lengths to evaluate this but I don't think this is necessary.
"The dot-plots from the paired comparisons between the three Morpho using D-GENIES showed a very high similarity between genomes (see sup. figure 7)."
The use of D-GENIES is mentioned in the methods so is not needed again in the results. Instead of stating the tool, it might be more helpful to the reader to describe the analysis e.g. "pairwise comparisons of synteny…"
"In M. deidamia, the Hifiasm assembly showed a single scaffold ptg000028l containing chromosomes NC_060051.1 and NC_060052.1 from M. jurtina."
I believe this should be ptg000008l rather than ptg000028l. It also looks like ptg0000161l is syntenic to two chromosomes in M. jurtina (NC_060054.1 and NC_060055.1). I
Based on the synteny plots in Fig 3, it seems that after filtering, H. helenor and H. deidomia have scaffolds with synteny to 27 of the 29 chromosomes of M. jurtina. In contrast, H. achilles has scaffolds with synteny to all 29 chromosomes of M. jurtina. Presumably, the sequences corresponding to the two missing homologous chromosomes in H. helenor and H. deidomia are in the filtered out scaffolds. It might be good to check this and report which scaffold(s) do map to these two M. jurtina chromosomes and which filter caused them to be dropped from the analysis as other users of this data may be interested in these. It is also a good sanity check that no data has been lost.
In Fig 3, could the scaffolds that are described in the text (e.g. ptg000008l) be â€‹â€‹annotated in some way to allow the reader to more easily locate them, for example by making the text label red?
The Morpho species labels in Fig 3 and Fig4 are red, green and blue which may prove difficult for colour blind readers. It may be helpful to change one of these colours.
Genomes on a Tree (GoaT) (https://goat.genomehubs.org/) predicts a genome size of 474 Mb for Morpho species so the values obtained here are indeed very close to the estimate. This may be worth adding but not essential!
"The karyotype and assembly analyses suggest some differences in chromosome number between the three sympatric Morpho species studied here, particularly between M. deidamia (27 chromosome pairs) and M. achilles (28 or 29 chromosome pairs)."
As in the results, M. achilles is described as having n=28 in all four specimens studied, I am not sure why

"28 or 29" is stated here.
"In two ascidians species of the genus Ciona and in insects like Drosophila inversions may promote speciation by reduction of the fitness or by causing sterility of heterozygotes."
Reference(s) missing.
"Figure 4. Rearrangement (SyRI) plot of the paired comparisons for the Z scaffold between the three Morpho species. Upper figure: M. helenor andM. deidamia; middle: M. deidamia andM. achilles; lower: M. helenor andM. deidamia. SyRI results were plotted using plotsr"
"SYRI results were plotted using plotter" should be in methods rather than the figure legend.
Lower text should say 'M. helenor and M. achilles' rather than 'M. helenor and M. deidamia'.
'Synteny and rearrangement plot' is more descriptive than 'rearrangement plot'.
"Data show that across the Passeriformes, the Z chromosome has accumulated more inversions than any other autosome and that the inversion fixation rate on the Z chromosome is 1.4 times greater than the average autosome."
Explicitly describe the type of data e.g. 'cytological and genomic data..'.
"Further population analyses are needed to answer this question and to enlighten what evolutionary forces could be acting to maintain them."
I suggest removing 'Further' as that suggests population analyses have been presented here.
"For instance, the three copies of LW opsins found in the Papilio genus (fig.)"
I think this should read as "Fig 2"?
"Interestingly, a blast of the putative proteic sequences of each Morpho species against those of M. jurtina allowed us to uncover different copy numbers of the gene bric - a - brac, which play a significant role in differences of UV iridescence between males of two incipient species of sulphur butterflies [60]."
Similar to comment 13, While a blast search is what was carried out, it would be more helpful to the reader to describe the analysis rather than the tool used e.g. "a homology search"
Scale bar is missing from Sup Fig 2.

Methods

Are the methods appropriate to the aims of the study, are they well described, and are necessary controls included? Choose an item.

Conclusions

Are the conclusions adequately supported by the data shown? Choose an item.

Reporting Standards

Does the manuscript adhere to the journal’s guidelines on minimum standards of reporting? Choose an item.

Choose an item.

Statistics

Are you able to assess all statistics in the manuscript, including the appropriateness of statistical tests used? Choose an item.

Quality of Written English

Please indicate the quality of language in the manuscript: Choose an item.

Declaration of Competing Interests

Please complete a declaration of competing interests, considering the following questions:

- Have you in the past five years received reimbursements, fees, funding, or salary from an organisation that may in any way gain or lose financially from the publication of this manuscript, either now or in the future?
- Do you hold any stocks or shares in an organisation that may in any way gain or lose financially from the publication of this manuscript, either now or in the future?
- Do you hold or are you currently applying for any patents relating to the content of the manuscript?
- Have you received reimbursements, fees, funding, or salary from an organization that holds or has applied for patents relating to the content of the manuscript?
- Do you have any other financial competing interests?
- Do you have any non-financial competing interests in relation to this paper?

If you can answer no to all of the above, write 'I declare that I have no competing interests' below. If your reply is yes to any, please give details below.

I declare that I have no competing interests.

I agree to the open peer review policy of the journal. I understand that my name will be included on my report to the authors and, if the manuscript is accepted for publication, my named report including any attachments I upload will be posted on the website along with the authors' responses. I agree for my report to be made available under an Open Access Creative Commons CC-BY license (http://creativecommons.org/licenses/by/4.0/). I understand that any comments which I do not wish to be included in my named report can be included as confidential comments to the editors, which will not be published.

Choose an item.

To further support our reviewers, we have joined with Publons, where you can gain additional credit to further highlight your hard work (see: https://publons.com/journal/530/gigascience). On publication of this paper, your review will be automatically added to Publons, you can then choose whether or not to claim your Publons credit. I understand this statement.

Yes Choose an item.
