# Supplementary material for: Genome assembly of 3 Amazonian Morpho butterfly species reveals Z-chromosome rearrangements between closely related species living in sympatry
Source: Gigascience. 2023 May 22;12:giad033. doi: 10.1093/gigascience/giad033 (PMC10202424; doi:10.1093/gigascience/giad033)
Supplement: giad033_Reviewer_2_Report_Original_Submission — Niklas Wahlberg -- 12/15/2022 Reviewed [file giad033_reviewer_2_report_original_submission.pdf]

Reviewer Report

Title: Genome assembly of three Amazonian Morpho butterfly species reveals Z-chromosome rearrangements between closely-related species living in sympatry

Version: Original Submission **Date:** 12/15/2022

Reviewer name: Niklas Wahlberg

Reviewer Comments to Author:

The authors report three high quality genomes of three closely related species of Morpho butterflies. While the genomes appear to be normal butterfly genomes, with no surprising features, the species involved are iconic and the whole genus does not have a genome available previously. For the research community, one might say finally a Morpho has been genomed! I did find it interesting that three species seem to have lower numbers of genes for the olfactory receptors, these butterflies are well known to be easy to catch in butterfly bait traps baited with rotting fruit and other smelly types of bait. Perhaps the remaining ORs are those that help them find rotting fruit in the forests? Another interesting point made in the manuscript is that the Z chromosome might have something to do with keeping the species separate. The authors did not cite another paper suggesting this for Junonia butterflies (DOI: 10.1111/syen.12428), and although the quality of data for that paper is much lower than for this manuscript, the authors of the Junonia paper do extensively discuss the role of the Z chromosome in maintaining species boundaries in closely related species. Perhaps something to include in the discussion? Otherwise the paper is very well written, and the availability of these new genomes to the research community is highly exciting. I have only one minor point to make, the GPS coordinates place the collection site in the middle of the Atlantic off the coast of West Africa, i.e. the longitude should -52.15832.

Methods

Are the methods appropriate to the aims of the study, are they well described, and are necessary controls included? Choose an item.

Conclusions

Are the conclusions adequately supported by the data shown? Choose an item.

Reporting Standards

Does the manuscript adhere to the journal's guidelines on minimum standards of reporting? Choose an item.

Choose an item.

Statistics

Are you able to assess all statistics in the manuscript, including the appropriateness of statistical tests used? Choose an item.

Quality of Written English

Please indicate the quality of language in the manuscript: Choose an item.

Declaration of Competing Interests

Please complete a declaration of competing interests, considering the following questions:

- Have you in the past five years received reimbursements, fees, funding, or salary from an organisation that may in any way gain or lose financially from the publication of this manuscript, either now or in the future?
- Do you hold any stocks or shares in an organisation that may in any way gain or lose financially from the publication of this manuscript, either now or in the future?
- Do you hold or are you currently applying for any patents relating to the content of the manuscript?
- Have you received reimbursements, fees, funding, or salary from an organization that holds or has applied for patents relating to the content of the manuscript?
- Do you have any other financial competing interests?
- Do you have any non-financial competing interests in relation to this paper?

If you can answer no to all of the above, write 'I declare that I have no competing interests' below. If your reply is yes to any, please give details below.

I declare that I have no competing interests

I agree to the open peer review policy of the journal. I understand that my name will be included on my report to the authors and, if the manuscript is accepted for publication, my named report including any attachments I upload will be posted on the website along with the authors' responses. I agree for my report to be made available under an Open Access Creative Commons CC-BY license (http://creativecommons.org/licenses/by/4.0/). I understand that any comments which I do not wish to be included in my named report can be included as confidential comments to the editors, which will not be published.

Choose an item.

To further support our reviewers, we have joined with Publons, where you can gain additional credit to further highlight your hard work (see: https://publons.com/journal/530/gigascience). On publication of this paper, your review will be automatically added to Publons, you can then choose whether or not to claim your Publons credit. I understand this statement.

Yes Choose an item.
